# YAP/TAZ mediates resistance to KRAS inhibitors through inhibiting proapoptosis and activating the SLC7A5/mTOR axis

**DOI:** 10.1172/jci.insight.178535

**Published:** 2024-12-20

**Authors:** Wang Yang, Ming Zhang, Tian-Xing Zhang, Jia-Hui Liu, Man-Wei Hao, Xu Yan, Haicheng Gao, Qun-Ying Lei, Jiuwei Cui, Xin Zhou

**Affiliations:** 1 Cancer Center, and; 2 Cancer Research Institute of Jilin University, The First Hospital of Jilin University, Changchun, China.; 3 International Center of Future Science, and; 4 School of Pharmaceutical Sciences, Jilin University, Changchun, China.; 5 Pathological Diagnostic Center, The First Hospital of Jilin University, Changchun, China.; 6 Fudan University Shanghai Cancer Center & Institutes of Biomedical Sciences, School of Basic Medical Sciences, Cancer Institutes, Key Laboratory of Breast Cancer in Shanghai, Shanghai Key Laboratory of Radiation Oncology, The Shanghai Key Laboratory of Medical Epigenetics, Shanghai Medical College, Fudan University, Shanghai, China.; 7 Department of Oncology, Shanghai Medical College, Fudan University, Shanghai, China.; 8 State Key Laboratory of Medical Neurobiology, Fudan University, Shanghai, China.

**Keywords:** Oncology, Therapeutics, Apoptosis survival pathways, Drug screens, Drug therapy

## Abstract

*KRAS* mutations are frequent in various human cancers. The development of selective inhibitors targeting KRAS mutations has opened a new era for targeted therapy. However, intrinsic and acquired resistance to these inhibitors remains a major challenge. Here, we found that cancer cells resistant to KRAS G12C inhibitors also display cross-resistance to other targeted therapies, such as inhibitors of RTKs or SHP2. Transcriptomic analyses revealed that the Hippo-YAP/TAZ pathway is activated in intrinsically resistant and acquired-resistance cells. Constitutive activation of YAP/TAZ conferred resistance to KRAS G12C inhibitors, while knockdown of YAP/TAZ or TEADs sensitized resistant cells to these inhibitors. This scenario was also observed in KRAS G12D–mutant cancer cells. Mechanistically, YAP/TAZ protects cells from KRAS inhibitor–induced apoptosis by downregulating the expression of proapoptotic genes such as *BMF*, *BCL2L11*, and *PUMA*, and YAP/TAZ reverses KRAS inhibitor–induced proliferation retardation by activating the SLC7A5/mTORC1 axis. We further demonstrated that dasatinib and MYF-03-176 notably enhance the efficacy of KRAS inhibitors by reducing SRC kinase activity and TEAD activity. Overall, targeting the Hippo-YAP/TAZ pathway has the potential to overcome resistance to KRAS inhibitors.

## Abstract 2

Targeting the Hippo signaling pathway to overcome intrinsic and acquired resistance to KRAS-targeted therapy in cancer.

## Graphical abstract

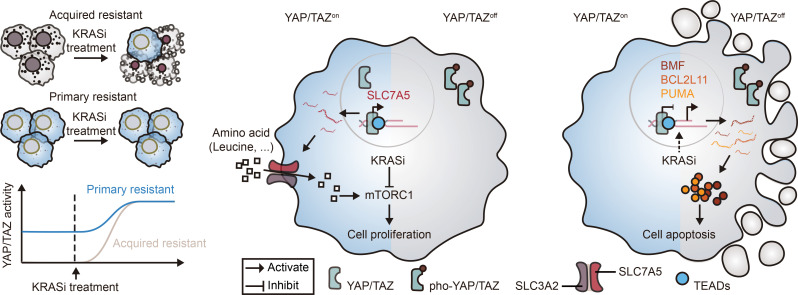

## Introduction

Kirsten rat sarcoma viral oncogene homolog (*KRAS*) is the most frequently mutated oncogene in various types of human cancers such as pancreatic, lung, and colorectal cancer, with G12, G13, and Q61 being the most prevalent mutant codons observed (1, 2). *KRAS* mutations lead to the accumulation of active GTP-bound KRAS, which in turn regulates multiple oncogenic cellular processes through its downstream mitogen-activated protein kinase (MAPK) and phosphoinositide 3-kinase (PI3K) pathways (1, 2). The perception of KRAS as “undruggable” was shattered with the discovery of selective KRAS G12C inhibitors and the subsequent clinical approval of sotorasib and adagrasib in 2021 and 2022 (3–7). This milestone represents a substantial breakthrough in precision oncology, paving the way for future targeting of other KRAS mutations.

The discovery of KRAS G12C inhibitors raises great excitement; nevertheless, intrinsic and acquired resistance to sotorasib and adagrasib has been reported in preclinical studies and clinical trials (8–10). It is believed that this resistance can arise from a variety of mechanisms, including the emergence of additional mutations in *KRAS* or other genes involved in the RAS pathway, non-mutational activation of bypass signaling pathways such as the MAPK or PI3K pathways, and diverse remodeling of the tumor microenvironment (8–10). The development of resistance to KRAS G12C inhibitors remains a major obstacle in treating KRAS-driven cancers. Several studies have explored combination strategies that involve cotargeting KRAS G12C with upstream regulators such as receptor tyrosine kinases (RTKs) and protein tyrosine phosphatase non-receptor type 11 (SHP2), or downstream effectors including mitogen-activated protein kinase kinase (MEK) and PI3K, of the RAS or other complementary pathways (7, 9, 11–13). However, cells with varying sensitivity to KRAS inhibitors exhibit inconsistent responses to these concurrent treatments (12, 14). For example, in the ClinicalTrials.gov cohort NCT04185883, although the combination of sotorasib and SHP2 inhibitor demonstrated a synergistic effect in treatment-naive patients harboring the KRAS G12C mutation, the efficacy of this combination was less pronounced in the population that had previously received KRAS G12C inhibitors (15). Therefore, there is an urgent need to identify novel targets that can generate broad efficacy in sensitizing resistant cells to KRAS inhibitors.

The Hippo pathway is critical in controlling organ size and carcinogenesis (16, 17). It consists of a kinase cascade and a transcription factor core. When activated, Ste20-like protein kinase 1 and 2 (MST1/2) phosphorylate and activate large tumor suppressor kinase 1 and 2 (LATS1/2), which then phosphorylate Yes-associated transcription regulator (YAP)/transcription coactivator with PDZ-binding motif (TAZ). This leads to the cytoplasmic accumulation and eventual degradation of YAP/TAZ (18–20). Non-phosphorylated YAP and TAZ translocate into the nucleus where they act as transcription coactivators of the transcriptional enhanced associate domain (TEAD) family of transcription factors to regulate cell proliferation, migration, epithelial-mesenchymal transition, and survival (21, 22). Emerging evidence suggests that dysregulation of the Hippo-YAP/TAZ pathway is a central resistance mechanism in various tumors to multiple targeted therapies, chemotherapies, hormonal therapies, and immunotherapies (23–27). Particularly, YAP/TAZ plays a crucial role in the intrinsic and acquired resistance to inhibitors targeting RTK/MAPK pathway (24, 28–32).

In this study, we observed that cancer cells resistant to KRAS G12C inhibitors also display cross-resistance to other targeted therapies such as inhibitors of epidermal growth factor receptor (EGFR), insulin-like growth factor 1 receptor (IGF1R), MEK, PI3K, or SHP2. We identified the YAP-TAZ-TEAD complex as a major transcription core that potentially enables the bypass of KRAS G12C inhibition. YAP and TAZ are sufficient to acquire resistance and essential for sustaining resistance to 3 tested KRAS G12C inhibitors in multiple intrinsically resistant and acquired-resistance cell lines. This scenario was also observed in KRAS G12D–mutant pancreatic cancer cells, suggesting the general role of the Hippo-YAP/TAZ pathway in resistance to KRAS inhibitors. Mechanistically, upon treatment with KRAS inhibitors, YAP/TAZ prevents apoptosis by downregulating proapoptosis genes Bcl2 modifying factor (*BMF*), Bcl-2-like protein 11 (*BCL2L11*), and BCL2 binding component 3 (*PUMA*), and sustains proliferation via the solute carrier family 7 member 5 (SLC7A5)/mTORC1 axis. By screening a panel of compounds targeting the Hippo-YAP/TAZ pathway, we identified that dasatinib and MYF-03-176 remarkably enhanced the efficacy of both KRAS G12C inhibitors and a KRAS G12D inhibitor. Taken together, our data reveal that the Hippo-YAP/TAZ pathway is critical in conferring and sustaining resistance to KRAS inhibitors, and modulation of this pathway holds promise for overcoming resistance to KRAS inhibitors and improving outcomes for patients with KRAS-mutant cancers.

## Results

### KRAS G12C–mutant cancer cell lines with either intrinsic or acquired resistance to KRAS G12C inhibitors display cross-resistance to other inhibitors targeting upstream or downstream components of the RAS signaling pathway.

**Figure 1:**
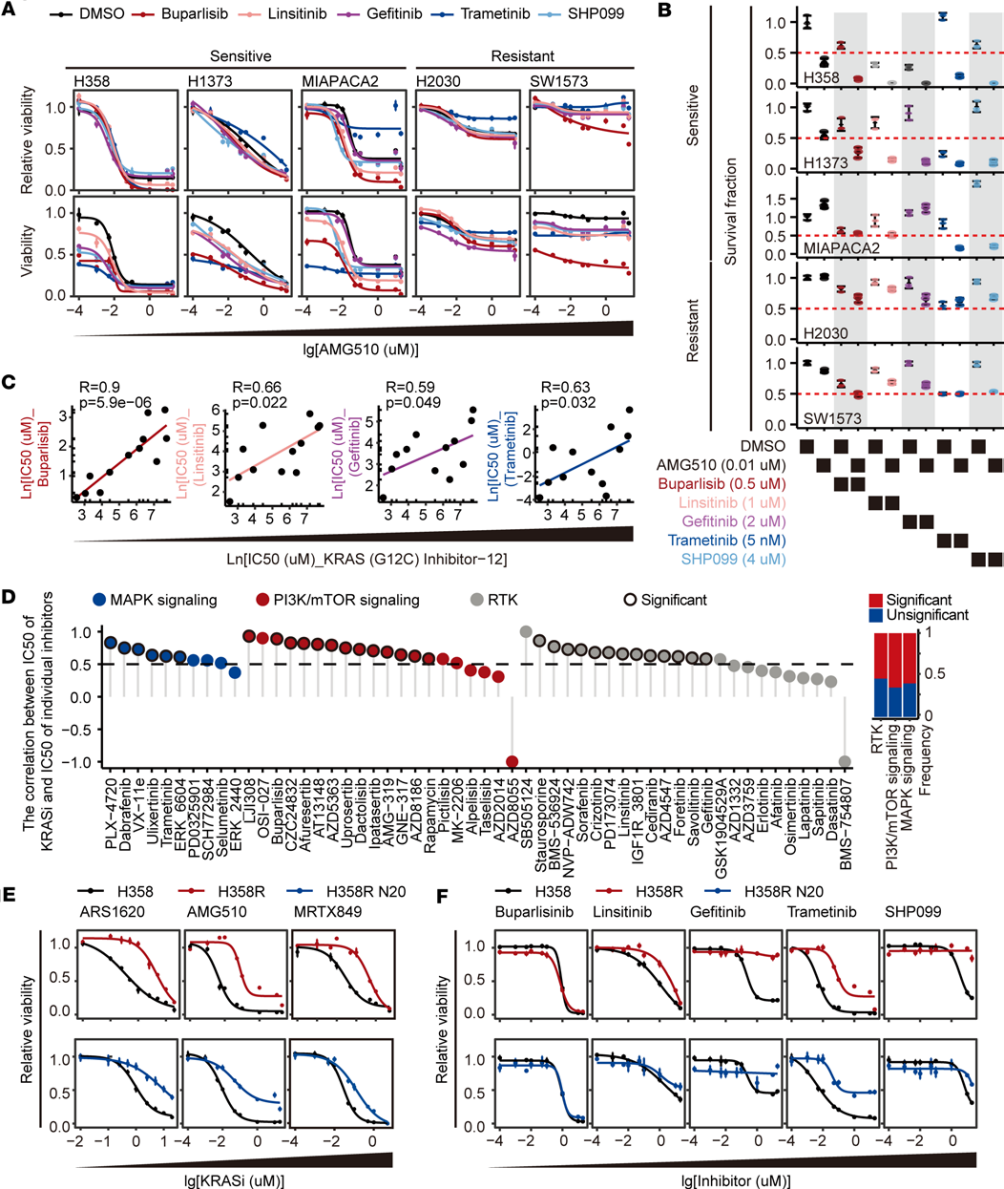
Cancer cells resistant to KRAS G12C inhibitors also display cross-resistance to other inhibitors targeting upstream or downstream components of the RAS signaling pathway. (**A**) Dose-response curves of AMG510 displaying the relative viability (top) and viability (bottom) of H358, H1373, MIAPACA2, H2030, and SW1573 cells following combination treatment with buparlisib (1 μM), linsitinib (0.5 μM), gefitinib (2 μM), trametinib (0.1 μM), or SHP099 (4 μM) for 3 days. (**B**) Dot plots displaying the quantitative results of the clonogenic assay evaluating the growth of H358, H1373, MIAPACA2, H2030, and SW1573 cells upon treatment with buparlisib, linsitinib, gefitinib, trametinib, or SHP099 alone, as well as in combination with AMG510 as in Supplemental Figure 1A. (**C**) Scatter plots depicting the correlation between the IC_50_ values of KRAS (G12C) Inhibitor-12 and the IC_50_ values of buparlisib, linsitinib, gefitinib, or trametinib in 12 KRAS G12C–mutant cell lines. Data were analyzed using Spearman’s rank correlation coefficient. (**D**) Left: Lollipop chart showing Spearman’s correlation coefficient (*R* values) between the IC_50_ values of KRAS (G12C) Inhibitor-12 and the IC_50_ values of 54 individual inhibitors targeting MAPK signaling, PI3K/mTOR signaling, or RTKs in 12 KRAS G12C–mutant cell lines. The dots with a black outline indicate significant correlations. Right: Stacked bar chart presenting the proportion of inhibitors with a significant *R* value in different targeted pathways. (**E** and **F**) Dose-response curves presenting the relative viability of parental H358 cells and acquired-resistance H358R and H358R N20 cells upon treatment with the indicated inhibitors for 5 days. Data are presented as mean ± SEM (**A**, **E**, and **F**) or mean ± SD (**B**).

To tackle KRAS G12C inhibitor resistance, we evaluated the effectiveness of combining AMG510 (sotorasib, KRAS G12C inhibitor) with buparlisib (PI3K inhibitor), linsitinib (IGF1R inhibitor), gefitinib (EGFR inhibitor), trametinib (MEK inhibitor), or SHP099 (SHP2 inhibitor) in multiple KRAS G12C–mutant cancer cell lines, including both sensitive ones (H358, H1373, and MIAPACA2) and resistant ones (H2030 and SW1573). We found that none of these inhibitors enhanced the sensitivity of AMG510 across all 5 cell lines, but they showed a trend of reduced viability when cotreated with AMG510 in sensitive ones and not in resistant ones (Figure 1A). These combination strategies efficiently suppressed cell growth in H358, H1373, and MIAPACA2 cells, as demonstrated by clonogenic assay. However, they failed to do so in resistant H2030 and SW1573 cells (Figure 1B and Supplemental Figure 1A; supplemental material available online with this article; https://doi.org/10.1172/jci.insight.178535DS1).

Upon closer examination of the results, we hypothesized that the synergistic effect of this combination observed in H358, H1373, and MIAPACA2 cells may be attributed to their high sensitivity toward both AMG510 and the other inhibitor, whereas the lack of effectiveness of these strategies observed in H2030 and SW1573 cells may be attributed to their simultaneous resistance to both AMG510 and the other inhibitor (Figure 1, A and B). To address this hypothesis, correlation analyses were performed between the IC_50_ values of KRAS (G12C) Inhibitor-12 and the IC_50_ values of 54 other inhibitors targeting the MAPK signaling pathway, the PI3K/mTOR signaling pathway, or RTKs in 12 KRAS G12C–mutant cell lines in the Genomics of Drug Sensitivity in Cancer (GDSC) database (33). We found that 60% (6 of 10) inhibitors targeting the MAPK signaling pathway, 65% (13 of 20) inhibitors targeting the PI3K/mTOR signaling pathway, and 54.2% (13 of 24) inhibitors targeting RTKs had significant positive correlations with KRAS (G12C) Inhibitor-12, indicating that they share similar sensitivity profiles in these cell lines. Notably, buparlisib, linsitinib, gefitinib, and trametinib were among those inhibitors (Figure 1, C and D, and Supplemental Figure 1B). When tested in cell lines with varying sensitivities to AMG510, buparlisib, linsitinib, gefitinib, trametinib, and SHP099 displayed a trend of greater efficacy in KRAS G12C inhibitor–sensitive cell lines than in resistant cell lines (Supplemental Figure 1C). These data suggest that cell lines with intrinsic resistance to KRAS G12C inhibitors may also display cross-resistance to other inhibitors targeting RTKs, the MAPK signaling pathway, or the PI3K/mTOR signaling pathway. This could result in ineffectiveness when treating these resistant cells with the aforementioned combination strategies.

To expand our observations in acquired-resistance cells, we induced in vitro (H358R) and in vivo (H358R N20) cell models of acquired resistance to KRAS G12C inhibitors. Both H358R cells and H358R N20 cells displayed increased IC_50_ values for ARS1620, AMG510, and MRTX849 (Figure 1E). Importantly, both cell lines exhibited trends toward insensitivity to linsitinib, gefitinib, trametinib, and SHP099 (Figure 1F). Taken together, our study reveals that KRAS G12C–mutant cell lines with either intrinsic or acquired resistance to KRAS G12C inhibitors also display cross-resistance to inhibitors targeting upstream or downstream components of the RAS signaling pathway. The presence of such cross-resistance may weaken the potential synergistic efficacy of these combined treatments.

### The activity of the Hippo-YAP/TAZ pathway is elevated in intrinsically resistant and acquired-resistance cells and is adaptively induced upon treatment with KRAS G12C inhibitors.

**Figure 2:**
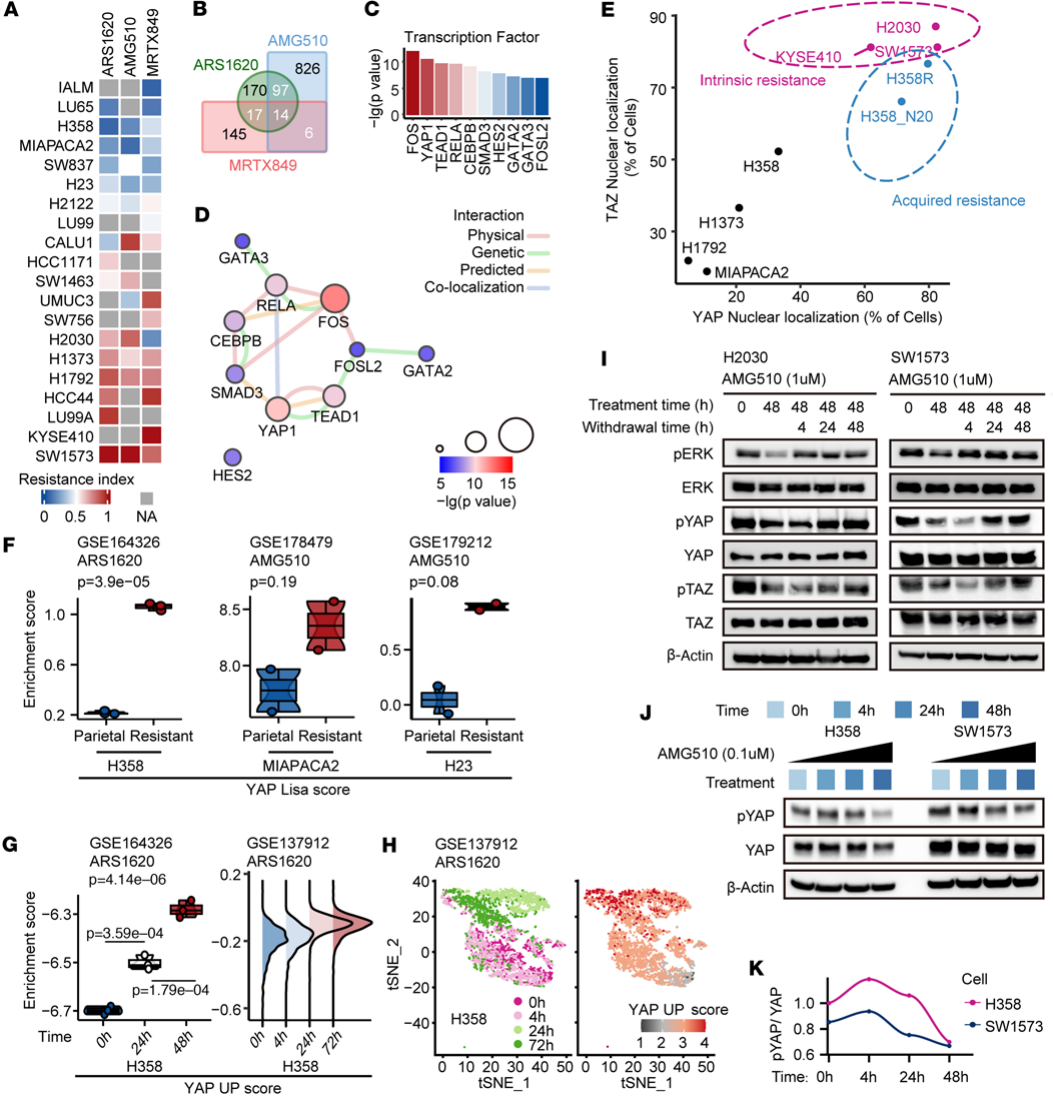
The Hippo-YAP/TAZ pathway is activated in both intrinsic and acquired KRAS G12C inhibitor–resistant cells and is adaptively induced upon KRAS G12C inhibition. (**A**) Heatmap displaying the resistance index of ARS1620, AMG510, and MRTX849 across various KRAS G12C–mutant cancer cell lines. (**B**) Venn diagram illustrating the overlapping relationships among 3 gene sets associated with resistance to KRAS G12C inhibitors as in Supplemental Figure 1D. (**C**) Bar chart presenting the top 10 regulatory factors involved in modulating a set of 134 genes highlighted with white font in **B**. (**D**) Network graph visualizing the interplay between these 10 regulatory factors. (**E**) Quantification of immunofluorescence depicting the subcellular localization of YAP/TAZ in the indicated cells as in Supplemental Figure 2A. (**F**) Box-and-whisker plots (bulk RNA-seq) presenting YAP Lisa scores in parental cells and KRAS G12C inhibitor–resistant cells. (**G**) Box-and-whisker plot (left, bulk RNA-seq) and ridge plot (right, scRNA-seq) displaying YAP UP scores in H358 cells treated with ARS1620 for the indicated duration. (**H**) tSNE plots showing single-cell clustering (left) and YAP UP scores (right) in H358 cells treated with ARS1620 for indicated durations. (**I**) Immunoblots demonstrating changes in YAP/TAZ and ERK phosphorylation levels in intrinsically resistant cells (H2030, SW1573) before and after AMG510 treatment, and following the withdrawal of the drug. (**J** and **K**) Immunoblots (**J**) and line graph (**K**) illustrating the temporal dynamics of YAP phosphorylation in sensitive cells (H358) and resistant cells (SW1573) in response to AMG510 treatment. Data were analyzed using Student’s *t* test (**F**) or 1-way ANOVA followed by Tukey’s test for multiple comparisons (**G**). Blots provided together were set up in parallel at the same time (**I** and **J**).

To address the resistance of KRAS G12C inhibitors, transcriptomic analyses were carried out using the previously published data from 20 KRAS G12C–mutant cell lines (5–7, 34). These cells were ranked according to their sensitivity to KRAS G12C inhibitors, and a module of genes whose expression were higher in resistant cell lines than that in sensitive cell lines was generated for each KRAS G12C inhibitor panel (Figure 2A and Supplemental Figure 1D). From these modules, a gene set containing 134 genes was identified by overlap from at least 2 modules and subjected to transcription factor analysis. Fos proto-oncogene (FOS), YAP1, and TEAD1 were found to be the top 3 significant regulators responsible for the transcription of these genes (Figure 2, B and C). YAP and TEAD drew our attention not only because they interact with each other to regulate transcription, but also because they are tightly connected with other transcription factors identified above (Figure 2D). For instance, a complex consisting of YAP, TAZ, TEADs, and AP-1 (dimer of cFos and transcription factor Jun [JUN] families) was found to synergistically regulate the transcription of target genes (35–37). We, therefore, hypothesized that YAP, TAZ, and TEADs act as key transcription regulators mediating resistance to KRAS G12C inhibitors. Indeed, higher percentages of nucleus-localized YAP/TAZ were observed in intrinsically resistant cells than in sensitive cells (Figure 2E and Supplemental Figure 2, A and B), indicating hyperactivation of YAP/TAZ in cells that are intrinsically resistant to KRAS G12C inhibitors. Supporting this hypothesis, a recent study by Tsai et al. reported an upregulation of YAP1 activity in tumor tissues from patients who developed resistance to KRAS inhibitors (10).

Previous studies have demonstrated that activated Yap, through genetic amplification of *YAP*- or WNT5a-mediated dysregulation of the Hippo pathway, enables bypass of Kras suppression in Kras-driven murine pancreatic cancer and lung cancer models (37–39). We also observed that YAP and TAZ predominantly accumulated in the nucleus of H358R and H358R N20 cells (Figure 2E and Supplemental Figure 2, A and C). Consistently, the YAP Lisa signature (39 genes derived from the aforementioned 134 genes, as described in the Supplemental Methods) was much higher in acquired-resistance cell lines than that in their corresponding parental H358, MIAPACA2, and H23 cell lines, based on the transcriptomic analyses of acquired-resistance cell lines that were generated by previous studies (Figure 2F) (40, 41). Together, these data strongly suggest that YAP/TAZ is activated in cells that are either intrinsically resistant or have acquired resistance to KRAS G12C inhibitors.

### Activation of YAP/TAZ drives KRAS G12C–mutant cancer cells to acquire resistance to KRAS G12C inhibitors.

We wondered whether YAP/TAZ activation occurs early during the treatment with KRAS G12C inhibitors and contributes to the development of acquired resistance. A previous study demonstrated a set of genes that were rescued by the expression of YAP in the specific context of KRAS suppression, and a YAP UP signature was derived from these data in a more stringent way (37). By assessing the level of the YAP UP signature, we observed that YAP was rapidly activated in H358 cells under the treatment with ARS1620, as shown by both bulk and single-cell RNA sequencing (scRNA-seq) (Figure 2, G and H). This phenomenon was further supported by the rapid dephosphorylation of YAP/TAZ in H2030 and SW1573 cells during treatment with a KRAS G12C inhibitor (Supplemental Figure 2D). Moreover, upon withdrawal of the inhibitor, the phosphorylation of YAP/TAZ rapidly returned to baseline level, demonstrating the reversible nature of this effect (Figure 2I). Additionally, the phosphorylation status of YAP/TAZ in acquired-resistance cells swiftly readjusted upon drug withdrawal (Supplemental Figure 2E). Despite both sensitive H358 cells and resistant SW1573 cells showing YAP dephosphorylation and activation upon prolonged KRAS inhibitor treatment, the resistant cells exhibited higher basal YAP activity and responded more rapidly to the KRAS inhibitor to further activated YAP than did the sensitive cells (Figure 2, J and K). Taken together, these data suggest that YAP/TAZ activation is an early event when KRAS G12C–mutant cancer cells are exposed to KRAS G12C inhibitors and this activation is dynamically reversible in resistant cells. YAP activity is inherently higher in intrinsically resistant cells than that in sensitive cells. Over time during KRAS inhibitor treatment, both sensitive and intrinsically resistant cells display elevated YAP activity, ultimately leading to greater resistance.

**Figure 3:**
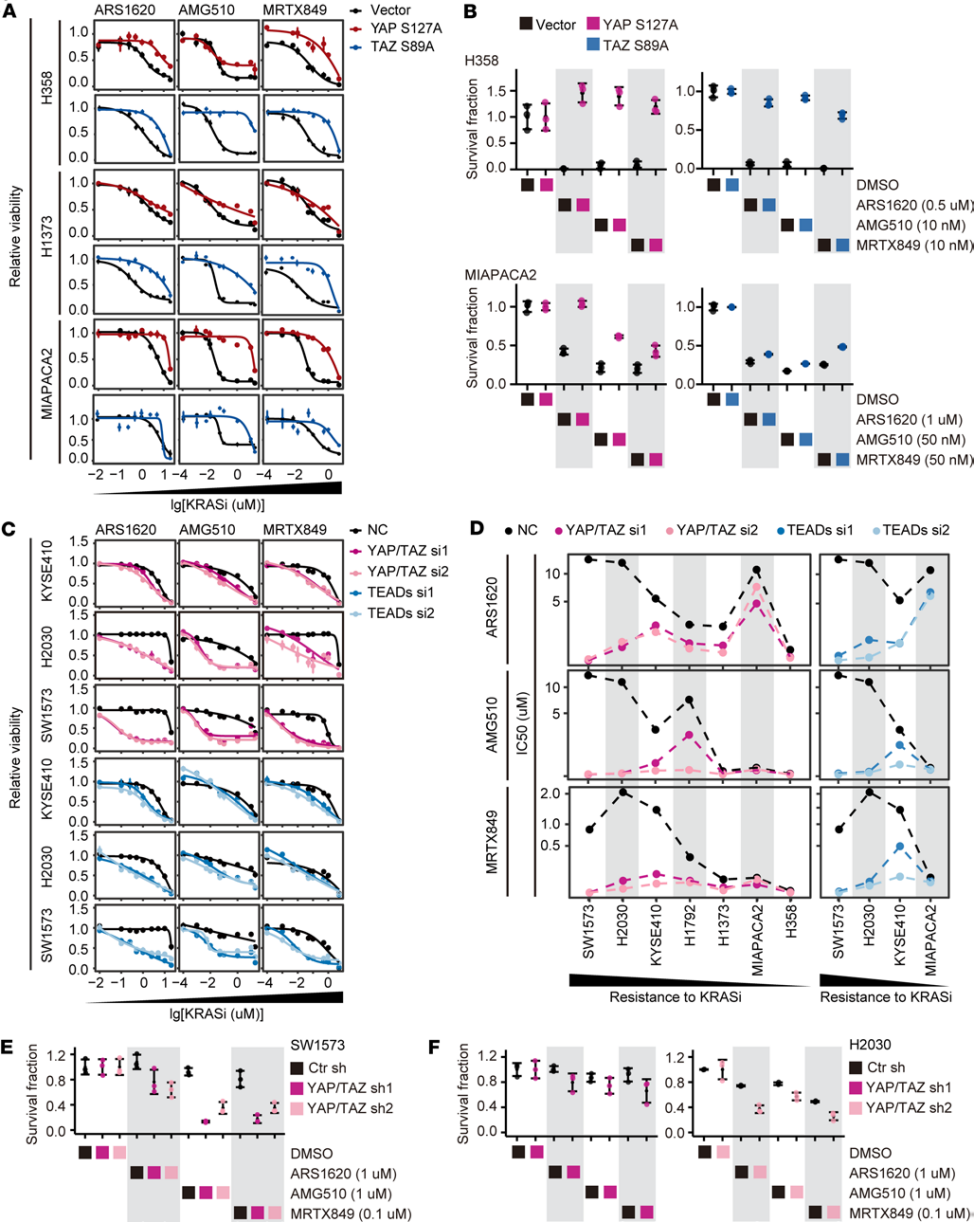
YAP and TAZ are capable of inducing resistance to KRAS G12C inhibitors and are crucial for maintaining this resistance. (**A**) Dose-response curves depicting the relative viability of H358, H1373, and MIAPACA2 cells with or without ectopic expression of constantly activated YAP or TAZ under treatment with ARS1620, AMG510, or MRTX849 for 5 days. (**B**) Dot plots displaying the quantification of clonogenic assay showing the growth of H358 and MIAPACA2 cells with or without ectopic expression of constantly activated YAP or TAZ upon treatment with KRAS G12C inhibitors as in Supplemental Figure 4A. (**C**) Dose-response curves presenting the relative viability of KYSE410, H2030, and SW1573 cells with or without knockdown of YAP/TAZ or TEADs under treatment with ARS1620, AMG510, or MRTX849 for 5 days. (**D**) Dot plots depicting the IC_50_ values (median of at least triplicate experiments) of different cell lines with or without knockdown of YAP/TAZ or TEADs under treatment with the indicated KRAS G12C inhibitors for 5 days. (**E** and **F**) Dot plots displaying the quantification of clonogenic assay showing the growth of SW1573 and H2030 cells with or without knockdown of YAP/TAZ upon treatment with KRAS G12C inhibitors as in Supplemental Figure 4D. Data are presented as mean ± SEM (**A** and **C**) or mean ± SD (**B**, **E**, and **F**).

Along this line, ectopic expression of the constitutively activated forms of YAP (S127A mutant) or TAZ (S89A mutant) in H358, H1373, and MIAPAKA2 cells resulted in their acquired resistance to KRAS G12C inhibitors (Figure 3, A and B, Supplemental Figure 3, A and B, and Supplemental Figure 4A). Given the high conservation between YAP and TAZ in their amino acid sequences, domain structures, and regulatory pathways, we observed a comparable resistance profile for the YAP S127A mutant and the TAZ S89A mutant across various cell lines and KRAS inhibitors (Figure 3, A and B). In addition, ectopic expression of YAP S127A or TAZ S89A in these sensitive cells conferred resistance to inhibitors targeting MEK, IGF1R, EGFR, or PI3K, although the extent of resistance varied among different cell lines or between ectopic expression of YAP and TAZ (Supplemental Figure 5, A and B). In summary, our findings reveal that YAP/TAZ activation occurs upon exposure of sensitive cells to KRAS G12C inhibitors, and their sustained activation eventually results in acquired cross-resistance to KRAS G12C inhibitors and other inhibitors targeting upstream or downstream of the RAS signaling pathway.

### Inhibition of YAP/TAZ strikingly increases the sensitivity of KRAS G12C–mutant cancer cells to KRAS G12C inhibitors.

To explore the role of YAP/TAZ in regulating the sensitivity of KRAS G12C–mutant cancer cells to KRAS G12C inhibitors, we knocked down YAP or TAZ separately in SW1573, a lung cancer cell line that is highly resistant to KRAS G12C inhibitors. We observed that the knockdown of either YAP or TAZ did not notably potentiate SW1573 to 3 KRAS G12C inhibitors, i.e., ARS1620, AMG510, and MRTX849 (Supplemental Figure 4B). YAP and TAZ are homologs and are similarly regulated by the Hippo pathway. Thus, we simultaneously knocked down both YAP and TAZ in multiple KRAS G12C–mutant cell lines, including SW1573, H2030, KYSE410, H1792, H1373, MIAPACA2, and H358 (Supplemental Figure 3C), and we found that this strongly enhanced the sensitivity of these cells to ARS1620, AMG510, and MRTX849 (Figure 3, C and D). Similar observations were replicated in acquired-resistance H358R and H358 N20 cells (Supplemental Figure 4C). Of note, the more resistant the cell is, the more substantial is the decrease in its IC_50_ value for a KRAS G12C inhibitor. Knockdown of YAP/TAZ potentiated all the tested cell lines to a similarly high sensitivity, irrespective of their initial sensitive or resistant status (Figure 3, C and D).

As YAP and TAZ are transcription coactivators of TEADs, we tested whether the knockdown of TEADs had a similar effect. We found that knockdown of all TEAD1–TEAD4 homologs, similar to the strategy of co-knockdown of YAP and TAZ, strongly enhanced the sensitivity of SW1573, H2030, KYSE410, MIAPACA2, H358R, and H358R N20 cells to the 3 KRAS G12C inhibitors (Figure 3, C and D, Supplemental Figure 3D, and Supplemental Figure 4, B and C).

We also confirmed the critical role of YAP/TAZ in sustaining the resistance of KRAS G12C inhibitors in H2030 and SW1573 cells through a clonogenic assay (Figure 3, E and F, Supplemental Figure 3E, and Supplemental Figure 4D). Since LATS1/2 kinases inhibit YAP/TAZ, we therefore ectopically expressed LATS1 in SW1573 cells and found that this enhanced the efficiency of the 3 KRAS G12C inhibitors (Supplemental Figure 4, E–G). Taken together, the results show that the Hippo-YAP/TAZ pathway plays a critical role in sustaining resistance to KRAS G12C inhibitors. Modulating this pathway by inhibiting the downstream transcription core or activating the upstream kinase activity may hold great promise in overcoming the resistance to KRAS G12C inhibitors.

### YAP/TAZ protects cells from KRAS G12C inhibitor–induced apoptosis by downregulating the expression of proapoptotic genes such as BMF, BCL2L11, and PUMA.

**Figure 4:**
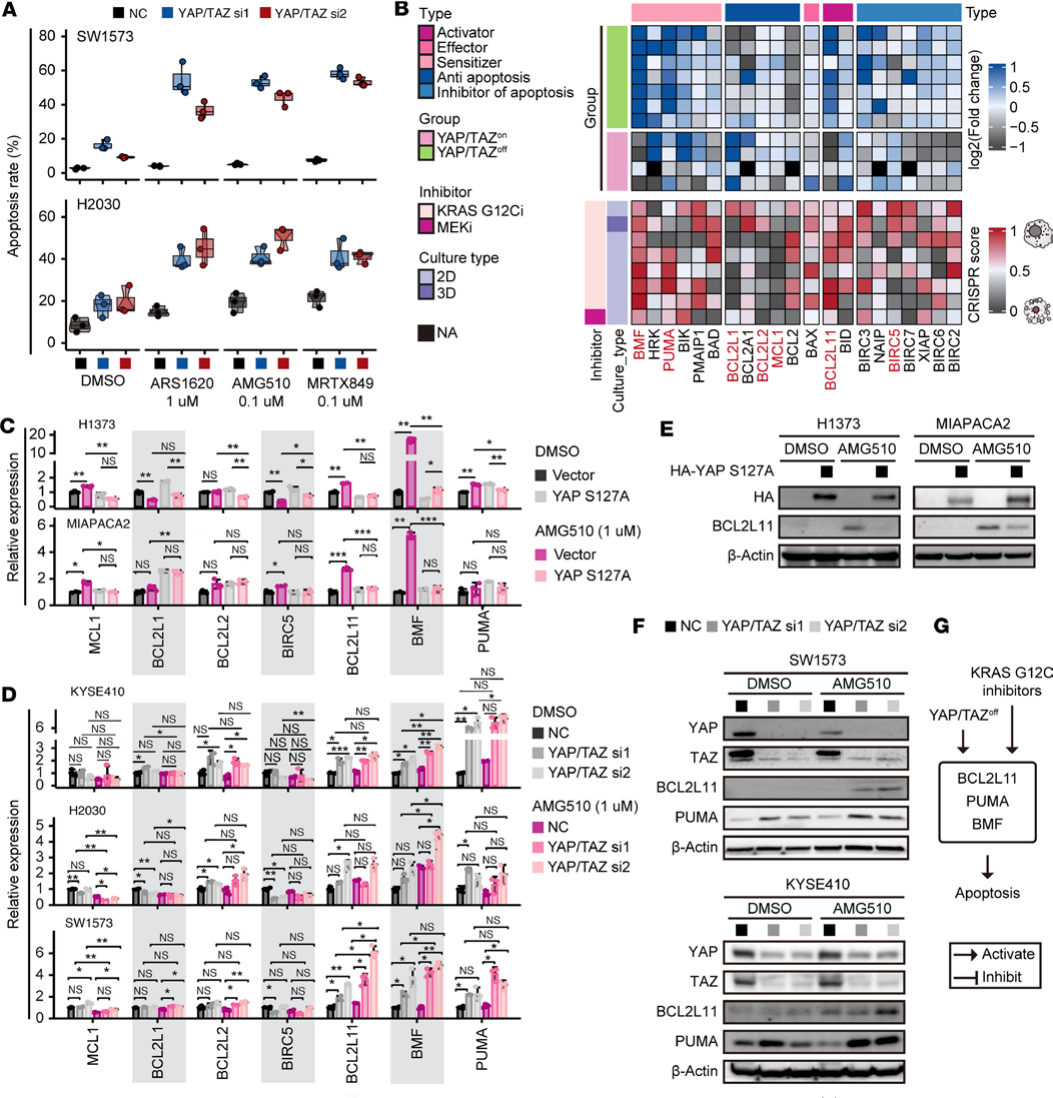
YAP/TAZ prevents KRAS G12C inhibitor–induced cellular apoptosis by downregulating proapoptotic proteins. (**A**) Box-and-whisker plots demonstrating apoptosis rates of SW1573 and H2030 cells with or without knockdown of YAP/TAZ after treatment with the indicated KRAS G12C inhibitors for 5 days. (**B**) Heatmap illustrating the regulation of apoptosis-related genes by YAP/TAZ and their dependency for cell survival upon treatment with inhibitors targeting KRAS G12C or MEK. (**C** and **E**) RT-qPCR (**C**) and immunoblots (**E**) presenting the expression of apoptosis-related genes in H1373 and MIAPACA2 cells with or without ectopic expression of YAP S127A upon 3 days of treatment with AMG510. (**D** and **F**) RT-qPCR (**D**) and immunoblots (**F**) presenting the expression of apoptosis-related genes in KYSE410, H2030, and SW1573 cells with or without knockdown of YAP/TAZ after 1 day of treatment with AMG510. (**G**) Model illustrating that inhibition of YAP/TAZ enhances KRAS G12C inhibitor–induced apoptosis by upregulating the expression of apoptotic proteins. Data in **C** and **D** are presented as mean ± SD and were analyzed using Student’s *t* test, with *P* values adjusted using the false discovery rate method. *P < 0.05; **P < 0.01; ***P < 0.001. NS, not significant. Blots provided together were set up in parallel at the same time (**E** and **F**).

The significant improvement in the efficacy of KRAS G12C inhibitors upon knockdown of YAP/TAZ or TEADs may be attributed to the promotion of cell death or the inhibition of cell proliferation. To determine the underlying mechanism, we evaluated the relationship between YAP/TAZ and cellular apoptosis rates. In resistant SW1573 and H2030 cells, treatment with KRAS G12C inhibitors resulted in a slight increase in apoptosis rates. However, when the knockdown of YAP/TAZ was combined with the treatment of these inhibitors, the apoptosis rates were strikingly upregulated (Figure 4A). To uncover the mechanism by which YAP/TAZ regulates apoptosis specifically in the context of KRAS G12C inhibitor treatment, we conducted bioinformatic analyses of apoptosis-related genes based on previously published RNA-seq and CRISPR screening data (9, 13, 42–48). Our selection criteria for potential target genes were 2-fold; first, the expression of certain genes is reversely regulated under YAP/TAZ activation and inhibition in multiple cell lines, and second, knockout of certain genes consistently leads to a “sensitive phenotype” or a “resistant phenotype” under treatment with KRAS G12C inhibitors or MEK inhibitor across multiple cell lines (Figure 4B). To validate our findings, we employed quantitative real-time PCR (RT-qPCR) to determine the expression of antiapoptotic genes (Bcl-2-like protein 1 [*BCL2L1*], Bcl-2-like protein 2 [*BCL2L2*], Bcl-2-like protein 3 [*MCL1*], and baculoviral IAP repeat–containing protein 5 [*BIRC5*]) and proapoptotic genes (*BMF*, *PUMA*, and *BCL2L11*). We found that only the expression of BCL2L11 and BMF was significantly elevated upon AMG510 treatment in 2 sensitive cell lines, H1373 and MIAPACA2, and this effect was abolished by ectopic expression of the YAP S127A mutant (Figure 4C). Conversely, intrinsically resistant cell lines KYSE410, H2030, and SW1573 did not exhibit induction of BCL2L11, BMF, and PUMA after AMG510 exposure. However, the knockdown of YAP/TAZ prominently boosted the expression of these genes (Figure 4D). Thus, the remarkable enhancement in the potency of KRAS G12C inhibitors upon knockdown of YAP/TAZ could potentially be attributed to the upregulation of these proapoptotic genes. Our observation was further supported by immunoblots (Figure 4, E and F). Taken together, our data suggest that inhibition of YAP/TAZ synergizes with KRAS G12C inhibitors to induce robust apoptosis in cancer cells via upregulating the expression of proapoptotic genes such as *BCL2L11*, *BMF*, and *PUMA* (Figure 4G). To test this hypothesis, we treated SW1573 and H2030 cells with Q-VD-OPh, a pan-caspase inhibitor, or siRNAs targeting the proapoptotic genes *BCL2L11*, *BMF*, and *PUMA*, alongside YAP/TAZ knockdown and KRAS G12C inhibitor treatment. We found that Q-VD-OPh and the siRNAs substantially reduced the apoptosis induced by the combination of YAP/TAZ knockdown and KRAS inhibitor treatment (Supplemental Figure 6, A and B).

### YAP/TAZ reverses KRAS G12C inhibitor–induced proliferation retardation by activating the SLC7A5/mTORC1 axis.

**Figure 5:**
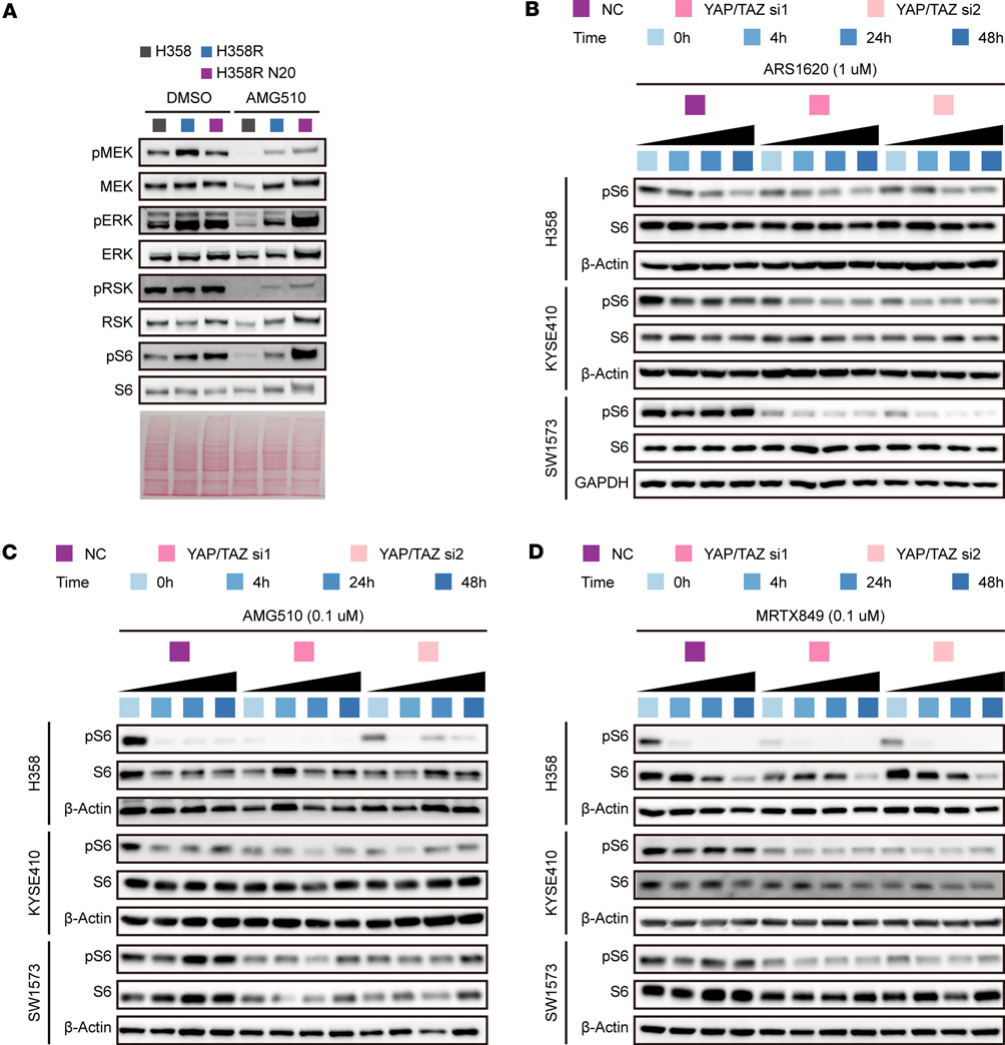
YAP/TAZ mediates the reactivation of mTOR signaling induced by KRAS G12C inhibitors. (**A**) Immunoblots revealing the expression levels and phosphorylation status of MEK, ERK, RSK, and S6 in H358, H358R, and H358R N20 cells after treatment with AMG510 (1 μM) for 3 days. (**B**–**D**) Immunoblots displaying the levels of p-S6 and S6 in H358, KYSE410, and SW1573 cells with or without knockdown of YAP/TAZ under treatment with ARS1620 (**B**), AMG510 (**C**), or MRTX849 (**D**) as indicated. This panel was excerpted from Supplemental Figure 7A. Blots provided together were set up in parallel at the same time (**A**–**D**).

In several intrinsically resistant cell lines, the MAPK and PI3K/mTOR pathways were reactivated within 24–48 hours of treatment with KRAS G12C inhibitors, despite continued suppression of GTP-bound KRAS (11, 12). We observed a similar effect in acquired-resistance cell lines H358R and H358R N20, but not in parental H358 cells (Figure 5A). To investigate whether loss of YAP/TAZ could prevent this reactivation from improving the efficacy of KRAS G12C inhibitors, we analyzed the temporal dynamics of the phosphorylation status of MEK, extracellular signal–regulated kinase (ERK), AKT, and ribosomal protein S6 (S6) across H358, KYSE410, and SW1573 cells during treatment with ARS1620, AMG510, or MRTX849. Although the phosphorylation status of MEK, ERK, and AKT varied under treatment with different KRAS G12C inhibitors or upon the knockdown of YAP/TAZ in multiple cell lines, the phosphorylation of S6 was consistently reduced upon YAP/TAZ knockdown and could not be further reactivated during the exposure to KRAS G12C inhibitors in resistant cell lines KYSE410 and SW1573 (Figure 5, B–D, and Supplemental Figure 7A).

**Figure 6:**
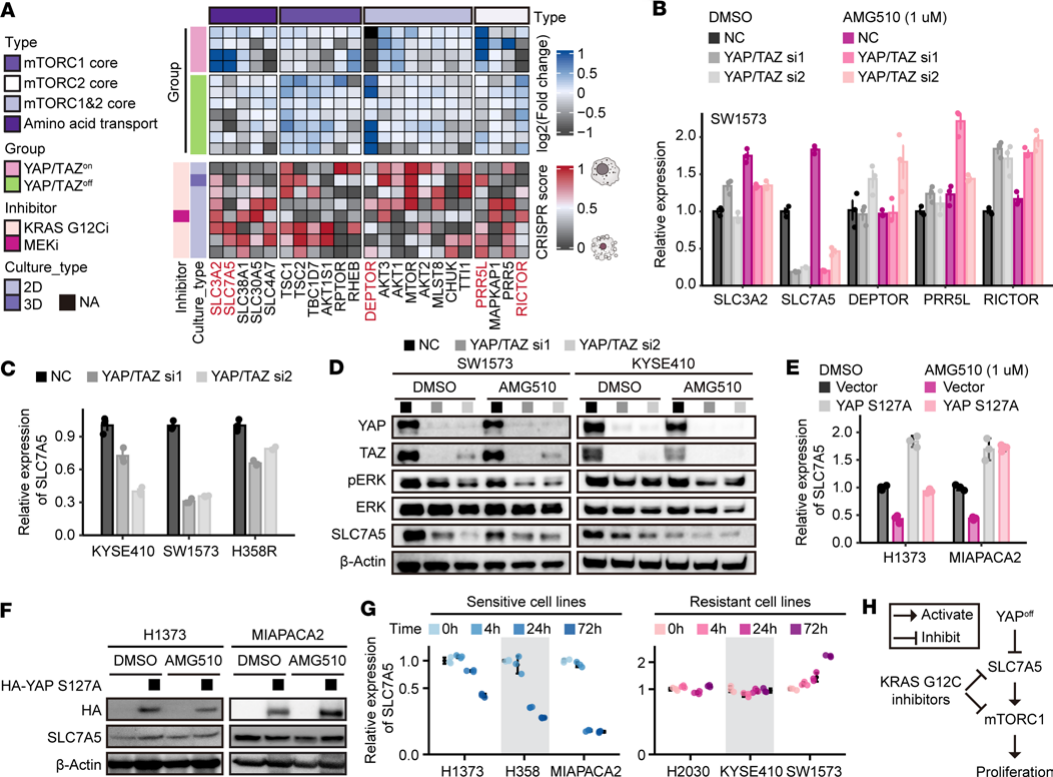
YAP/TAZ promotes the expression of SLC7A5 to overcome proliferation retardation induced by KRAS inhibitors. (**A**) Heatmap illustrating the regulation of mTOR-related genes by YAP/TAZ and their dependency for cell survival upon treatment with inhibitors targeting KRAS G12C or MEK. (**B**) Bar graph presenting the expression of key mTOR-related genes by RT-qPCR in SW1573 cells with or without knockdown of YAP/TAZ upon treatment with AMG510 for 1 day. (**C** and **D**) RT-qPCR (**C**) and immunoblots (**D**) presenting the expression of SLC7A5 and ERK phosphorylation in KYSE410, SW1573, or H358R cells with or without knockdown of YAP/TAZ, in the presence or absence of 1 day of treatment with 1 μM AMG510. (**E** and **F**) RT-qPCR (**E**) and immunoblots (**F**) revealing the expression of SLC7A5 in H1373 and MIAPACA2 cells with or without ectopic expression of YAP S127A after treatment with 1 μM AMG510 for 3 days. (**G**) Dot plots illustrating the expression of SLC7A5 in sensitive and resistant cell lines after different treatment times with 1 μM AMG510. (**H**) Model elucidating that inhibition of YAP/TAZ enhances the proliferation retardation effect of KRAS G12C inhibitors by downregulating the expression of SLC7A5. Data are presented as mean ± SD (**B**, **C**, **E**, and **G**). Blots provided together were set up in parallel at the same time (**D** and **F**).

To investigate how YAP/TAZ regulates the mTOR pathway specifically in the context of KRAS G12C inhibitor treatment, bioinformatic analyses of mTOR-related genes were conducted similarly to those described in Figure 4B (Figure 6A). Proline-rich protein 5-like (*PRR5L*), rapamycin-insensitive companion of mTOR (*RICTOR*), DEP domain–containing mTOR-interacting protein (*DEPTOR*), and *SLC7A5* were selected for further validation in SW1573 cells using RT-qPCR, and only the expression of SLC7A5 was found to be notably reduced upon the knockdown of YAP/TAZ (Figure 6B). Notably, the expression of its partner protein solute carrier family 3 member 2 (SLC3A2), which forms a heterodimer with SLC7A5 to facilitate leucine transport and mTORC1 activation (49), remained unaffected (Figure 6B). These results were confirmed in both intrinsically resistant cell lines, KYSE410 and SW1573, as well as acquired-resistance H358R cells (Figure 6, C and D). Additionally, AMG510 treatment notably reduced the expression of SLC7A5 in sensitive cell lines H1373 and MIAPACA2, but this effect was attenuated or even abolished by the ectopically expressed YAP S127A mutant (Figure 6, E and F).

SLC7A5 is a transporter that primarily facilitates the uptake of extracellular amino acids, particularly leucine, thereby triggering mTOR activation. We observed that exogenous leucine supplementation accelerated S6 reactivation following KRAS inhibition. However, SLC7A5 knockdown prevented leucine from inducing S6 reactivation in both intrinsically resistant and acquired-resistance cells (Supplemental Figure 8A). Additionally, while knockdown of YAP/TAZ attenuated the reactivation of S6 phosphorylation following KRAS inhibitor treatment, ectopically expressed SLC7A5 restored S6 reactivation even in the presence of YAP/TAZ knockdown in SW1573 cells (Supplemental Figure 8, B and C). These findings suggest that SLC7A5 is a critical downstream mediator of YAP/TAZ in mTOR reactivation upon KRAS inhibitor treatment in resistant cells.

Interestingly, while the reduction in SLC7A5 upon AMG510 treatment was consistently observed in sensitive cell lines H1373, H358, and MIAPACA2 in a time-dependent manner, no change in resistant cells H2030 and KYSE410 or even an increase in SW1573 cells was found during AMG510 treatment (Figure 6, B, E, and G). These findings suggest that a reduction in SLC7A5 upon treatment with a KRAS G12C inhibitor could be a valuable indicator of the sensitivity to the treatment for individual cell lines or patients with KRAS G12C mutation.

Given that mTOR is a critical regulator of cell growth and proliferation, we observed that knockdown of YAP/TAZ enhanced AMG510-induced proliferation retardation in resistant cell lines KYSE410, H2030, and SW1573, as indicated by EdU incorporation assay (Supplemental Figure 8D). Notably, ectopic expression of SLC7A5 reversed this growth retardation induced by the combination of KRAS inhibitor treatment and YAP/TAZ knockdown, supporting the role of the YAP/TAZ/SLC7A5 axis in cell proliferation under KRAS inhibitor treatment (Supplemental Figure 8, B and E). Moreover, clonogenic assays revealed that exogenous expression of SLC7A5 conferred resistance to a KRAS inhibitor, while JPH203, an SLC7A5 inhibitor, acted synergistically with a KRAS inhibitor in both intrinsically resistant and acquired-resistance cells (Supplemental Figure 8, B, F, and G).

In conclusion, YAP/TAZ protects cells from KRAS G12C inhibitor–induced apoptosis by downregulating the expression of proapoptotic genes such as *BMF*, *BCL2L11*, and *PUMA*, and they also reverse KRAS G12C inhibitor–induced proliferation retardation by activating the SLC7A5/mTORC1 axis (Figure 4G and Figure 6H). While YAP/TAZ knockdown combined with KRAS inhibition has a more pronounced effect on cellular apoptosis than on proliferation, both contribute to resistance against KRAS inhibitors.

### The combination strategies of KRAS G12C inhibitors and YAP/TAZ inhibition exert synergistic effects in multiple KRAS G12C–mutant cancer cell lines that exhibit intrinsic or acquired resistance to KRAS G12C inhibitors.

**Figure 7:**
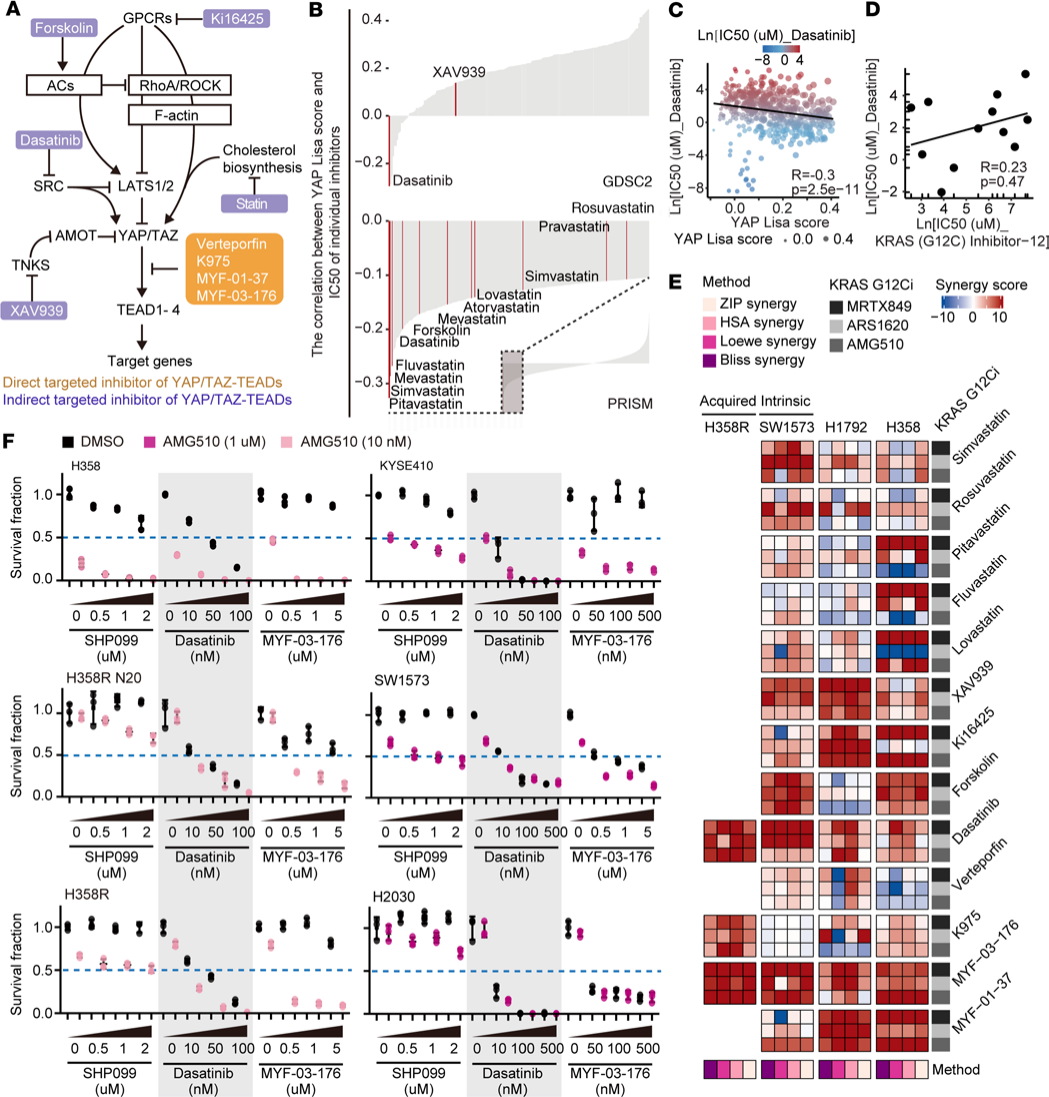
Targeting YAP/TAZ enhances the effectiveness of KRAS G12C inhibition in vitro. (**A**) Scheme illustrating the regulation of the Hippo-YAP/TAZ pathway and potential strategies for targeting YAP/TAZ. (**B**) Bar charts illustrating the correlation between the YAP Lisa scores and the IC_50_ values of individual inhibitors in various cells based on the GDSC2 and PRISM Repurposing datasets. Inhibitors with potential targeting of YAP/TAZ are highlighted in red. (**C**) Scatter plot showing the correlation between the YAP Lisa scores and the IC_50_ values of dasatinib in various cell lines based on the GDSC2 dataset. (**D**) Scatter plot showing the correlation between the IC_50_ values of dasatinib and the IC_50_ values of KRAS (G12C) Inhibitor-12 in 12 KRAS G12C–mutant cell lines. (**E**) Heatmap displaying the synergy scores of combination strategies of KRAS G12C inhibitors and individual inhibitors targeting YAP/TAZ across various cell lines. (**F**) Quantitative results of clonogenic assay displaying the combined effects of AMG510 with SHP099, dasatinib, or MYF-03-176 in H358, H358R N20, H358R, KYSE410, SW1573, and H2030 cells as in Supplemental Figure 9D. Data are presented as mean ± SD (**F**).

As the knockdown of YAP/TAZ has been shown to strikingly enhance the sensitivity to ARS1620, AMG510, and MRTX849 in almost all tested KRAS G12C–mutant cell lines (Figure 3, C and D, and Supplemental Figure 4C), we sought to explore the potential of small molecular compounds to achieve a similar effect. A panel of compounds that indirectly modulate the Hippo pathway or directly modulate the formation of a YAP-TAZ-TEAD complex were selected for testing (Figure 7A) (21). Interestingly, we observed that the IC_50_ values of dasatinib, statins, and forskolin exhibited a reverse correlation with the YAP Lisa scores in either GDSC2 or PRISM Repurposing datasets (Figure 7B and see Methods). Approximately half of the drugs targeting YAP/TAZ displayed a negative correlation with YAP Lisa scores (Supplemental Figure 9A). For instance, cells with higher YAP activity exhibited a better response to dasatinib (Figure 7, B and C). However, drugs targeting upstream or downstream components of the RAS signaling pathway tended to exhibit no significant correlation or positive correlation with YAP Lisa scores (Supplemental Figure 9A).

Importantly, in KRAS G12C–mutant cell lines, the IC_50_ values of KRAS (G12C) Inhibitor-12 did not display a significant positive correlation with the IC_50_ values of dasatinib (Figure 7D). In contrast, it was remarkably positively correlated with the IC_50_ values of various inhibitors of RTKs, PI3K/mTOR signaling, or MAPK signaling (Figure 1, C and D, and Supplemental Figure 1B). These data imply that KRAS G12C–mutant cancer cells, which are resistant to KRAS G12C inhibitors and exhibit elevated YAP/TAZ activity, might display sensitivity to YAP/TAZ inhibitors such as dasatinib.

Two rounds of screening were carried out to determine the synergistic effect between these compounds and KRAS G12C inhibitors with a checkerboard assay and clonogenic assay, respectively (Figure 7, E and F, and Supplemental Figure 9, B–D). We found that dasatinib and MYF-03-176 exhibited extraordinarily synergistic effects with KRAS G12C inhibitors in multiple cell lines (Figure 7, E and F, and Supplemental Figure 9, B–D). Dasatinib, a clinically used anticancer agent, can inhibit YAP/TAZ by suppressing SRC kinases (50, 51). MYF-03-176 can directly disrupt the YAP-TAZ-TEAD complex by covalent binding with the palmitoylation pocket of TEADs (Figure 7A). Notably, these effects were much better than that of SHP099, an SHP2 inhibitor that has gained attention in current clinical trials for its potential to overcome resistance to KRAS G12C inhibitors (Figure 7F and Supplemental Figure 9D).

While previous studies have shown that SRC kinases can directly activate YAP by phosphorylating YAP at Y341/Y357/Y394, and indirectly activate YAP by reducing YAP S127 phosphorylation through inhibiting LATS1/2 kinases, SRC kinases also target other proteins such as caveolin-1, STAT3, and p130Cas (50, 52). Although we observed that dasatinib notably inhibits YAP/TAZ activity via promoting their phosphorylation and reducing the transcription of their canonical target genes, *CTGF* and *CYR61* (Supplemental Figure 10, A and B), it remains uncertain whether dasatinib’s efficacy in combination with KRAS inhibitors relies solely on the effective inhibition of YAP/TAZ activity. To investigate this, the YAP S127A and the YAP S127A/3YE (Y341E/Y357E/Y394E) mutants were introduced into cells sensitive to KRAS G12C inhibitors. Clonogenic assays revealed that both mutants promoted cells to acquire resistance to dasatinib as well as AMG510. More importantly, both mutants diminished the synergistic effect between dasatinib and KRAS G12C inhibitor, with the YAP S127A/3YE mutant being more potent than the YAP S127A mutant (Supplemental Figure 10, C–G). Together, our data suggest that dasatinib’s efficacy in combination with KRAS inhibitors depends on effective inhibition of YAP/TAZ activity. Both the direct dasatinib/SRC/YAP axis and the indirect dasatinib/SRC/LATS1/2/YAP/TAZ axis are critical for dasatinib’s synergistic effect with KRAS inhibitors.

**Figure 8:**
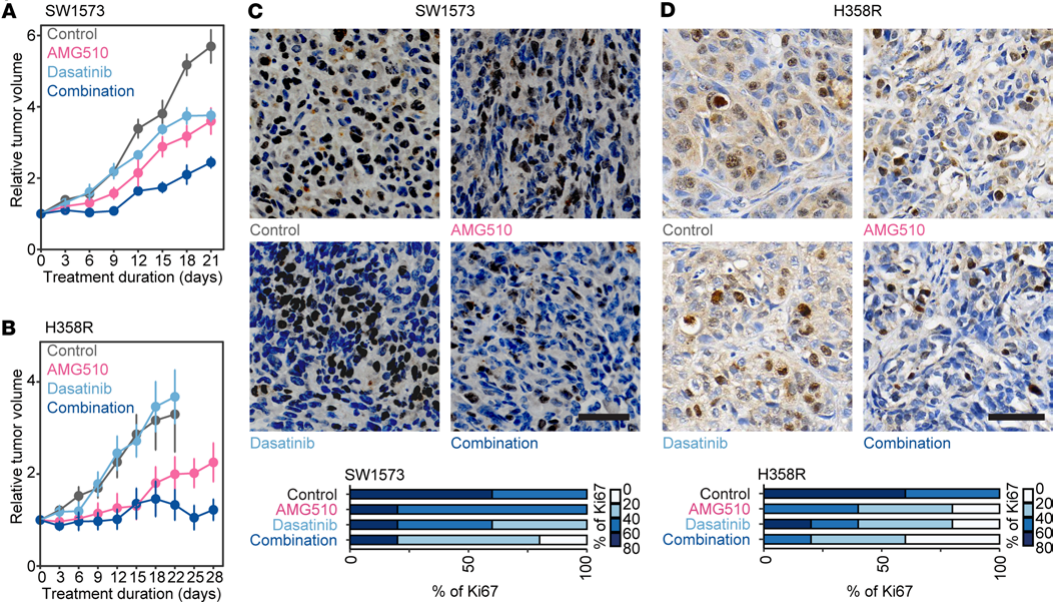
Dasatinib enhances the effectiveness of KRAS G12C inhibition in vivo. (**A** and **B**) Relative tumor volume curves of SW1573 (**A**) and H358R (**B**) xenografts treated with AMG510, dasatinib, or both in combination. *n* = 5 mice per group. (**C** and **D**) Representative images and quantifications of immunohistochemistry of Ki67 in SW1573 and H358R xenografts treated with AMG510, dasatinib, or both in combination. Scale bars: 50 μm (**C**) and 60 μm (**D**). Data are presented as mean ± SEM (**A** and **B**).

The combination with dasatinib and AMG510 resulted in a notable reduction in tumor volumes in xenografts of SW1573, which is the most intrinsically resistant cell line detected yet (Figure 8A). In the acquired-resistance cell line H358R, this combination strategy almost prevented the progression of xenograft tumors, substantially surpassing the individual efficacy of dasatinib or AMG510 alone (Figure 8B). The combination strategy led to a substantial reduction in cellular proliferation, as demonstrated by the diminished proportion of Ki67-positive cancer cells (Figure 8, C and D), and a significant increase in cleaved caspase-3–positive cells, a pivotal marker of apoptosis (Supplemental Figure 11, A and B). These observations indicate that the combination therapy effectively impedes tumor progression through simultaneously suppressing proliferation and enhancing apoptotic cell death. Importantly, this combination strategy had minimal impact on body weight, indicating its potential tolerability (Supplemental Figure 11, C and D). In addition, we evaluated the impact of simvastatin and MYF-03-176 on potentiating the effectiveness of AMG510 in SW1573 cells. Although they were not as effective as dasatinib, both compounds substantially reduced the tumor volumes when in combination with AMG510, without affecting body weight (Supplemental Figure 11, E and F). Taken together, our data show that inhibitors that directly or indirectly modulate YAP/TAZ/TEADs represent a practical approach to overcome intrinsic and acquired resistance to KRAS G12C inhibitors.

### Inhibition of YAP/TAZ strikingly enhances the sensitivity of KRAS G12D–mutant cancer cell lines to KRAS G12D inhibitor.

**Figure 9:**
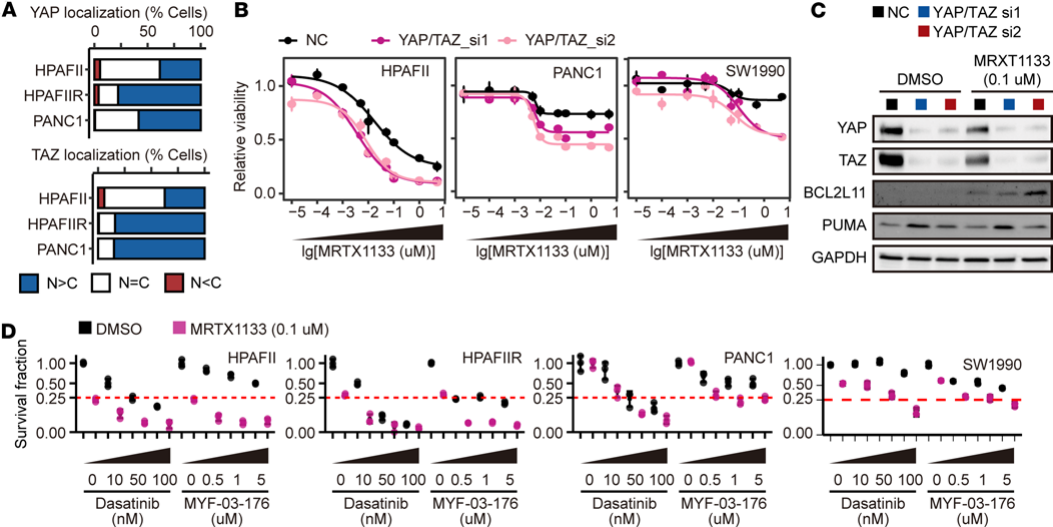
The Hippo-YAP/TAZ pathway is activated in both intrinsic and acquired KRAS G12D inhibitor–resistant cells and is crucial for maintaining this resistance. (**A**) Quantifications of immunofluorescence depicting the subcellular localization of YAP/TAZ in KRAS G12D–mutant cell lines as in Supplemental Figure 12A. N, nuclear; C, cytosolic. (**B**) Dose-response curves depicting the relative viability of HPAFII, PANC1, and SW1990 cells with or without knockdown of YAP/TAZ under treatment with MRTX1133 for 5 days. NC, negative control. (**C**) Immunoblots revealing the expression levels of BCL2L11 and PUMA in PANC1 cells with or without knockdown of YAP/TAZ upon treatment with MRTX1133 for 3 days. (**D**) Quantification of clonogenic assay displaying the combined effects of MRTX1133 with dasatinib or MYF-03-176 in HPAFII, HPAFIIR, PANC1, and SW1990 cells as in Supplemental Figure 12C. Data are presented as mean ± SEM (**B**) or mean ± SD (**D**). Blots provided together were set up in parallel at the same time (**C**).

Among the various KRAS mutations in human cancers, G12C, G12D, G12V, and G12A are the most prevalent (2). Recently, inhibitors targeting the KRAS G12D mutation have also transitioned from being “undruggable” to becoming “druggable.” Hence, we extended our findings to KRAS G12D–mutant pancreatic cancer cells. We discovered that, compared with the sensitive pancreatic cancer cell line HPAFII, both the intrinsically resistant cell line PANC1 and the acquired-resistance cell line HPAFIIR exhibited a high percentage of nucleus-localized YAP and TAZ, indicating the activation of YAP/TAZ in both intrinsically and acquired KRAS G12D inhibitor–resistant pancreatic cancer cells (Figure 9A and Supplemental Figure 12A). In addition, the knockdown of YAP/TAZ resulted in robust sensitization of KRAS G12D–mutant cells to MRTX1133, a KRAS G12D inhibitor, possibly due to the downregulation of SLC7A5 and the upregulation of proapoptotic genes such as *BCL2L11* and *PUMA* upon MRTX1133 treatment (Figure 9, B and C, Supplemental Figure 12B, and Supplemental Figure 13A). In line with this, the SLC7A5 inhibitor JPH203 exhibited synergistic effects when combined with a KRAS G12D inhibitor in 3 pancreatic cancer cell lines carrying the KRAS G12D mutation. In contrast, SLC7A5 overexpression conferred resistance to the KRAS G12D inhibitor (Supplemental Figure 13, B and C). We further confirmed that dasatinib and MYF-03-176 potentiated the effect of MRTX1133 on HPAFII, HPAFIIR, PANC1, and SW1990 cells (Figure 9D and Supplemental Figure 12C). Collectively, these data suggest that the Hippo-YAP/TAZ pathway plays a critical role in developing and maintaining resistance to both KRAS G12C inhibitors and a KRAS G12D inhibitor. This general resistance mechanism to multiple KRAS inhibitors sheds light on an opportunity to overcome resistance to KRAS inhibitors and improve outcomes for patients with KRAS-mutant cancers through modulation of the Hippo-YAP/TAZ pathway.

## Discussion

**Figure 10:**
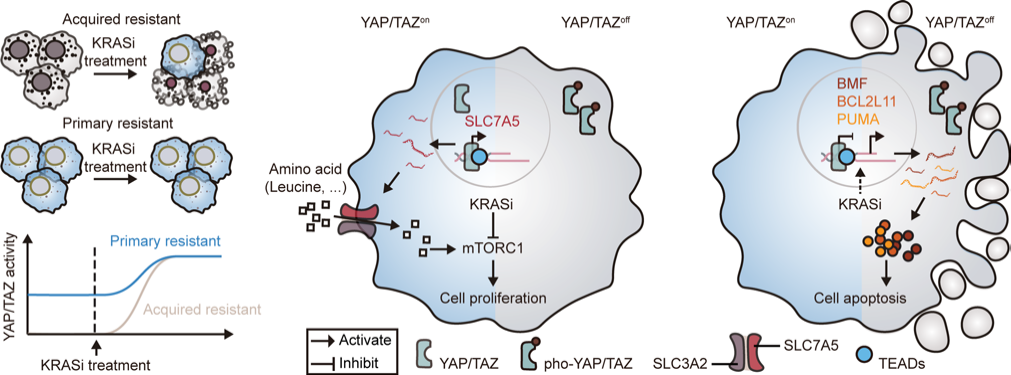
Model illustrating the roles of YAP/TAZ in developing and maintaining resistance to KRAS inhibitors. KRAS inhibitors (KRASi) can induce upregulation of YAP/TAZ activity, which in turn protects cells from KRAS inhibitor–induced apoptosis by downregulating proapoptotic genes such as *BMF*, *BCL2L11*, and *PUMA*, while also reversing proliferation retardation through activation of the SLC7A5/mTORC1 axis.

Various mechanisms have been observed in developing resistance to sotorasib and adagrasib. These include the emergence of additional mutations in the *KRAS* gene, high-level amplification of the *KRAS G12C* allele, and genetic changes in genes (such as neuroblastoma RAS viral oncogene homolog [*NRAS*], v-Raf murine sarcoma viral oncogene homolog B [*BRAF*], v-Raf-1 murine leukemia viral oncogene homolog 1 [*RAF1*], tyrosine-protein kinase Met [*MET*], or phosphatase and tensin homolog [*PTEN*]) within the RAS signaling pathway (8–10). Additionally, bypass signaling pathways like the MAPK or PI3K pathway can become activated without involving mutational events (10, 12). In this regard, our study has identified the activation of the Hippo-YAP/TAZ pathway as a crucial mechanism in developing and sustaining resistance to KRAS inhibitors, where YAP and TAZ downregulate proapoptotic genes to protect cells from KRAS inhibitor–induced apoptosis and activate the SLC7A5/mTORC1 axis to reverse KRAS inhibitor–induced proliferation retardation (Figure 10). These findings provide valuable insights for developing practical therapeutic approaches to combat drug resistance in KRAS-mutant cancers.

While very recent studies have implied the involvement of YAP in sotorasib resistance (32, 53), our study provides substantial advancements on multiple fronts. Firstly, we demonstrate that YAP/TAZ activation occurs in cells with intrinsic or acquired resistance to KRAS G12C inhibitors. Secondly, we illustrate that cells with intrinsic or acquired resistance to KRAS G12C inhibitors confer cross-resistance to drugs targeting the upstream or downstream components of the RAS signaling pathways. Thirdly, we highlight the essential role of YAP and TAZ, rather than solely YAP, in sustaining resistance to sotorasib, adagrasib, and ARS1620 across all 9 tested cell lines. It is important to emphasize that while YAP and TAZ share high homology, they also exhibit structural and functional differences. Their expression patterns and activities are not entirely identical and are regulated by different upstream factors. Although current targeting approaches aim at both of these targets simultaneously, it is worth noting that in the era of RNA delivery, selectively delivering YAP siRNA and simultaneously delivering YAP/TAZ siRNA may yield distinct outcomes in overcoming resistance to KRAS inhibitors. Lastly, and perhaps most importantly, our findings also apply to resistance against KRAS G12D inhibition in pancreatic cancer cells. While it may seem relatively straightforward to extrapolate the findings from KRAS G12C to KRAS G12D, we want to underline that various KRAS mutation variants exhibit significant functional disparities (54). It is crucial to not overlook the differences among different KRAS mutation variants. Furthermore, even within KRAS G12C–mutant tumors, there are notable differences in the mechanisms of resistance to KRAS G12C inhibitors between non–small cell lung cancer and colorectal cancer. EGFR signaling was identified as the dominant mechanism of resistance to KRAS G12C inhibitors in colorectal cancer rather than in non–small cell lung cancer (55). These highlight the importance of avoiding the assumption of universal applicability of resistance mechanisms to KRAS inhibitors across different tumor types and KRAS mutation variants, as it oversimplifies the complexity of the issue. Therefore, it is crucial to engage in rigorous scientific investigations to establish the generalizability of YAP/TAZ-mediated resistance to KRAS inhibition across a wide spectrum of tumor types and distinct KRAS mutation variants.

Combination strategies targeting KRAS G12C and upstream regulators or downstream effectors of the RAS signaling have been extensively explored to overcome resistance to sotorasib or adagrasib (9–12, 56). However, the effectiveness of those combination strategies has shown variations across different cell lines. For instance, the combination of ARS1620 with an anaplastic lymphoma kinase (ALK) inhibitor or FGFR inhibitor has shown effectiveness in H358 and H23 cell lines, but its efficacy is substantially diminished in H1792, SW1463, and SW1573 cell lines (12). Remarkably, our study unexpectedly discovered that KRAS G12C–mutant cancer cells, whether they exhibit intrinsic or acquired resistance to KRAS G12C inhibitors, also display cross-resistance to other inhibitors targeting EGFR, IGF1R, MEK, or SHP2 (Figure 1). Consequently, the combination of these inhibitors with KRAS G12C inhibitors proved ineffective in treating these resistant cells. It is important to note that our conclusion does not disregard the potential for synergistic cytotoxicity with such combination strategies. However, we should be aware that the occurrence of cross-resistance poses a significant obstacle that cannot be overlooked during combination therapy. Among all combination strategies to enhance the antitumor activity of KRAS G12C inhibitors (9–12, 56), the inhibition of SHP2, which connects RTKs to the RAS signaling pathway and is particularly vulnerable in cancer cells with genetic alteration in *KRAS*, has attracted considerable attention (12, 56–59). Ongoing clinical trials (TNO155 by Novartis, ClinicalTrials.gov NCT04330664; RMC-4630 by Revolution Medicine, ClinicalTrials.gov NCT05054725) are investigating the potential of SHP2 inhibitors to overcome resistance to KRAS G12C inhibitors. In the ClinicalTrials.gov cohort NCT04185883, as reported at the International Association for the Study of Lung Cancer 2022 World Conference on Lung Cancer, the combination of sotorasib and RMC-4630 demonstrated a strong synergistic effect in treatment-naive patients harboring the KRAS G12C mutation. However, the efficacy of this combination was less pronounced in the population that had previously received KRAS G12C inhibitors. This outcome aligns with our previous concerns that cancer cells acquiring resistance to KRAS G12C inhibitors may also develop cross-resistance to SHP2 inhibitors as well as other inhibitors targeting upstream or downstream of the RAS signaling pathway. This presents a noteworthy obstacle that must be considered when exploring combination therapy to overcome resistance to KRAS inhibitors.

In this study, we found that knockdown of YAP/TAZ or TEAD1–TEAD4 sensitized KRAS G12C inhibitors in all these cells (Figure 3, C and D, and Supplemental Figure 4C). It has been documented that resistance to sotorasib commonly arises from genetic changes affecting multiple components of the RAS signaling pathway (8–10). Considering that the cell lines tested in our study have diverse genetic backgrounds, the activation of YAP/TAZ may be the underlying cause of this cross-resistance. This hypothesis is supported by the fact that ectopic expression of constantly activated YAP or TAZ mutants contributes to acquired resistance to not only KRAS G12C inhibitors, but also inhibitors targeting MEK, IGF1R, EGFR, or PI3K (Supplemental Figure 5). In fact, accumulating evidence suggests that the dysregulation of the Hippo-YAP/TAZ pathway serves as a central resistance mechanism in multiple therapies, particularly in the intrinsic and acquired resistance to RTK, RAF, or MEK inhibitors (24, 28, 29). In light of the potential involvement of the Hippo-YAP/TAZ pathway in cross-resistance, further exploration of targeting this pathway as a therapeutic approach may hold promise in overcoming resistance against inhibitors targeting the RTK/RAS signaling pathway.

Targeting YAP/TAZ is an appealing strategy to overcome drug resistance in cancers. However, direct inhibition of YAP and TAZ remains challenging due to their conformational flexibility and integration of multiple oncogenic pathways. Alternatively, targeting the YAP/TAZ signaling network through upstream regulators (e.g., the Hippo signaling pathway) or downstream effectors (e.g., TEAD binding) offers a more feasible approach to block aberrant YAP/TAZ activation effectively at present. It is worth mentioning that a recent study has explored the potential of overcoming resistance to sotorasib by an allosteric pan-TEAD inhibitor called GNE-7883 (31). In line with our findings, both MYF-03-176 and GNE-7883 have demonstrated efficacy in overcoming intrinsic and acquired resistance to KRAS inhibitors. However, the transition from preclinical to clinical application of these chemicals requires time. To expedite the clinical application of these findings, we conducted a screening for clinically approved drugs targeting upstream regulators of YAP/TAZ. Compounds like statins and dasatinib have been identified and have shown promising synergistic effects with KRAS inhibitors. In particular, dasatinib, a clinical anticancer drug, has demonstrated superior effects in overcoming resistance to KRAS inhibitors compared with SHP2 inhibitor SHP099. In the acquired KRAS G12C inhibitor–resistant H358R and H358R N20 cells, dasatinib remarkably enhanced the sensitivity of AMG510. In the intrinsically KRAS G12C inhibitor–resistant KYSE410, SW1573, and H2030 cells, dasatinib synergized with AMG510, partially attributable to its high effectiveness in these cells, which exhibit superactivation of YAP/TAZ (Supplemental Figure 9D). Of great significance, our study also highlights that utilizing dasatinib as a combination therapy led to improved treatment outcomes compared with using the KRAS G12D inhibitor alone. Our findings lay the groundwork for future studies and clinical trials to validate the clinical effectiveness and safety of dasatinib in combination approaches, ultimately advancing the development of more effective treatment options for patients with resistance to KRAS inhibitors.

In conclusion, our study highlights that cross-resistance limits the effectiveness of combination strategies in overcoming resistance to KRAS inhibitors. We demonstrate the crucial role of the Hippo-YAP/TAZ pathway in both acquiring and maintaining resistance to KRAS inhibitors via concurrently modulating the expression of proapoptotic proteins and the SLC7A5/mTORC1 axis (Figure 10). Moreover, targeting this pathway, such as by dasatinib, shows potential for overcoming resistance to KRAS inhibitors and improving outcomes for patients with KRAS-mutant cancers.

## Methods

### Sex as a biological variable.

Our study exclusively examined female mice. It is unknown whether the findings are relevant for male mice.

### Chemicals and reagents.

AMG510, MRTX1133, dasatinib, MYF-01-37, MYF-03-176, and K975 were synthesized by DC Chemicals. Simvastatin, rosuvastatin, pitavastatin, fluvastatin, lovastatin, XAV-939, Ki16425, forskolin, ARS1620, gefitinib, linsitinib, trametinib, SHP099, PEG300, and Tween 80 were purchased from Selleckchem. Verteporfin, MRTX849, and buparlisib were supplied by MedChemExpress. DMSO and L-leucine were purchased from Sigma-Aldrich.

### Cell lines and cell culture.

H358, MIAPACA2, H1373, H1792, H2030, SW1573, PANC1, SW1990, HPAFII, LS180, and HEK293T cells were obtained from the American Type Culture Collection. The KYSE410 cell line was obtained from the European Collection of Cell Cultures. H358, H1373, H1792, H2030, and KYSE410 cells were cultured in RPMI 1640 (Procell). MIAPACA2, SW1573, PANC1, SW1990, LS180, and HEK293T cells were cultured in DMEM (Procell). HPAFII was cultured in MEM (Procell). All culture media were supplemented with 10% fetal bovine serum, 100 U/mL penicillin, and 100 μg/mL streptomycin. All cells were cultured in a humidified incubator at 37°C with 5% CO_2_.

### Construction of plasmids and generation of stable cells.

The pLVX-puro vector, pLVX-puro-YAP2 S127A, and pCDN3-TAZ S89A plasmids were obtained from the Bio-research innovation center Suzhou (http://www.brics.ac.cn/). The pLV3-puro-SLC7A5 (P48869) plasmid was obtained from Miaoling Biotech. The pLVX-YAP S127A/3YE plasmid was synthesized by Sangon Biotech based on the sequence provided by Li et al. (52). The plasmid containing LATS1 was stored in our laboratory. TAZ S89A was subcloned into the pLVX-puro vector and the pMCB-puro vector. LATS1 was subcloned into the pCDH-GFP vector. The shRNA sequences targeting YAP or TAZ were designed following the Addgene protocol. The YAP shRNAs were cloned into the PMKO.1-hygro vector, while the TAZ shRNAs were cloned into the PLKO.1-puro vector. The sequences of all shRNAs used in this study are listed in Supplemental Table 1.

To produce viruses, plasmids derived from the pLVX, pCDH, pLV3, or pLKO.1 backbone were cotransfected into HEK293T cells with the packaging plasmids psPAX2 and pMD2.G using a transfection reagent (Polyplus). Similarly, plasmids based on the pMCB3 and pMKO.1 backbone were cotransfected with packaging plasmids GAG and VSVG. After 48 hours, the virus-containing supernatant was collected from HEK293T cells and filtered through a 0.22-μm filter (Millipore). The resulting filtered viral suspension was then used to infect cancer cells (H358, H1373, MIAPACA2, KYSE410, SW1573, and H2030) in a culture medium containing 1 μg/mL polybrene (Solarbio). After 2 days, the cells were subjected to selection using either puromycin (TargetMol) or hygromycin B (TargetMol), or sorted by flow cytometry. The efficiency of knockdown or overexpression was subsequently validated by immunoblotting.

### Generation of resistant cancer cell lines.

To establish KRAS G12C inhibitor–resistant cell lines, H358 cells were cultured in a medium containing gradually increasing concentrations (0.1 μM to 1 μM) of AMG510, with regular medium changes every 2–3 days. After approximately 6 months of induction in vitro, the KRAS G12C inhibitor–resistant cell line, H358R, was generated. Furthermore, a xenograft resistance model was generated by orally administering mice bearing H358 xenograft tumors with 30 mg/kg AMG510 daily until the tumors grew in the presence of the inhibitor; after this, tumors were dissociated into single-cell suspensions using a tumor dissociation kit (Miltenyi Biotec) and the KRAS G12C inhibitor–resistant cell line, H358R N20, was obtained by continuous passaging to eliminate mouse cells. The above 2 resistant cell lines were maintained in the presence of 1 μM AMG510. Similarly, to generate a KRAS G12D inhibitor–resistant cell line, HPAFII cells were cultured with gradually increasing concentrations of MRTX1133 (0.01 μM to 0.1 μM), resulting in the in vitro resistant cell line after approximately 4 months of induction. The HPAFIIR cells were maintained in the presence of 0.1 μM MRTX1133.

### siRNA transfection.

siRNA transfection was performed according to the manufacturer’s protocols (Polyplus). The knockdown efficiency was evaluated 48 hours after transfection using immunoblotting. The sequences of all siRNAs used in this study are listed in Supplemental Table 2.

### Cell viability and synergy score.

Approximately 3 × 10^3^ to 5 × 10^3^ cells were seeded per well in a 96-well plate and incubated overnight before treatment with the respective inhibitors for 3–5 days. Following treatment, the cells were incubated with medium containing 10% cell counting kit-8 (CCK-8) (Bimake) for 1–4 hours. The absorbance of the resulting solution in individual wells was measured at 450 nm using a BioTek Synergy HT Microplate Reader. The IC_50_ values were calculated by normalizing them to vehicle control using Prism8 software (GraphPad).

A checkerboard assay was performed to assess the synergistic effect of 2 inhibitors. SynergyFinder Plus (https://tangsoftwarelab.shinyapps.io/synergyfinder/) was then used to calculate the synergy score, utilizing 4 computational models (HSA, Loewe, Bliss, and ZIP) (60).

### Immunoblotting.

Cell lysates were subjected to immunoblotting following the standard protocol. Briefly, proteins were separated by SDS-PAGE and transferred onto nitrocellulose membranes (Millipore). After blocking with 5% skim milk in PBS, the membranes were incubated with the indicated primary antibodies overnight at 4°C. Antibodies against YAP (1:1000, sc-101199), GAPDH (1:5000, sc-47724), BCL2L11 (1:500, sc-374358), PUMA (1:500, sc-374223), SLC7A5 (1:1000, sc-374232), and ERK1/2 (1:1000, sc-514302) were from Santa Cruz Biotechnology. Antibodies against p-YAP (Ser127) (1:2000, 4911), pan-TEAD (1:2000, 13295), SLC7A5 (1:2000, 5347), LATS1 (1:1000, 3477), p-MEK1/2 (Ser217/221) (1:2000, 9154), MEK1/2 (1:2000, 9122), p-ERK1/2 (Thr202/Tyr204) (1:2000, 9101), p-RSK (Thr359) (1:2000, 8753), RSK (1:2000, 9355), p-S6 (Ser235/236) (1:2000, 2211), S6 (1:2000, 2217), p-AKT (Ser473) (1:2000, 4060), and AKT (1:2000, 9272) were supplied by Cell Signaling Technology. Antibody against TAZ (1:2000, HPA007415) was purchased from Sigma-Aldrich. Antibodies against β-actin (1:20,000, HOA013BA01) or HA (1:5000, HOA012HA01) were from Shanghai HuiOu Biotech Co., Ltd. Antibodies against lamin A/C (1:5000, PTM-5746) were purchased from PTM BIO. After 3 washes with PBS containing 0.1% Tween 20 (PBST), appropriate horseradish peroxidase–conjugated secondary antibodies (anti-mouse: 1:20,000, HOA024GM01; anti-rabbit: 1:20,000, HOA024GR01, Shanghai HuiOu Biotech Co., Ltd) were applied for 1 hour at room temperature. The protein bands were visualized by a ChemiScope 5300 (CLINX) with ultra-sensitive ECL chemiluminescent substrate (Biosharp). β-Actin, GAPDH, or ponceau S staining was used for normalization.

### RT-qPCR.

Total RNA was extracted from cells using the RNA Extraction Kit (Foregene) according to the manufacturer’s instructions. Subsequently, the extracted RNA was reverse transcribed into cDNA using PrimerScript RT Master Mix (Takara) and subjected to RT-qPCR using RealStar Green Fast Mixture (Genstar) on a Bio-Rad CFX384 Real-Time PCR system. *ACTB* was used as the internal reference for normalization, and the relative expression levels of respective genes were calculated using the ΔΔCT method. The primer sequences used in this study are listed in Supplemental Table 3.

### Study approval.

All experiments were conducted following the protocol (2021-0739) for mouse procedures approved by the Institutional Animal Care and Use Committee of The First Hospital of Jilin University.

### Statistics.

Unless stated otherwise, the experiments were performed with at least 3 independent replicates, consistently yielding similar results. The number of mice in each experiment is described in the figure legends. All statistical analyses were conducted using R (version 4.3.0). Mean values are presented, with error bars indicating either the standard error of the mean (SEM) or standard deviation (SD), as specified in the figure legends. Box-and-whisker plots are presented with medians, quartiles, and whiskers that extend to 1.5 times the interquartile range. A 2-tailed unpaired Student’s *t* test was used to compare 2 groups. One-way analysis of variance (ANOVA) followed by Tukey’s test was conducted to compare 3 or more groups. A *P* value of less than 0.05 was considered statistically significant. The corresponding figure legends provide detailed information regarding the statistical tests for each experiment.

### Data availability.

The cell line drug sensitivity data used in this study were obtained from the GDSC and PRISM Repurposing datasets (33, 61). RNA-seq and mutation data for cell lines were sourced from DepMap (34). The CRISPR screening datasets were derived from previous studies (9, 13, 42–44). The public dataset that supports the findings of this study is available in the NCBI Gene Expression Omnibus under accession codes GSE178479, GSE164326 (40), GSE179212 (41), GSE152737 (45), GSE165631 (46), GSE157717 (47), GSE161010 (48) for bulk RNA-seq, and GSE137912 (43) for scRNA-seq. All other data supporting the findings of this study are available from the correspondence upon reasonable request. Values for all data points in graphs are reported in the Supporting Data Values file.

### Code availability.

This study did not utilize custom algorithms.

## Author contributions

XZ, JC, and WY conceptualized the project. WY conducted most of the experiments, interpreted the data, and conducted statistical analyses. MZ, MWH, and JHL supported cell culture and immunoblotting. TXZ and HG assisted with animal husbandry and mouse xenograft experiments. XY contributed to immunohistochemistry. JC and XZ supervised the study. The manuscript was drafted initially by XZ and WY and reviewed and edited by XZ, JC, and QYL. All authors read and provided comments on the manuscript.

## Supplementary Material

Supplemental data

Unedited blot and gel images

Supplemental tables 1-5

Supporting data values

## Version 1 12/20/2024

Electronic publication
