# Supplementary material for: YAP/TAZ mediates resistance to KRAS inhibitors through inhibiting proapoptosis and activating the SLC7A5/mTOR axis
Source: JCI Insight. 2024 Dec 20;9(24):e178535. doi: 10.1172/jci.insight.178535 (PMC11665569; doi:10.1172/jci.insight.178535)
Supplement: Supplemental data [file jciinsight-9-178535-s116.pdf]

Supplementary Materials for

YAP/TAZ mediates resistance to KRAS inhibitors through inhibiting pro-apoptosis and activating SLC7A5-mTOR axis

Wang Yang[1,2,3], Ming Zhang[2,3], Tian-Xing Zhang[4], Jia-Hui Liu[2,3], Man-Wei Hao[4], Xu Yan[5], Haicheng Gao[4], Qun-Ying Lei[6,7,8], Jiuwei Cui[1,*], Xin Zhou[1,2,3,*]

[1]Cancer Center, The First Hospital of Jilin University, Changchun 130021, China

[2]Cancer Research Institute of Jilin University, The First Hospital of Jilin University, Changchun 130021, China

[3]International Center of Future Science, Jilin University, Changchun 130021, China

[4]School of Pharmaceutical Sciences, Jilin University, Changchun 130021, China

[5]Pathological Diagnostic Center, The First Hospital of Jilin University, Changchun 130021, China

[6]Fudan University Shanghai Cancer Center & Institutes of Biomedical Sciences; School of Basic Medical Sciences; Cancer Institutes; Key Laboratory of Breast Cancer in Shanghai; Shanghai Key Laboratory of Radiation Oncology; The Shanghai Key Laboratory of Medical Epigenetics, Shanghai Medical College, Fudan University, Shanghai 20032, China.

[7]Department of Oncology, Shanghai Medical College, Fudan University, Shanghai 20032, China.

[8]State Key Laboratory of Medical Neurobiology, Fudan University, Shanghai 20032, China.

Address correspondence to:

Prof. Jiuwei Cui, Cancer Center, The First Hospital of Jilin University, Changchun 130021, China; Email: cuijw@jlu.edu.cn;

Prof. Xin Zhou, Cancer Center, The First Hospital of Jilin University, Changchun 130021, China; Email: xinzhou@jlu.edu.cn

Supplemental methods

Xenograft mouse studies

Female BALB/c nude mice aged 6-8 weeks were purchased from Vital River Laboratories in Beijing. The mice were group-housed at The First Hospital of Jilin University's animal facility under pathogen-free conditions. The housing environment maintained a 20-26°C temperature range, 40-60% humidity, and a 12-hour light/12-hour dark cycle.

For the SW1573 xenograft study, a suspension of SW1573 cells was mixed with Matrigel (Corning) in a volume ratio of 2:1. For the H358R xenograft study, a suspension of H358R cells was mixed with Matrigengel (ABW), also in a ratio of 2:1 for volume. BALB/c nude mice were then inoculated with SW1573 (1×10^7) or H358R (5×10^6) cells in the right flank for their respective studies. All the studies allowed the tumors to grow to a particular volume before initiating treatment. After that, the mice were randomly assigned to different treatment groups to ensure that the average baseline tumor sizes were comparable across various treatment regimens. The tumor volumes and body weights of the mice were measured approximately every 3-4 days.

To prepare the drug formulations, AMG510, dasatinib, and MYF-03-176 were added to a 0.5% solution of carboxymethylcellulose sodium salt (Sigma-Aldrich) to create a homogeneous suspension. Simvastatin, on the other hand, was mixed with 30% PEG300, 5% Tween80, and 2% DMSO. AMG510, dasatinib, and MYF-03-176 were administered orally, while simvastatin was administered via intraperitoneal injection. The treatment strategies for the different experiments are as follows: For the SW1573

xenograft mice studies, one experiment involved daily administration of 60 mg/kg of AMG510, 30 mg/kg of dasatinib, or both in combination for 21 days. In another experiment, the mice were given 60 mg/kg/day of AMG510, 100 mg/kg twice per day of MYF-03-176, or both in combination for 21 days. Additionally, there was a separate experiment where the mice received administration of 60 mg/kg/day of AMG510, 10mg/kg/day of simvastatin, or both in combination for seven days, followed by a switch to 60 mg/kg/day of AMG510, 20mg/kg/day of simvastatin, or both in combination for another 18 days. For the H358R xenograft study, mice received daily treatment of 30 mg/kg of AMG510, 30 mg/kg of dasatinib, or both in combination for the first 12 days. Subsequently, a treatment regimen of 10 mg/kg/day of AMG510, 30 mg/kg twice per day of dasatinib, or both in combination was followed for the remaining ten days (control and dasatinib groups) or 16 days (AMG510 and combination groups).

Cell apoptosis

After the knockdown of YAP/TAZ or TEADs in SW1573 and H2030 cells by siRNA for 48 hours, the cells were reseeded at a density of 1 x 10^5 cells per well in 12-well plates. After overnight incubation, the cells were treated with five days of KRAS G12C inhibitors (ARS1620: 1 uM; AMG510: 0.1 uM; MRTX849: 0.1 uM) or DMSO. The cells were then stained using the Annexin V-FITC/7-AAD Apoptosis Kit (Elabscience) and analyzed on a FACSAria II flow cytometry system (BD) with the assistance of FlowJo10 software.

EdU incorporation

Following the knockdown of YAP/TAZ or TEADs in KYSE410, H2030, and SW1573 cells by siRNA for 48 hours, the cells were reseeded at a density of 3 x 10^4 cells per well in 24-well plates. The cells were incubated overnight before being treated with or without KRAS G12C inhibitors (ARS1620: 1 uM; AMG510: 0.1 uM; MRTX849: 0.1 uM) for five days. Afterward, the cells were incubated with 50 uM of EdU for two hours and stained according to the protocol of the Cell-Light EdU Apollo567 In Vitro Kit (RiboBio). Images were captured using an Olympus IX73 inverted microscope and analyzed using ImageJ software. The proliferation rate was determined by dividing EdU-positive cells by the total number of cells stained with DAPI.

Clonogenic assay

Approximately 3-5 x 10^3 cells were seeded per well in 6/12-well plates and incubated overnight. The cells were subsequently treated with respective inhibitors in a regular culture medium for 10-14 days, with media and inhibitors changes every 3-4 days. Afterward, the cells were fixed and stained using a 0.5% crystal violet solution containing 10% methanol. Once the plate was dry, images were captured, and the areas of the clones were quantified using ImageJ software. All survival fractions were calculated relative to the vehicle control.

Immunofluorescence

Approximately 3 x 10^4 cells were plated in each 15 mm glass-bottom cell culture dish (NEST). After washing with PBS, the cells were fixed with 4% paraformaldehyde (Biosharp) for 15 minutes and then permeabilized with 0.5% Triton-X-100 for 10 minutes at room temperature. Subsequently, the cells were blocked with PBST containing 2% BSA at room temperature for 1 hour. Primary antibodies against YAP (1:200, sc-101199, Santa Cruz) or TAZ (1:200, HPA007415, Sigma-Aldrich) were added to the cells and incubated overnight at 4°C. The cells were washed with PBST three times and incubated with appropriate Alexa Fluor conjugated secondary antibodies (1:1000, A32790, Invitrogen; 1:1000, A32773, Invitrogen) at room temperature for 1 hour. After three washes with PBST, the cells were stained with DAPI (Servicebio) at room temperature for 10 minutes. Finally, images were captured using an Olympus FV3000 confocal microscope and analyzed by ImageJ software.

Tissue processing and immunohistochemistry

The tumor tissues were fixed in 4% paraformaldehyde for 24 hours, followed by dehydration in a gradient of ethanol until immersion in 100% ethanol. Subsequently, the samples were embedded in paraffin and sliced to a thickness of 5 um on a microtome (Leica). Immunohistochemistry was conducted following the standard protocol. Briefly, the slides were deparaffinized in xylene and rehydrated with an ethanol gradient. Antigen retrieval was performed by subjecting the slides to high-pressure boiling in citrate buffer (pH 6.0) (ZSGB-BIO). The slides were then quenched with 3% H_2O_2 for 20 minutes and blocked with PBST containing 10% goat serum for 10 minutes at room

temperature. The slides were then incubated overnight at 4°C with antibodies against Ki67 (1:400, PTM-5032, PTM BIO) or cleaved caspase-3 (Asp175) (1:200, 9664, Cell Signaling Technology). After three washes with PBST, the slices were incubated with a rabbit-enhanced polymer detection system (ZSGB-BIO) and detected with a DAB kit (ZSGB-BIO). Finally, after counterstaining with hematoxylin, the slices were dehydrated and mounted with a coverslip (Servicebio). Images were captured using a digital pathology scanner (Olympus or Motic). To quantify the percentage of Ki67-positive tumor cells, each of the five independent observers accessed three microscopic views per slide, and a median value was taken for further analysis.

Correlation analysis of sensitivities between KRAS (G12C) Inhibitor-12 and other inhibitors in KRAS G12C-mutant cell lines

In KRAS G12C-mutant cell lines, the correlation between the IC50 values of KRAS (G12C) Inhibitor-12 and the IC50 values of inhibitors targeting RTKs, MAPK, or PI3K/mTOR was assessed using the Spearman correlation coefficient, based on information provided by GDSC2 dataset (1). Inhibitors with correlation coefficients greater than 0.5 and *P* value less than 0.05 were considered statistically significant.

Identification of regulatory factors and gene sets associated with KRAS G12C inhibitor resistance

The sensitivities of different cell lines to KRAS G12C inhibitors, namely ARS1620, AMG510, and MRTX849, were ranked based on the previous studies (2-4). Each cell

line was assigned a resistance index specific to each of these inhibitors. The resistance index ranges from 0 to 1, where a lower index indicates a higher sensitivity to KRAS G12C inhibitors, and a higher index indicates resistance. By integrating RNA-seq data from the Cancer Cell Line Encyclopedia (CCLE) (5), Spearman correlation analyses were conducted to examine the correlation between the resistance indexes of KRAS G12C inhibitors and gene expression patterns in these cell lines. Genes with a correlation coefficient greater than 0.5 and a P value less than 0.05 were considered as specific KRAS G12C inhibitor resistance-related genes. By overlapping three gene sets corresponding to ARS1620, AMG510, and MRTX849, the union of any two specific KRAS G12C inhibitor resistance-related genes was identified as pan-KRAS G12C inhibitor resistance-related genes (134 genes). These sets of genes are listed in Supplemental Table 4. Furthermore, regulatory factors associated with these genes were identified by Lisa (http://lisa.cistrome.org/) (6). A network graph depicting the interactions of the top ten regulatory factors was generated using the geneMANIA plugin in Cytoscape (version 3.9.1) (7).

Identification of gene sets associated with YAP activity

A set of genes activated by YAP upon KRAS depletion was identified previously(8). More stringent screening criteria were implemented to refine this gene set: The LacZ ratio threshold was changed from less than 0.8 to less than 0.5, and the YAP1 ratio threshold was changed from larger than 0.8 to larger than 1. This resulting refined gene set (324 genes) was named YAP UP. On the other hand, based on the aforementioned

results from Lisa, where 134 genes were identified, 39 genes with a median YAP target score larger than 0.3 were identified as YAP target genes, forming the YAP Lisa gene set (see Supplemental Table 5). To calculate the YAP UP and YAP Lisa scores in different samples, the GSVA-based ssgsea method was employed for bulk RNA-seq data, while the Seurat-based AddModuleScore method was utilized for single-cell RNA-seq data (9, 10).

Correlation analysis of YAP Lisa scores and sensitivities of individual inhibitors in various cell lines

YAP Lisa scores were calculated for each cell using CCLE RNA-seq data (5), and Spearman correlation was employed to assess the correlation between YAP Lisa scores and the IC50 values of individual inhibitors in various cell lines, based on data provided by the GDSC and PRISM Repurposing datasets (1, 11).

Identification of apoptosis-related and mTOR-related genes that are regulated by YAP/TAZ via analysis of transcriptomic profiling in multiple cell lines

To characterize YAP/TAZ activity, we classified datasets into two categories: The YAP/TAZ^{on} datasets involved YAP overexpression, TAZ overexpression, or LATS1/2 knockout, while YAP/TAZ^{off} datasets involved YAP/TAZ knockdown. Before analysis, RNA-seq data in counts format was transformed into logCPM using edgeR (12). The differential expression analysis was performed using the limma package (13) on the following datasets: GSE165631 (14) (SALE: Vector~YAP_5SA), GSE157717 (15)

(HeLa: Ctrl~LATS1/2_KO), GSE152737 (16) (SW872: Vector~YAP_5SA; Vector~TAZ_4SA), GSE161010 (17). Afterward, the expression levels of apoptosis-related and mTOR-related genes were compared across multiple cell lines upon either YAP/TAZ^{on} or YAP/TAZ^{off}.

Identification of YAP/TAZ target genes associated with survival dependency upon treatment with KRAS G12C inhibitors or MEK inhibitor via analyzing CRISPR screening data

All CRISPR screening data were sorted and scaled as CRISPR scores ranging from 0 to 1. A low CRISPR score for a particular gene indicates a increased sensitivity of cells lacking that gene to KRAS G12C inhibitors or MEK inhibitor. Conversely, a high CRISPR score suggests resistance of cells lacking that gene to KRAS G12C inhibitors or MEK inhibitor.

References

1. Iorio F, Knijnenburg TA, Vis DJ, Bignell GR, Menden MP, Schubert M, et al. A Landscape of Pharmacogenomic Interactions in Cancer. *Cell.* 2016;166(3):740-54.
2. Canon J, Rex K, Saiki AY, Mohr C, Cooke K, Bagal D, et al. The clinical KRAS(G12C) inhibitor AMG 510 drives anti-tumour immunity. *Nature.* 2019;575(7781):217-23.
3. Hallin J, Engstrom LD, Hargis L, Calinisan A, Aranda R, Briere DM, et al. The KRAS(G12C) Inhibitor MRTX849 Provides Insight toward Therapeutic Susceptibility of KRAS-Mutant Cancers in Mouse Models and Patients. *Cancer Discov.* 2020;10(1):54-71.
4. Janes MR, Zhang J, Li LS, Hansen R, Peters U, Guo X, et al. Targeting KRAS Mutant Cancers with a Covalent G12C-Specific Inhibitor. *Cell.* 2018;172(3):578-89.e17.
5. Ghandi M, Huang FW, Jané-Valbuena J, Kryukov GV, Lo CC, McDonald ER, 3rd, et al. Next-generation characterization of the Cancer Cell Line Encyclopedia. *Nature.* 2019;569(7757):503-8.
6. Qin Q, Fan J, Zheng R, Wan C, Mei S, Wu Q, et al. Lisa: inferring transcriptional regulators through integrative modeling of public chromatin accessibility and ChIP-seq data. *Genome Biol.* 2020;21(1):32.
7. Warde-Farley D, Donaldson SL, Comes O, Zuberi K, Badrawi R, Chao P, et al. The GeneMANIA prediction server: biological network integration for gene prioritization and predicting gene function. *Nucleic Acids Res.* 2010;38(Web Server issue):W214-20.
8. Shao DD, Xue W, Krall EB, Bhutkar A, Piccioni F, Wang X, et al. KRAS and YAP1 converge to regulate EMT and tumor survival. *Cell.* 2014;158(1):171-84.
9. Hänzelmann S, Castelo R, and Guinney J. GSVA: gene set variation analysis for microarray and RNA-seq data. *BMC Bioinformatics.* 2013;14:7.
10. Hao Y, Hao S, Andersen-Nissen E, Mauck WM, 3rd, Zheng S, Butler A, et al. Integrated analysis of multimodal single-cell data. *Cell.* 2021;184(13):3573-87.e29.
11. Corsello SM, Nagari RT, Spangler RD, Rossen J, Kocak M, Bryan JG, et al. Discovering the anti-cancer potential of non-oncology drugs by systematic viability profiling. *Nat Cancer.* 2020;1(2):235-48.
12. Robinson MD, McCarthy DJ, and Smyth GK. edgeR: a Bioconductor package for differential expression analysis of digital gene expression data. *Bioinformatics.* 2010;26(1):139-40.
13. Ritchie ME, Phipson B, Wu D, Hu Y, Law CW, Shi W, et al. limma powers differential expression analyses for RNA-sequencing and microarray studies. *Nucleic Acids Res.* 2015;43(7):e47.
14. Vichas A, Riley AK, Nkinsi NT, Kamlapurkar S, Parrish PCR, Lo A, et al. Integrative oncogene-dependency mapping identifies RIT1 vulnerabilities and synergies in lung cancer. *Nat Commun.* 2021;12(1):4789.
15. He L, Yuan L, Yu W, Sun Y, Jiang D, Wang X, et al. A Regulation Loop between YAP and NR4A1 Balances Cell Proliferation and Apoptosis. *Cell Rep.* 2020;33(3):108284.
16. Merritt N, Garcia K, Rajendran D, Lin ZY, Zhang X, Mitchell KA, et al. TAZ-CAMTA1 and YAP-TFE3 alter the TAZ/YAP transcriptome by recruiting the ATAC histone acetyltransferase complex. *Elife.* 2021;10.
17. Pham TH, Hagenbeek TJ, Lee HJ, Li J, Rose CM, Lin E, et al. Machine-Learning and Chemicogenomics Approach Defines and Predicts Cross-Talk of Hippo and MAPK Pathways.

Cancer Discov. 2021;11(3):778-93.

Supplemental Figure legends

Supplemental Figure 1 | KRAS G12C-mutant cancer cell lines exhibit cross-resistance to KRAS G12C inhibitors and inhibitors targeting upstream or downstream components of the RAS signaling pathway. (**A**) Representative images of clonogenic assay evaluating the growth of H358, H1373, MIAPACA2, H2030, and SW1573 cells upon treatment with buparlisib, linsitinib, gefitinib, trametinib, or SHP099 alone, as well as in combination with AMG510. (**B**) Scatter plots presenting the correlation between the IC50 values of KRAS (G12C) Inhibitor-12 and the IC50 values of 28 individual inhibitors targeting the MAPK signaling, PI3K/mTOR signaling, or RTKs in 12 KRAS G12C-mutant cell lines. (**C**) Dot plots of viability of H358, H1373, MIAPACA2, H2030, and SW1573 cells following treatment with AMG510 (0.1uM), buparlisib (1 uM), linsitinib (0.5 uM), gefitinib (2 uM), trametinib (0.1 uM), or SHP099 (4 uM) for three days. (**D**) Heatmaps displaying the expression levels of three sets of genes that exhibit positive correlations with the resistance index of ARS1620, AMG510, or MRTX849 in various KRAS G12C-mutant cancer cell lines. Data are mean ± s.d. (**C**).

Supplemental Figure 2 | YAP/TAZ is activated in cells with intrinsic and acquired resistance to KRAS G12C inhibitors. (**A**) Representative images of immunofluorescence depicting the subcellular localization of YAP/TAZ in the indicated cells. Scale bar, 50 um. (**B** and **C**) Immunoblots showing cytoplasmic/nuclear fractionation of YAP and TAZ in cells with intrinsic and acquired resistance to KRAS G12C inhibitors. Nuclear expression levels of YAP and TAZ are normalized to Lamin A/C levels and are denoted as nor.YAP and nor.TAZ. (**D**) Immunoblots depicting the expression levels and phosphorylation status of YAP, TAZ, and ERK in H2030 and SW1573 cells after treatment of AMG510 as indicated. (**E**) Immunoblots revealing the expression levels and phosphorylation status of YAP, TAZ, and ERK in H358R and H358R N20 cells following the withdrawal of AMG510 as indicated. Blots provided together were set up in parallel at the same time (**B**-**E**).

Supplemental Figure 3 | Validation of the expression levels of YAP, TAZ, and TEADs in various cell lines with ectopic expression or knockdown of the corresponding genes. (**A** and **B**) Immunoblots confirming the overexpression of YAP or TAZ in H358, H1373, and MIAPACA2 cells. (**C** and **D**) Immunoblots indicating the knockdown efficiency of YAP, TAZ, or TEADs using siRNAs in the indicated cell lines. (**E**) Immunoblots demonstrating stable knockdown of YAP and TAZ in SW1573 and H2030 cells using shRNAs. Blots provided together were set up in parallel at the same time (**A**-**E**).

Supplemental Figure 4 | YAP/TAZ is capable of inducing resistance to KRAS G12C inhibitors and are crucial for maintaining this resistance. (**A**) Representative images of clonogenic assay showing the growth of H358 and MIAPACA2 cells with or without ectopic expression of constantly activated YAP or TAZ upon treatment with KRAS G12C inhibitors as indicated. (**B**) Dose-response curves of ARS1620, AMG510, and MRTX849 depicting the relative viability of SW1573 with or without knockdown of YAP, TAZ, TEAD2, or TEAD1/3/4. (**C**) Dose-response curves of ARS1620, AMG510, and MRTX849 depicting the relative viability of H358R and H358N20 with or without knockdown of YAP/TAZ or TEADs. (**D**) Representative images of clonogenic assay showing the growth of SW1573 and H2030 cells with or without knockdown of YAP/TAZ upon treatment with the indicated KRAS G12C inhibitors. (**E** and **F**) Representative images (**E**) and quantifications (**F**) of clonogenic assay presenting the growth of SW1573 with or without ectopic expression of LATS1 upon treatment with the indicated KRAS G12C inhibitors. (**G**) Immunoblots confirming the overexpression of LATS1 in SW1573 cells. Data are mean ± s.d. (**F**). Data are mean ± s.e.m. (**B** and **C**).

Supplemental Figure 5 | YAP/TAZ is capable of inducing resistance to MEK, PI3K, and RTK inhibitors but not SHP2 inhibitor. (**A** and **B**) Representative images (**A**) and quantifications (**B**) of clonogenic assay presenting the growth of H358, H1373, and MIAPACA2 cells with or without ectopic expression of constantly activated YAP or

TAZ (pMCB-TAZ S89A) upon treatment with increasing concentrations of buparlisib, linsitinib, gefitinib, trametinib, or SHP099. Data are mean ± s.d. (**B**).

Supplemental Figure 6 | Q-VD-OPh and BCL2L11/BMF/PUMA knockdown reverse the apoptosis induced by the combination of YAP/TAZ inhibition and KRAS G12C inhibitors. (**A**) Scatter plots showing apoptosis rates in SW1573 and H2030 cells treated with or without YAP/TAZ siRNA, KRAS G12C inhibitor, and Q-VD-OPh (20uM) as indicated for 72 hours. (**B**) Scatter plots showing apoptosis rates in SW1573 and H2030 cells treated with or without YAP/TAZ siRNA, KRAS G12C inhibitor, and BCL2L11/BMF/PUMA siRNA as indicated for 72 hours.

Supplemental Figure 7 | The knockdown of YAP/TAZ potentiates proliferation retardation by KRAS G12C inhibitors via suppression of mTOR reactivation. (**A**) Immunoblots displaying the expression levels and phosphorylation status of MEK, ERK, AKT, and S6 in H358, KYSE410, and SW1573 cells with or without knockdown of YAP/TAZ under treatment with ARS1620, AMG510, or MRTX849 for increasing duration as indicated. Blots provided together were set up in parallel at the same time (**A**).

Supplemental Figure 8 | SLC7A5 plays a crucial role in YAP/TAZ-mediated mTOR activation and KRAS G12C inhibitor resistance. (**A**) Immunoblots displaying the phosphorylation of S6 in SW1573, KYSE410, and H358R N20 cells treated with AMG510, with or without exogenous supplementation of leucine (0.4 mM) or SLC7A5 knockdown as indicated. (**B**) Immunoblots validating the ectopic expression of SLC7A5 in KYSE410 and SW1573 cells. (**C**) Immunoblots displaying the phosphorylation of S6 in SW1573 cells treated with AMG510, with or without YAP/TAZ knockdown or ectopic expression of SLC7A5 as indicated. (**D**) Box plots illustrating the quantification of proliferation rates of KYSE410, H2030, and SW1573 cells with or without knockdown of YAP/TAZ upon treatment with the indicated KRAS G12C inhibitors for five days. (**E**) Box plots illustrating the quantification of

proliferation rates of SW1573 cells with or without YAP/TAZ knockdown or SLC7A5 overexpression upon treatment with the indicated KRAS G12C inhibitors for five days. (**F**) Clonogenic assay demonstrating the synergistic effect of JPH203 and AMG510 in KYSE410, H358R N20, H358R, and H2030 cells. (**G**) Clonogenic assay assessing the effect of AMG510 in SW1573 and KYSE410 cells with or without SLC7A5 overexpression**.** Blots provided together were set up in parallel at the same time (**A**-**C**).

Supplemental Figure 9 | Multiple inhibitors targeting the Hippo-YAP/TAZ pathway enhance the efficacy of AMG510. (**A**) Bar charts illustrating the correlation between the YAP Lisa scores and the IC50 values of individual inhibitors in various cells based on the PRISM Repurposing datasets (left). Stacked bar chart presenting the distribution of indicated groups of inhibitors among all inhibitors ranked basing on Spearman correlation coefficient (right). (**B** and **C**) Representative images (**B**) and quantifications (**C**) of clonogenic assay displaying the combined effects of AMG510 with simvastatin, pitavastatin, K975, or MYF-01-37 in SW1573, H2030, and H1373 YAP S127A cells. (**D**) Representative images of clonogenic assay displaying the combined effects of AMG510 with SHP099, dasatinib, or MYF-03-176 in various cell lines, including sensitive cells (H358), acquired resistant cells (H358R and H358R N20), and intrinsically resistant cells (KYSE410, SW1573, and H2030). Data are mean ± s.d. (**C**).

Supplemental Figure 10 | Dasatinib enhances the efficacy of KRAS inhibitor primarily through its impact on YAP/TAZ inhibition. (**A**) RT-qPCR displaying the expression levels of CTGF and CYR61 in SW1573 and H358R cells following dasatinib treatment. (**B**) Immunoblotting revealing the phosphorylation status of YAP/TAZ in SW1573 cells after dasatinib treatment. (**C** and **D**) Representative images (**C**) and quantifications (**D**) of clonogenic assays displaying the synergistic effect of dasatinib combined with AMG510 in H358 and H1373 cells with or without YAP S127A or TAZ S89A overexpression. (**E**) Immunoblotting confirming the exogenous expression of YAP S127A/3YE in MIAPACA2 cells. (**F** and **G**) Representative images

(**F**) and quantifications (**G**) of clonogenic assays showing the synergistic effect of dasatinib combined with KRAS inhibitors in MIAPACA2 cells with or without overexpression of YAP mutants. Data are mean ± s.d. (**A**). Blots provided together were set up in parallel at the same time (**B** and **E**).

Supplemental Figure 11 | Targeting Hippo-YAP/TAZ pathway enhances the effectiveness of KRAS G12C inhibitor *in vivo* without causing apparent toxicities. (**A** and **B**) Representative images and quantifications of immunohistochemistry of cleaved caspase-3 (CC3) in SW1573 (**A**) and H358R (**B**) xenografts treated with AMG510, dasatinib, or both in combination. Scale bars, 60 um. (**C** and **D**) Body weight curves of SW1573 (**C**) and H358R (**D**) xenograft models treated with AMG510, dasatinib, or both in combination. n = 5 mice per group. (**E**) Tumor volume curves (left) and body weight curves (right) of SW1573 xenograft models (n = 4 mice per group) treated with AMG510, simvastatin, or both in combination. (**F**) Tumor volume curves (left) and body weight curves (right) of SW1573 xenograft models (n = 5 mice per group) treated with AMG510, MYF-03-176, or both in combination. Data are mean ± s.d. (**A** and **B**). Data are mean ± s.e.m. (**C-F**). Data were analyzed using Student's t-test, and the *P* values were adjusted using the false discovery rate (FDR) method (**A** and **B**).

Supplemental Figure 12 | Targeting YAP/TAZ enhances the effectiveness of KRAS G12D inhibitor. (**A**) Representative images of immunofluorescence depicting the subcellular localization of YAP/TAZ in KRAS G12D-mutant cell lines. Scale bar, 50 um. (**B**) Immunoblots indicating the knockdown efficiency of YAP and TAZ using siRNAs in the indicated cell lines. (**C**) Representative images of clonogenic assay displaying the combined effects of MRTX1133 with dasatinib or MYF-03-176 in HPAFII, HPAFIIR, PANC1, and SW1990 cells. Blots provided together were set up in parallel at the same time (**B**).

Supplemental Figure 13 | SLC7A5 is critical for resistance to the KRAS G12D inhibitor. (**A**) Immunoblots revealing the expression levels of SLC7A5 and ERK

phosphorylation in PANC1 cells with or without YAP/TAZ knockdown upon treatment with MRTX1133 for three days. (**B**) Immunoblots (up) confirming the exogenous expression of SLC7A5 in PANC1 cells and clonogenic assays (down) displaying the impact of SLC7A5 overexpression on the efficacy of MRTX1133 in PANC1 cells. (**C**) Clonogenic assays demonstrating a synergistic effect between JPH203 and MRTX1133 in HPAFII, LS180, and SW1990 cells. Blots provided together were set up in parallel at the same time (**A and B**).

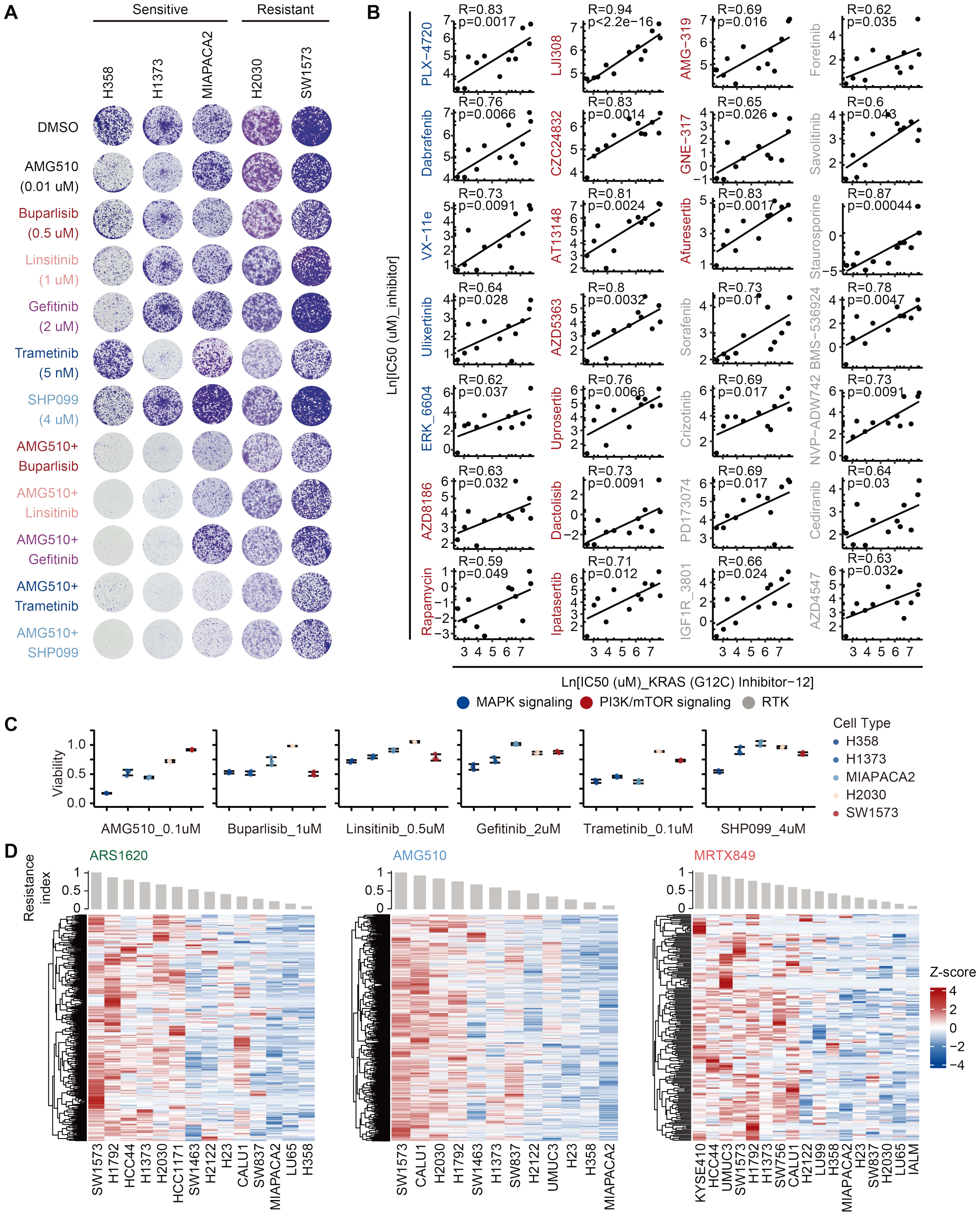

Supplemental Figure 1

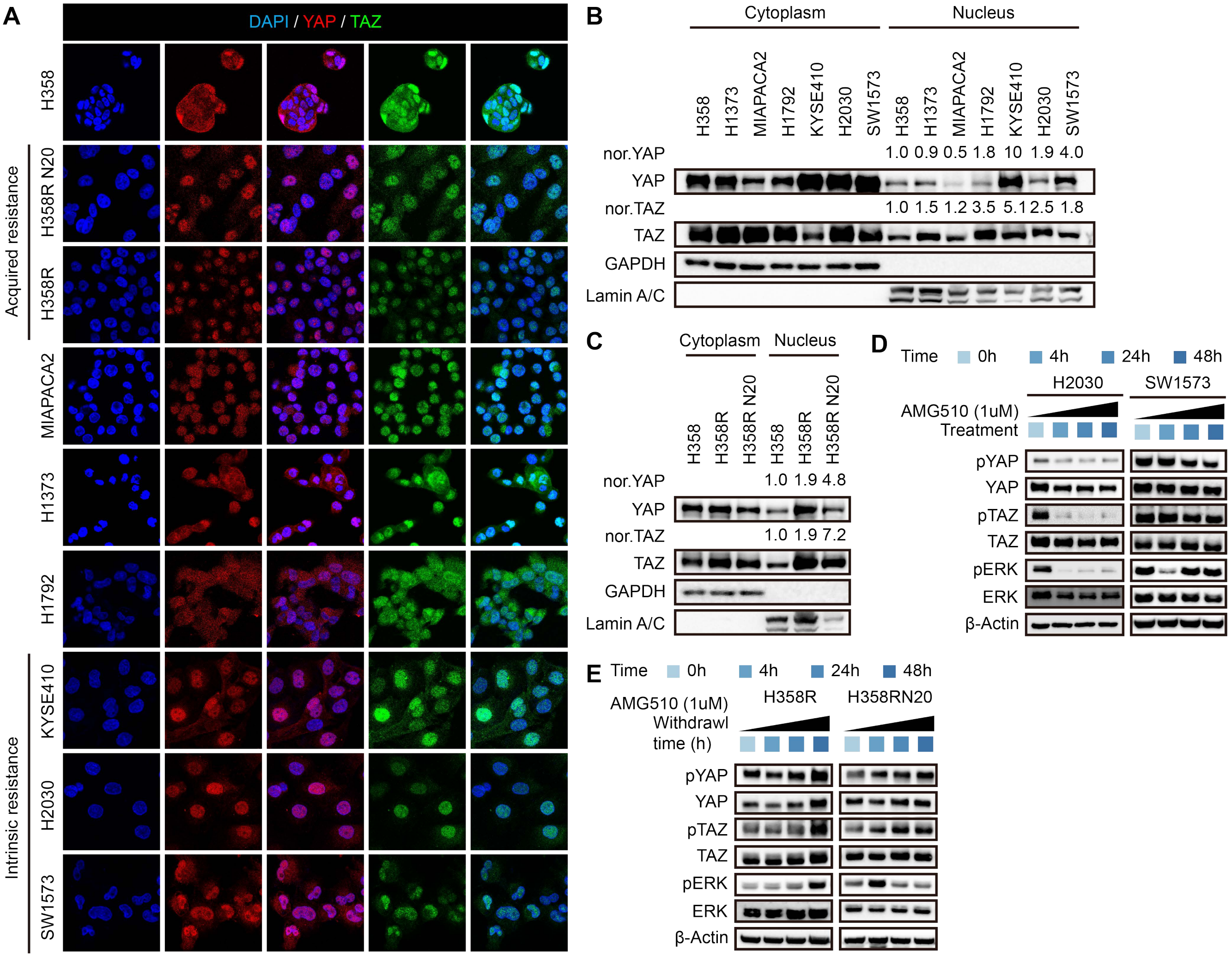

Supplemental Figure 2

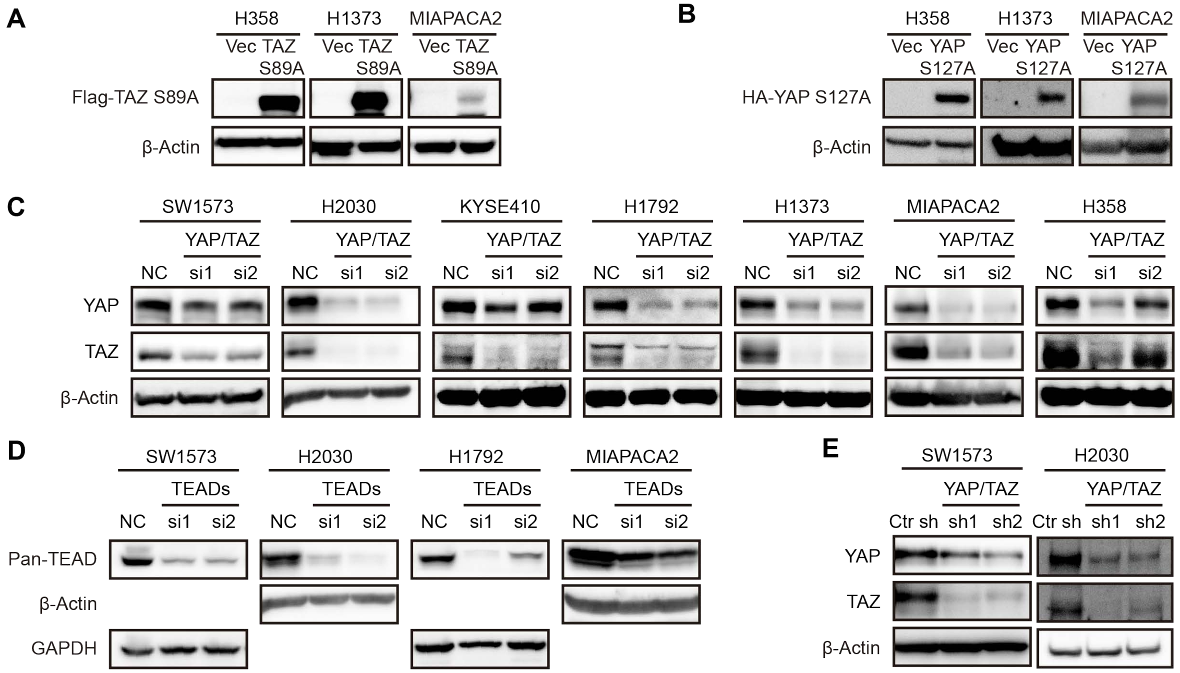

Supplemental Figure 3

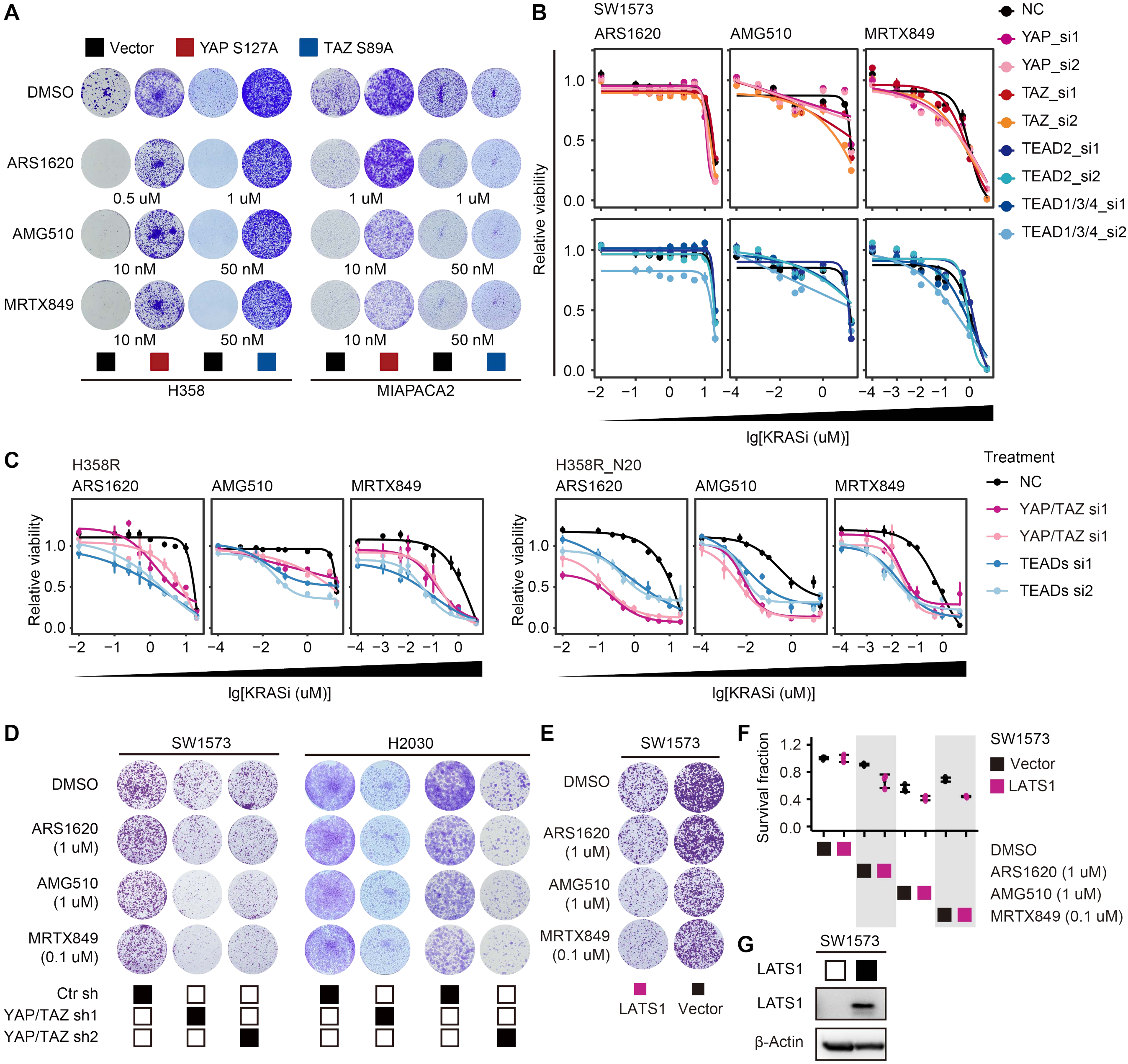

Supplemental Figure 4

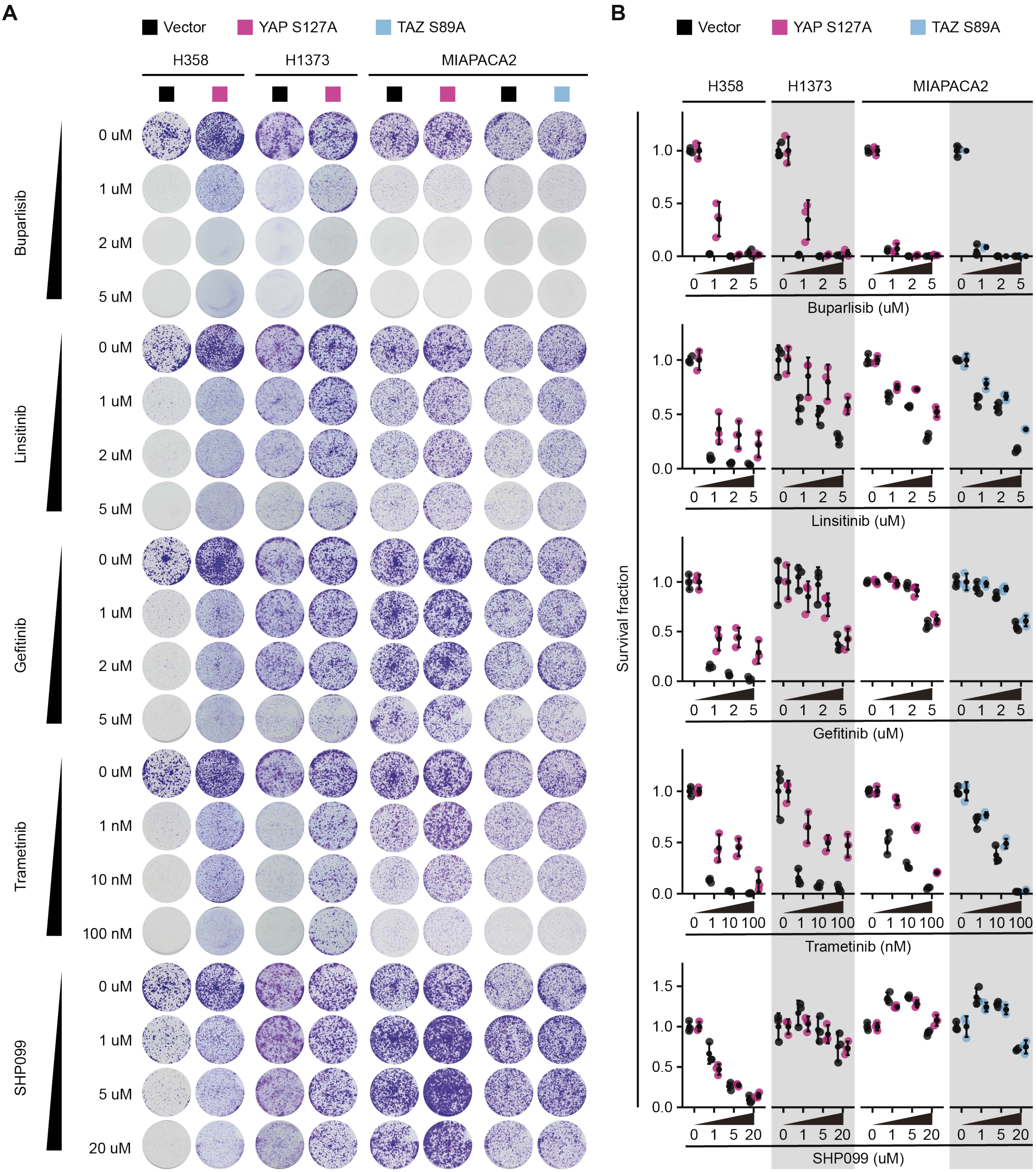

Supplemental Figure 5

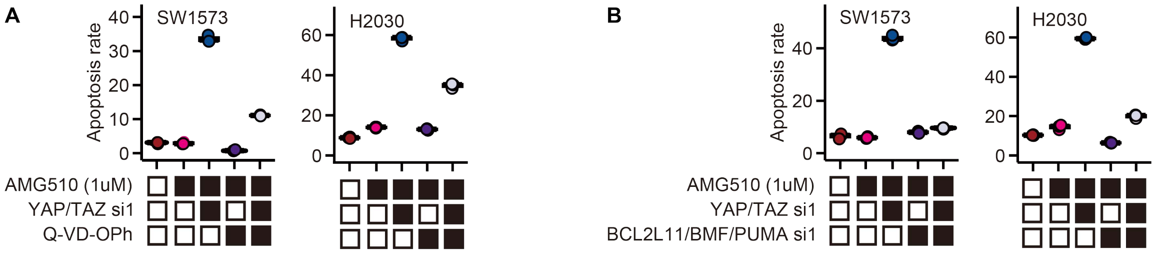

Supplemental Figure 6

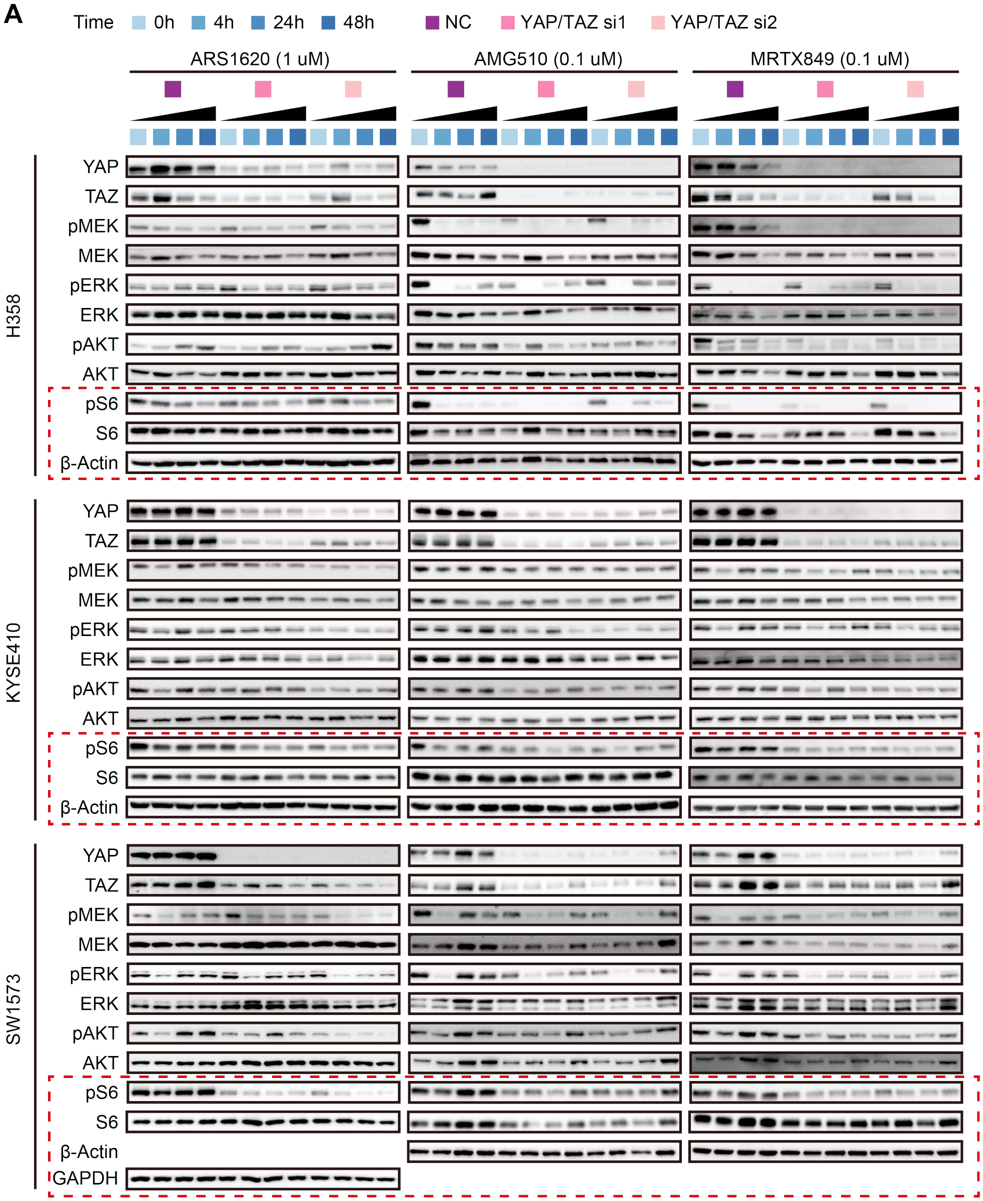

Supplemental Figure 7

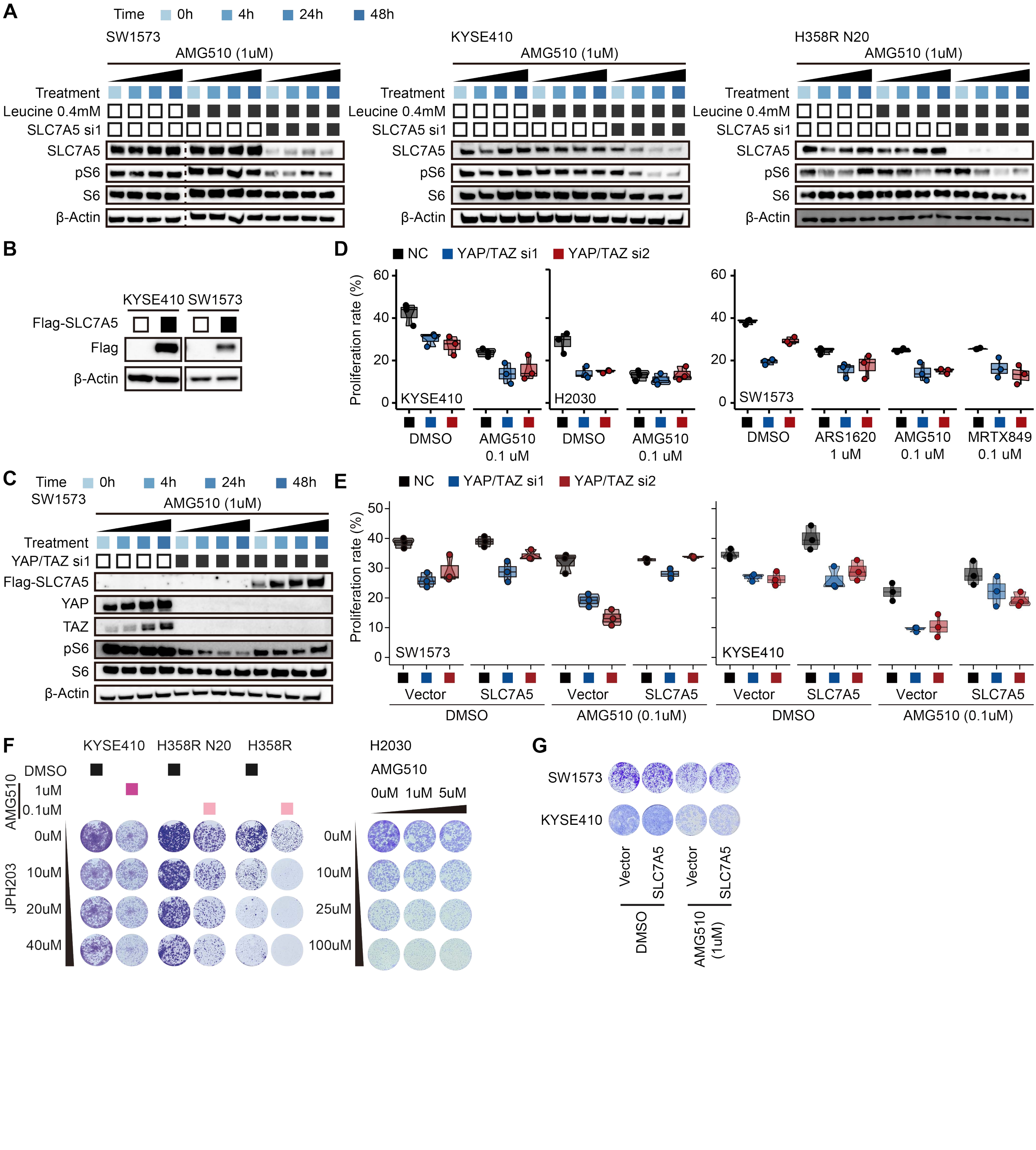

Supplemental Figure 8

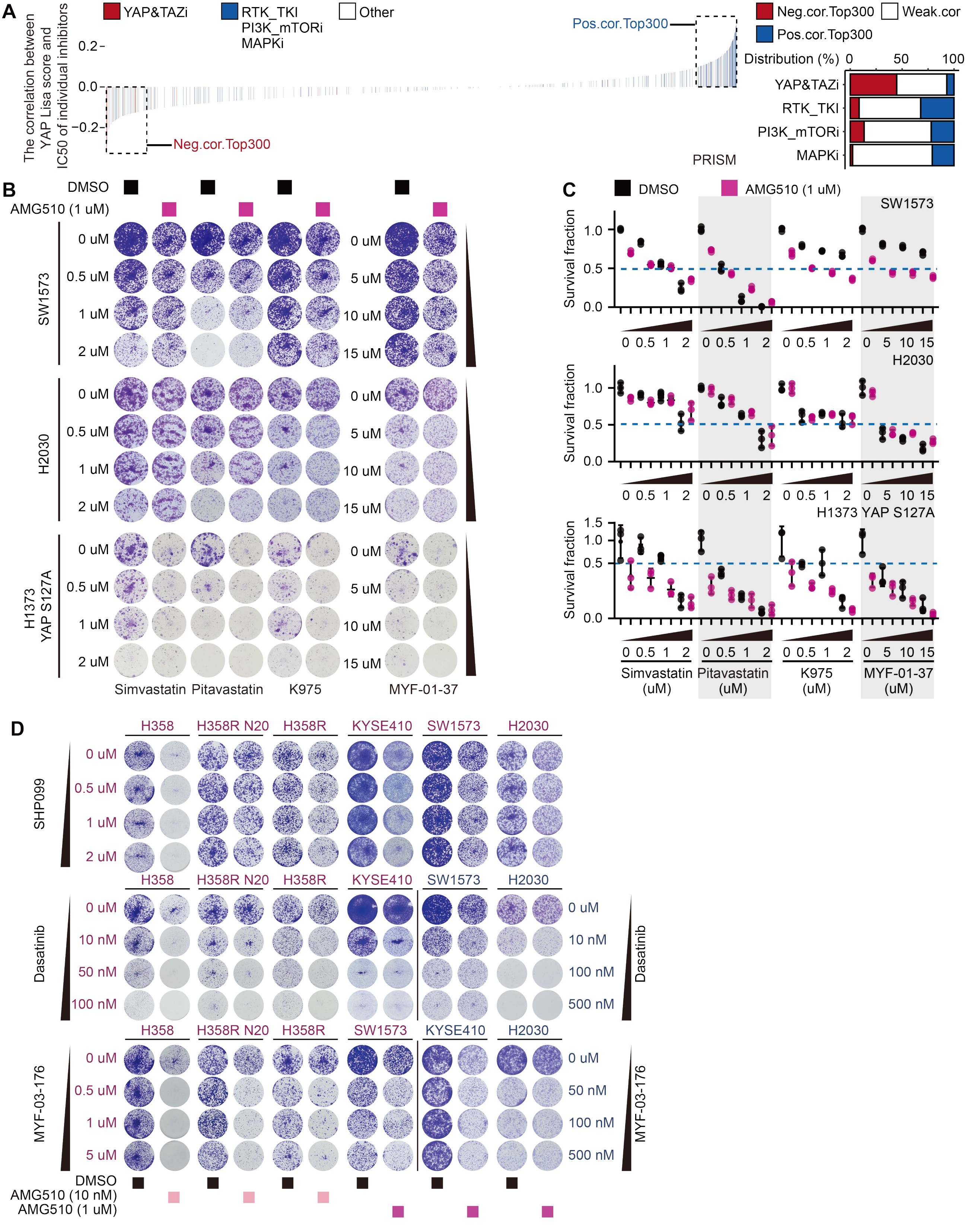

Supplemental Figure 9

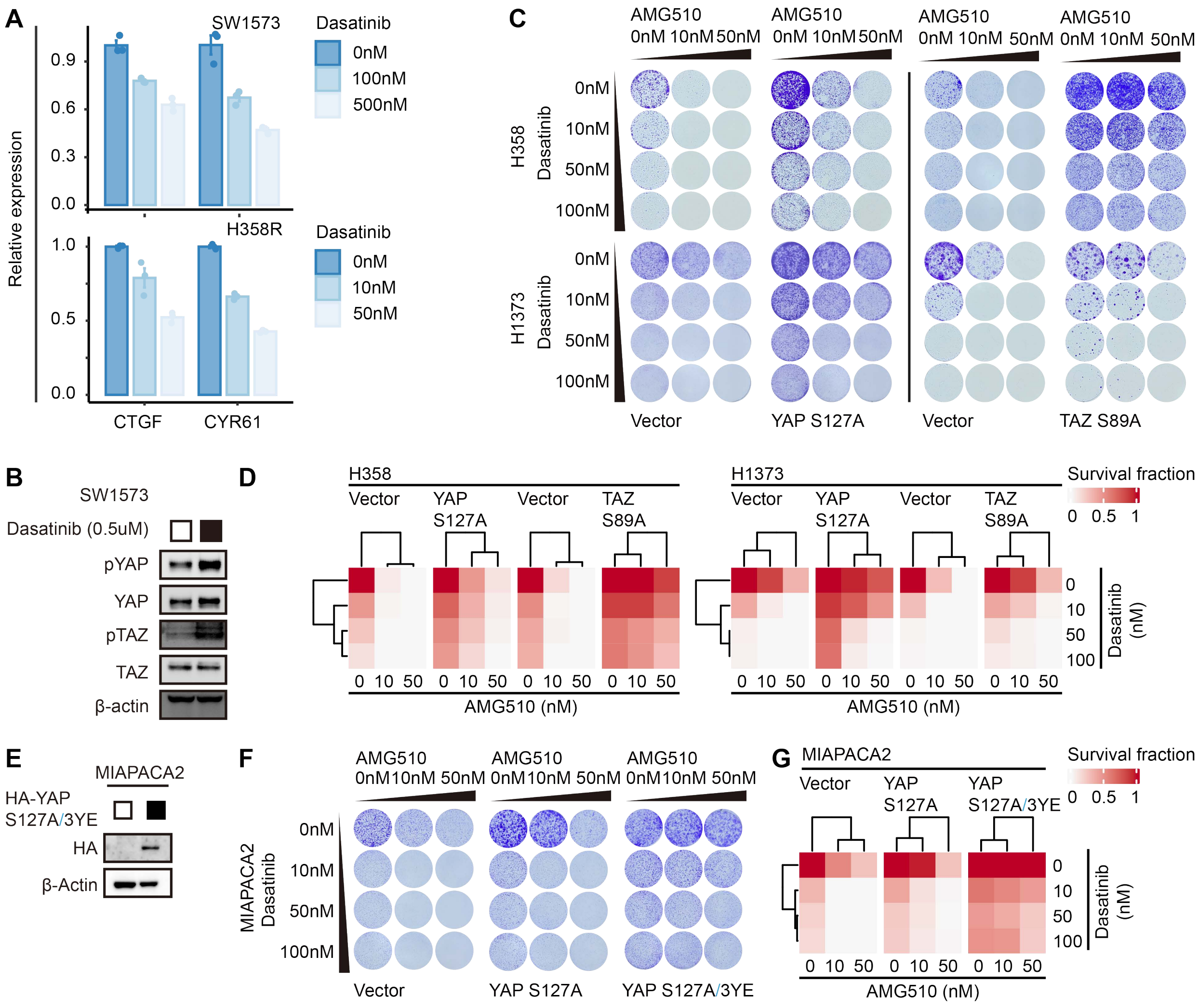

Supplemental Figure 10

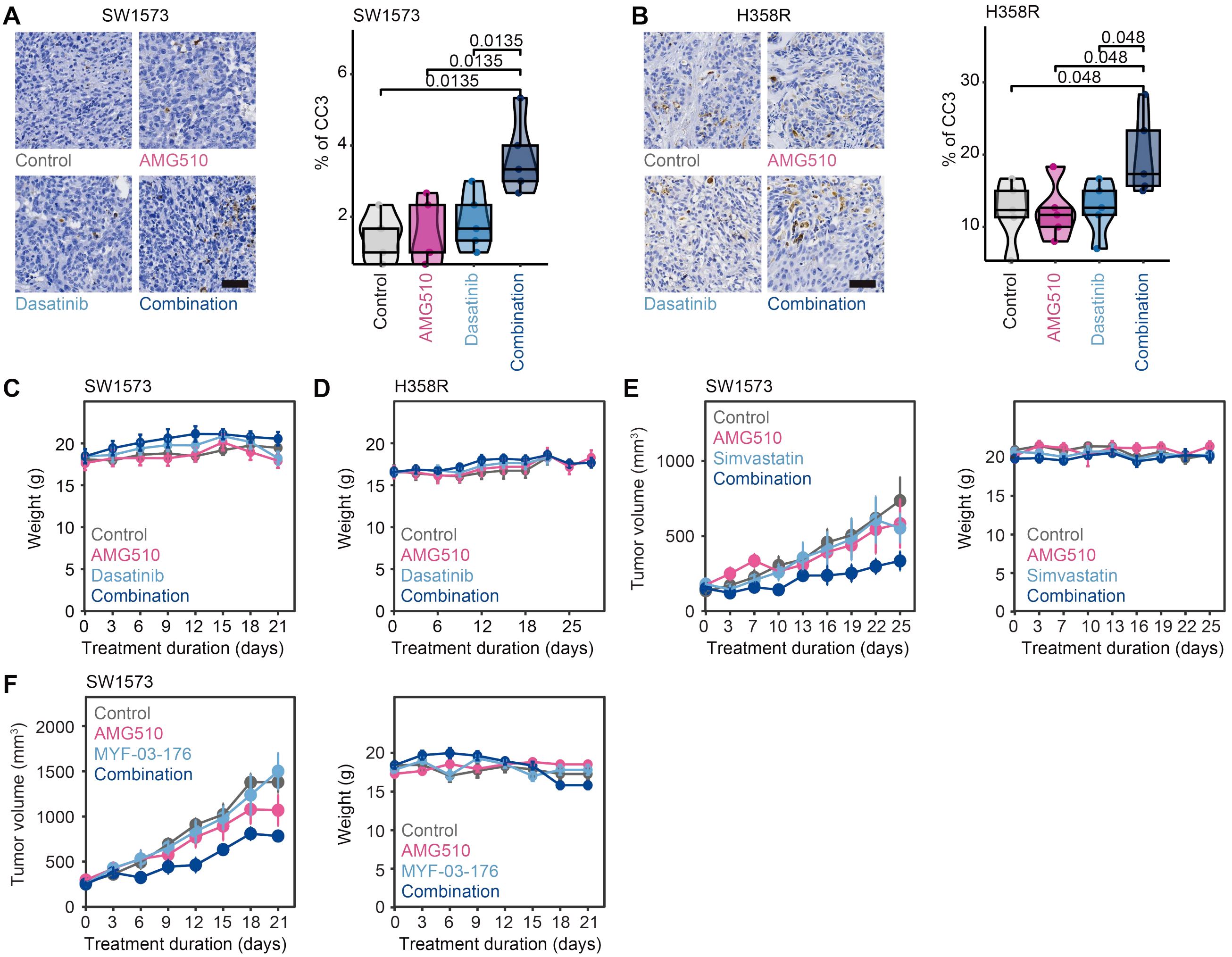

Supplemental Figure 11

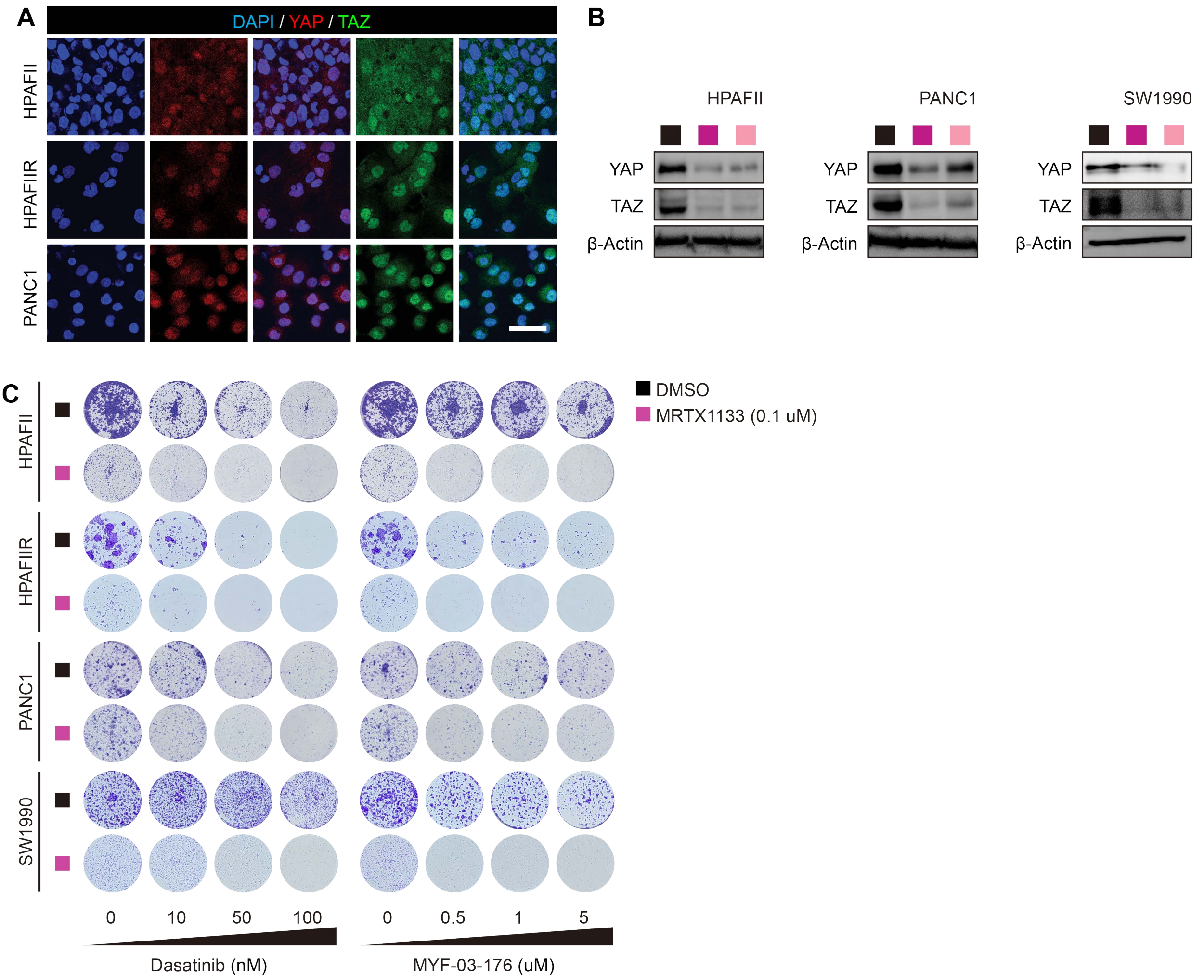

Supplemental Figure 12

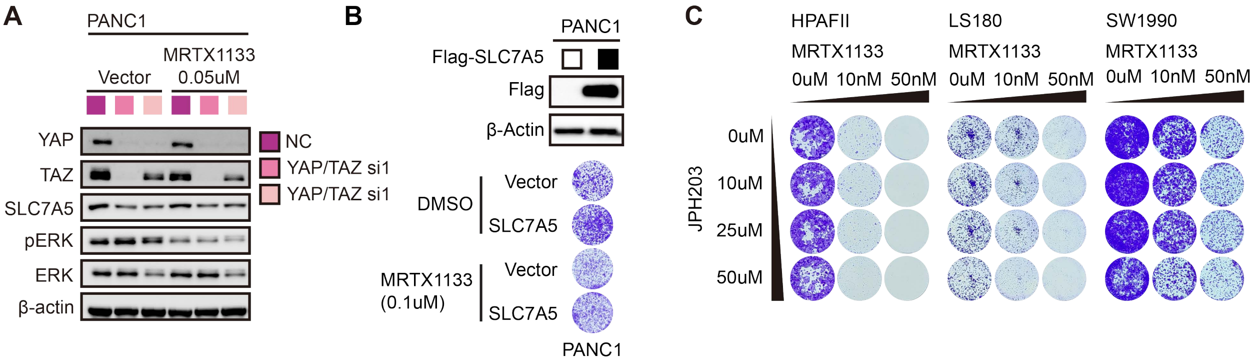

Supplemental Figure 13
