# Supplementary material for: YAP/TAZ mediates resistance to KRAS inhibitors through inhibiting proapoptosis and activating the SLC7A5/mTOR axis
Source: JCI Insight. 2024 Dec 20;9(24):e178535. doi: 10.1172/jci.insight.178535 (PMC11665569; doi:10.1172/jci.insight.178535)
Supplement: Unedited blot and gel images [file jciinsight-9-178535-s117.pdf]

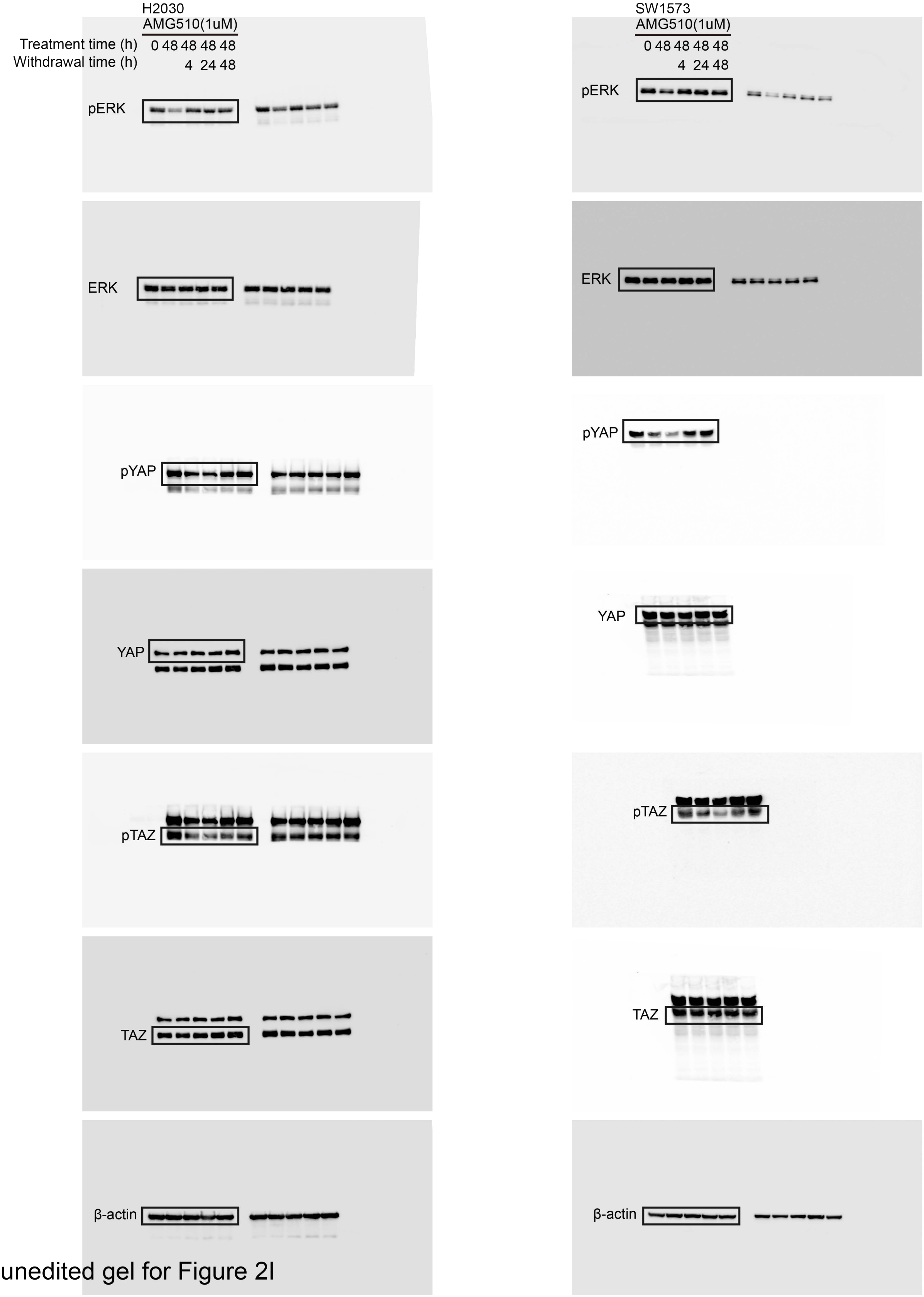

Full unedited gel for Figure 2I

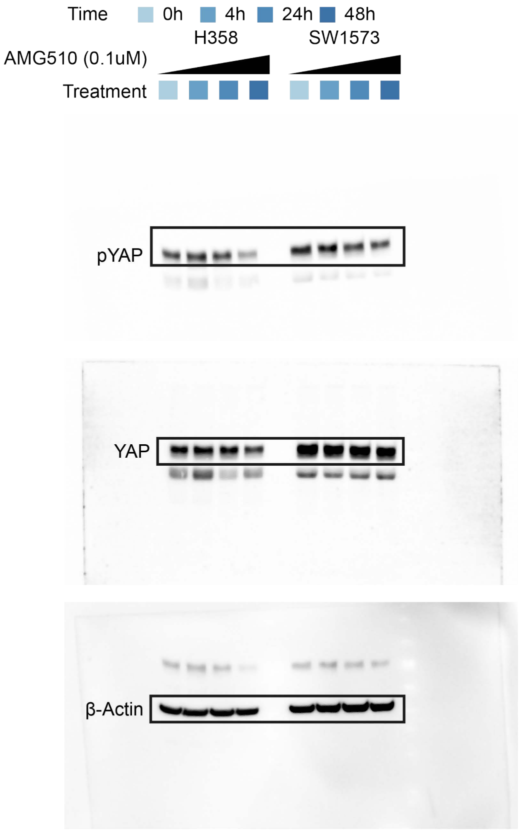

Full unedited gel for Figure 2J

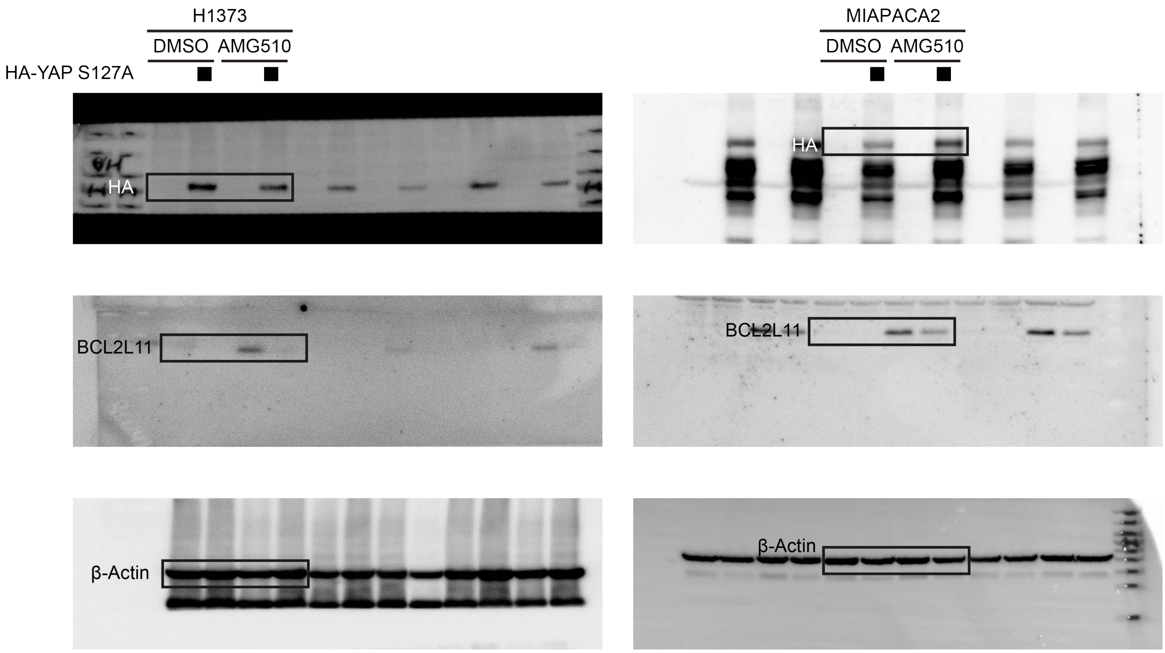

Full unedited gel for Figure 4E

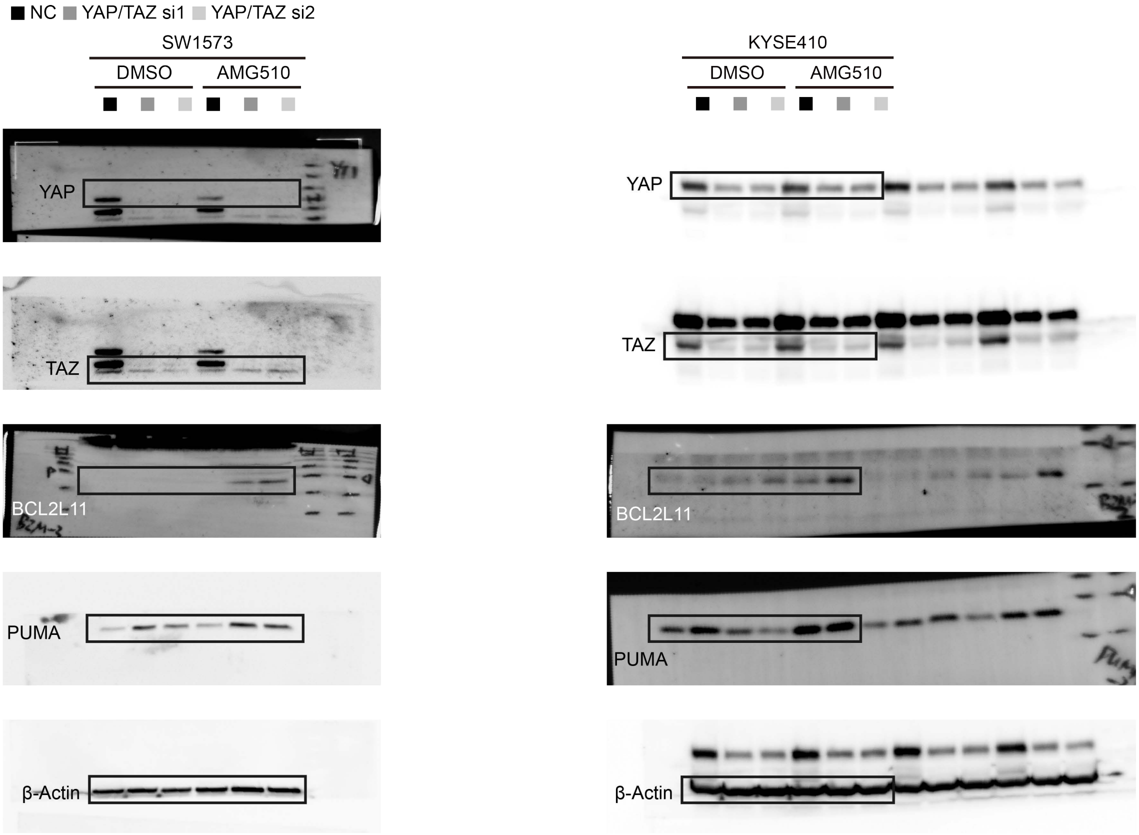

Full unedited gel for Figure 4F

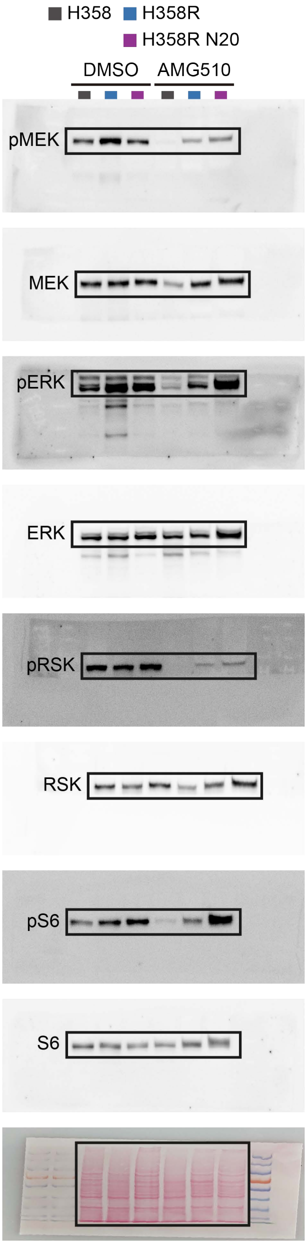

Full unedited gel for Figure 5A

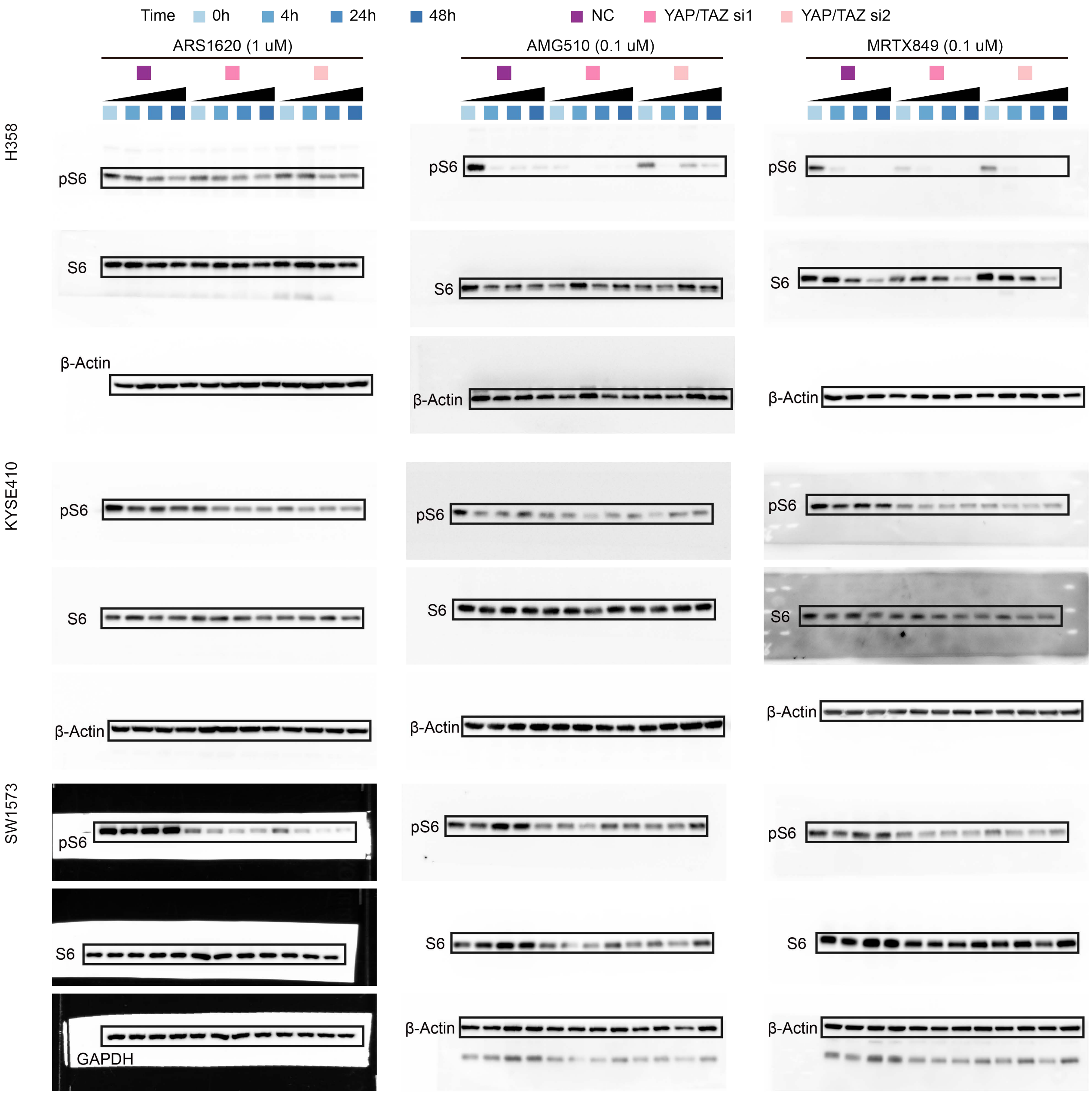

Full unedited gel for Figure 5, B-D, and Supplemental Figure 7A

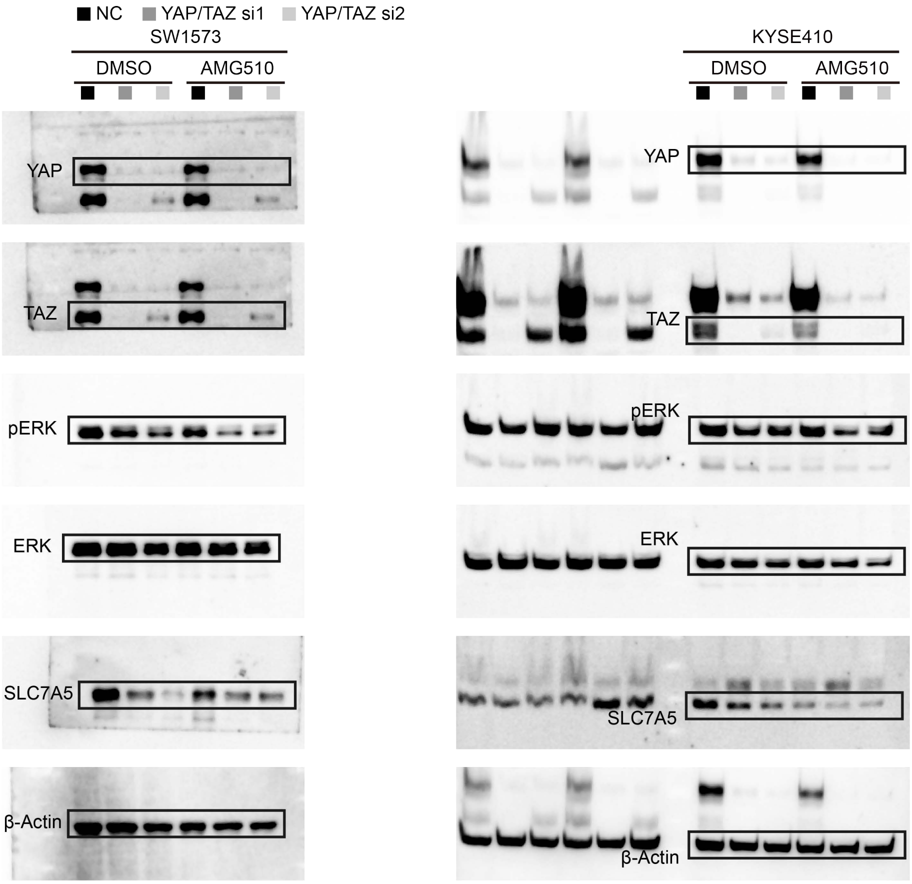

Full unedited gel for Figure 6D

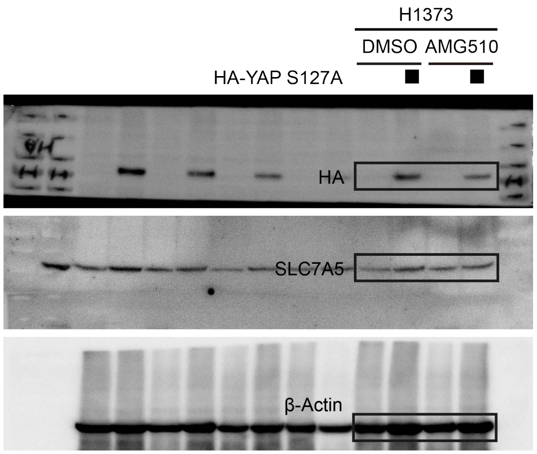

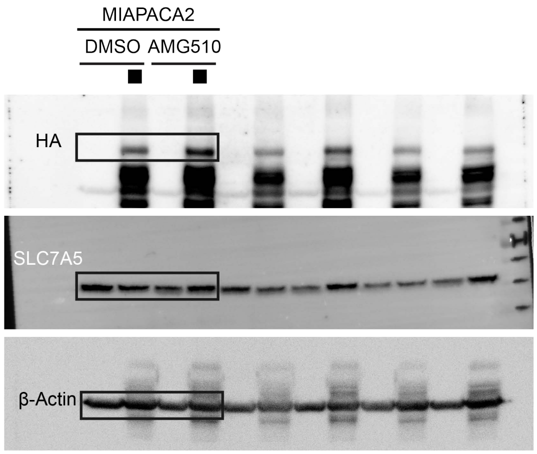

Full unedited gel for Figure 6F

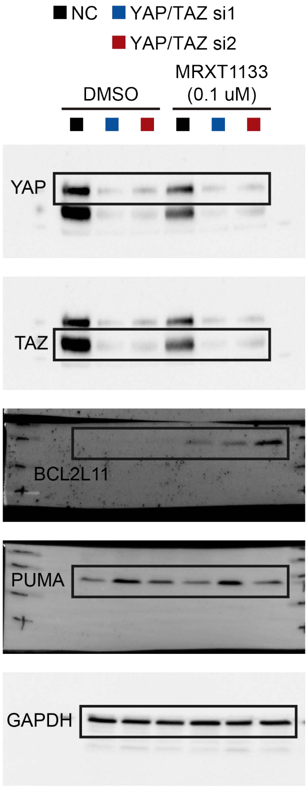

Full unedited gel for Figure 9C

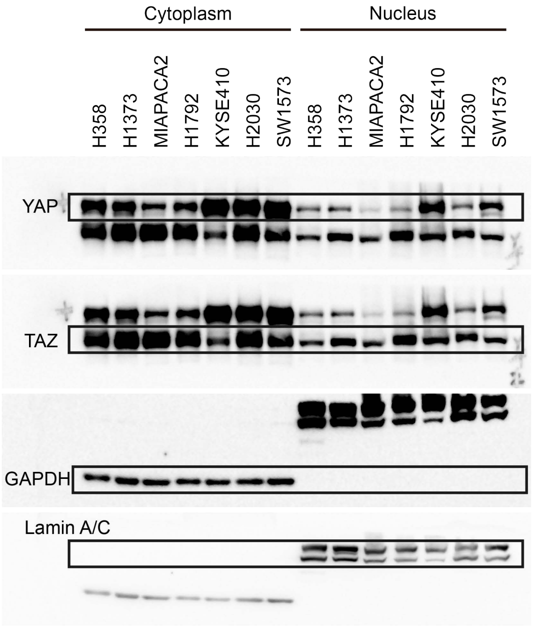

Full unedited gel for Supplemental Figure 2B

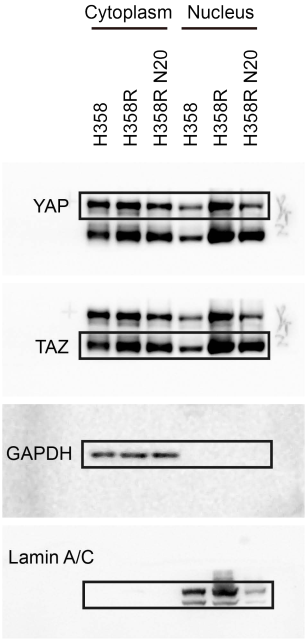

Full unedited gel for Supplemental Figure 2C

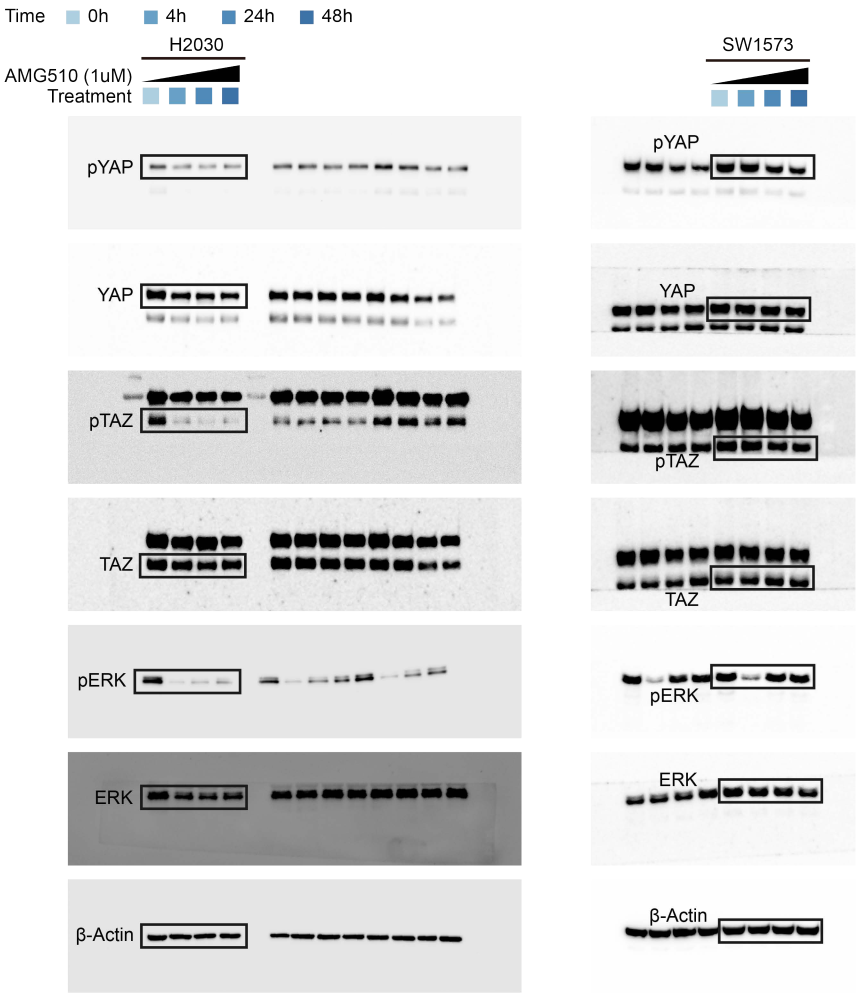

Full unedited gel for Supplemental Figure 2D

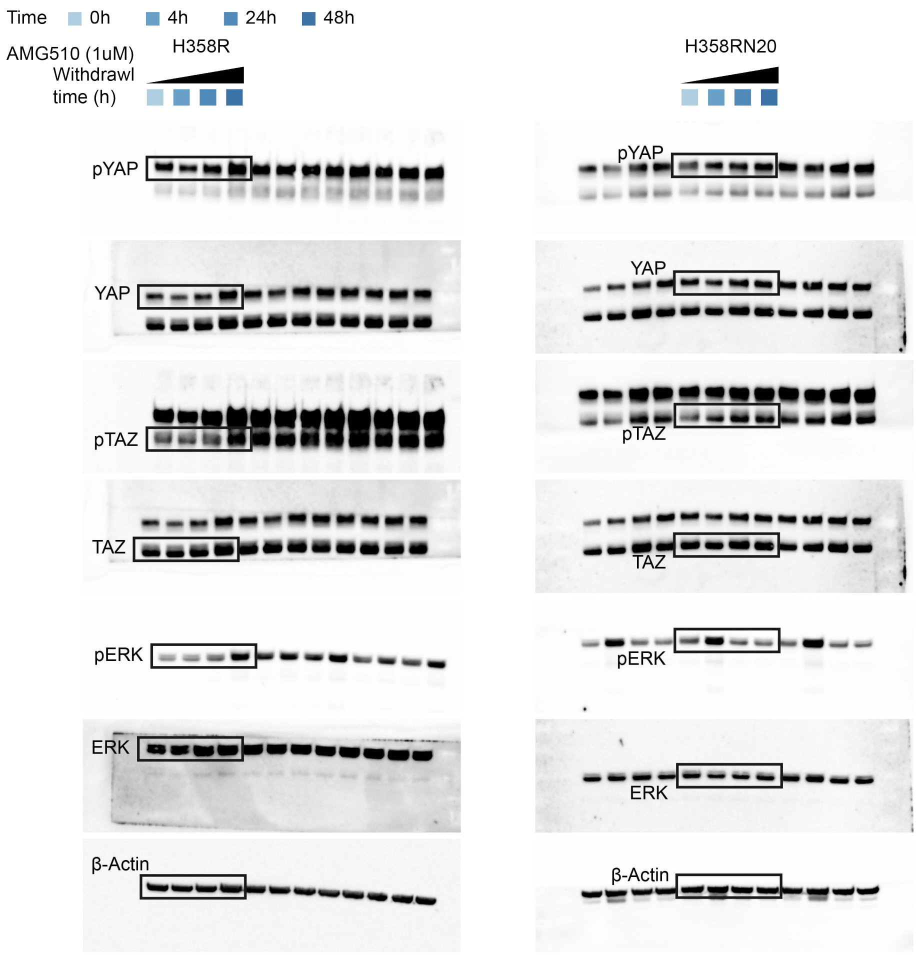

Full unedited gel for Supplemental Figure 2E

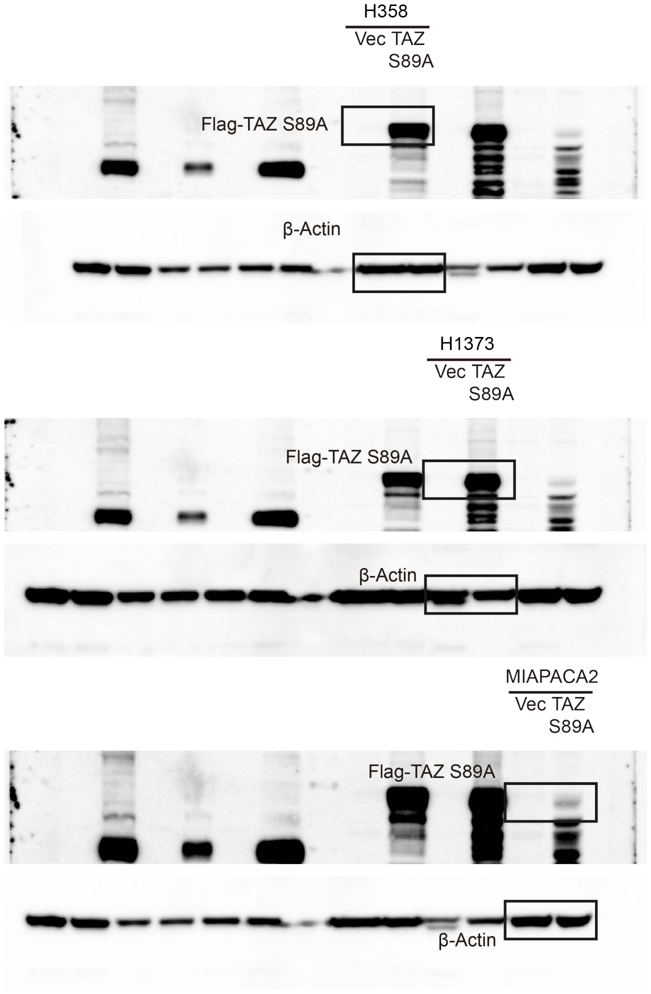

Full unedited gel for Supplemental Figure 3A

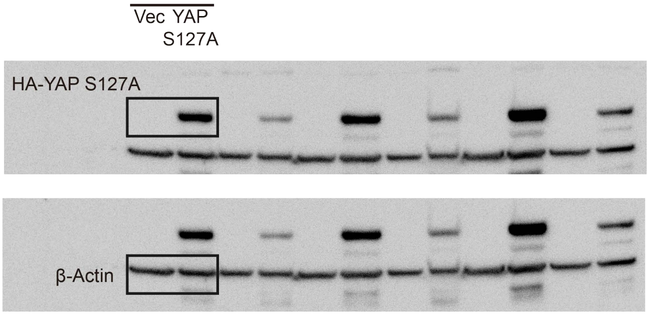

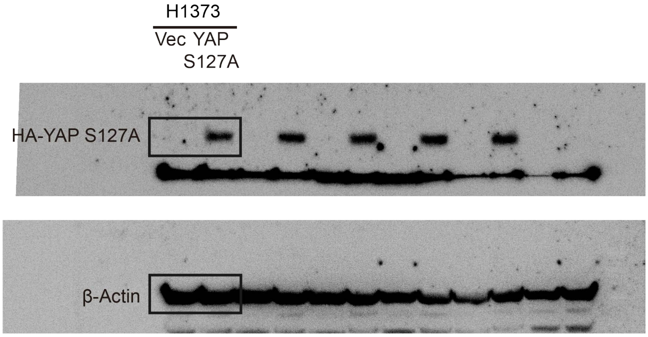

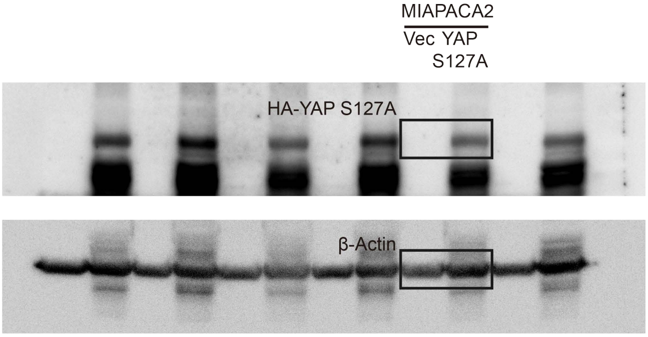

Full unedited gel for Supplemental Figure 3B

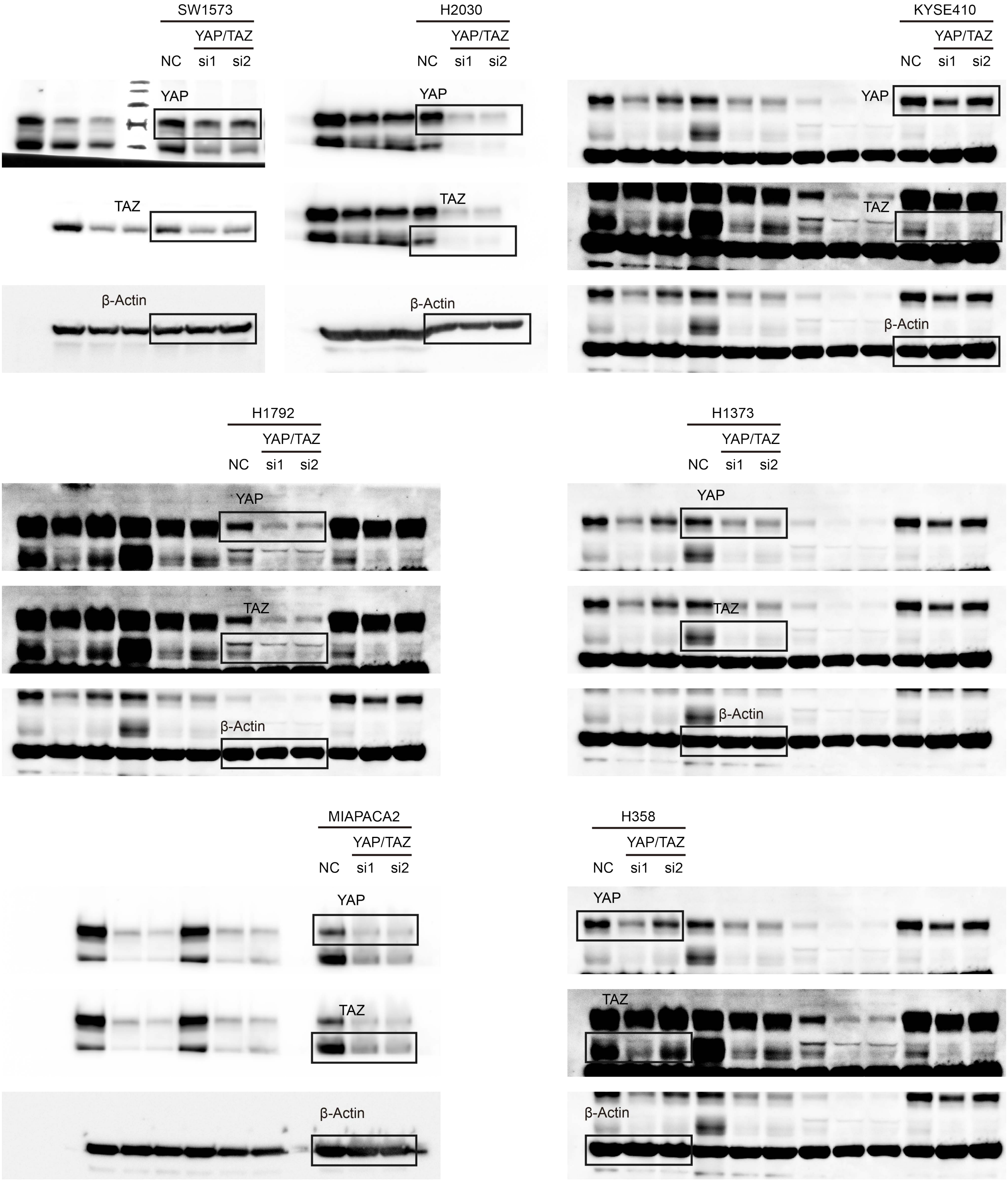

Full unedited gel for Supplemental Figure 3C

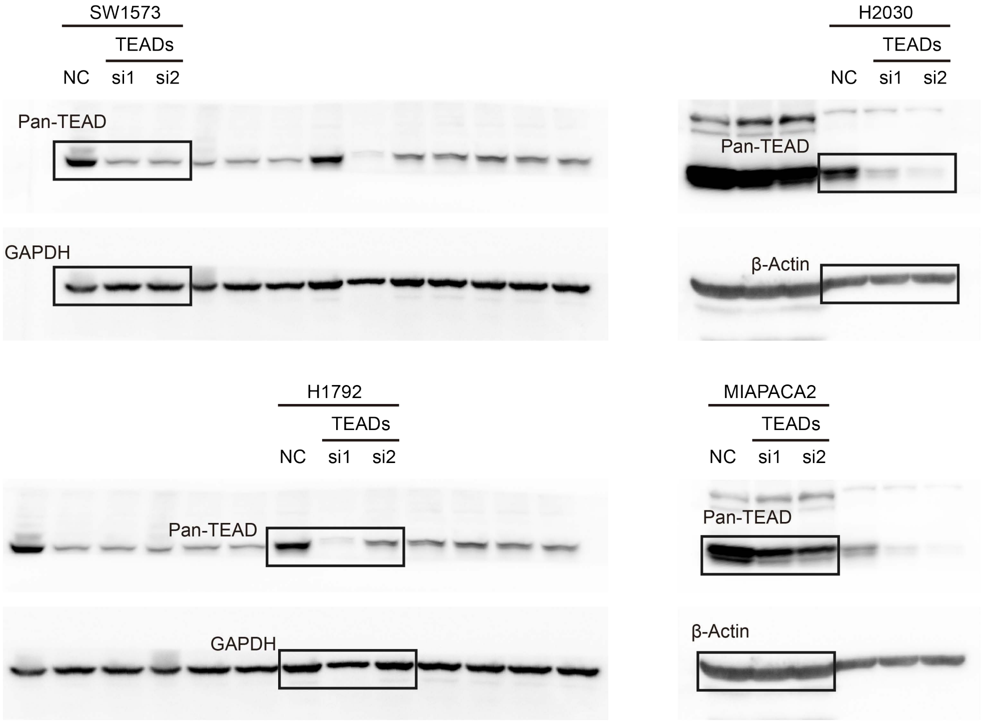

Full unedited gel for Supplemental Figure 3D

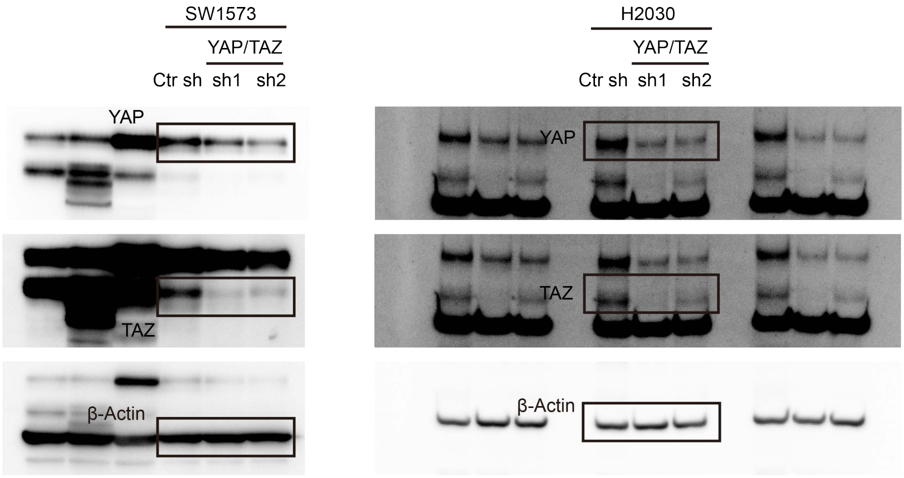

Full unedited gel for Supplemental Figure 3E

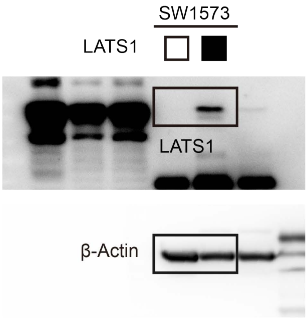

Full unedited gel for Supplemental Figure 4G

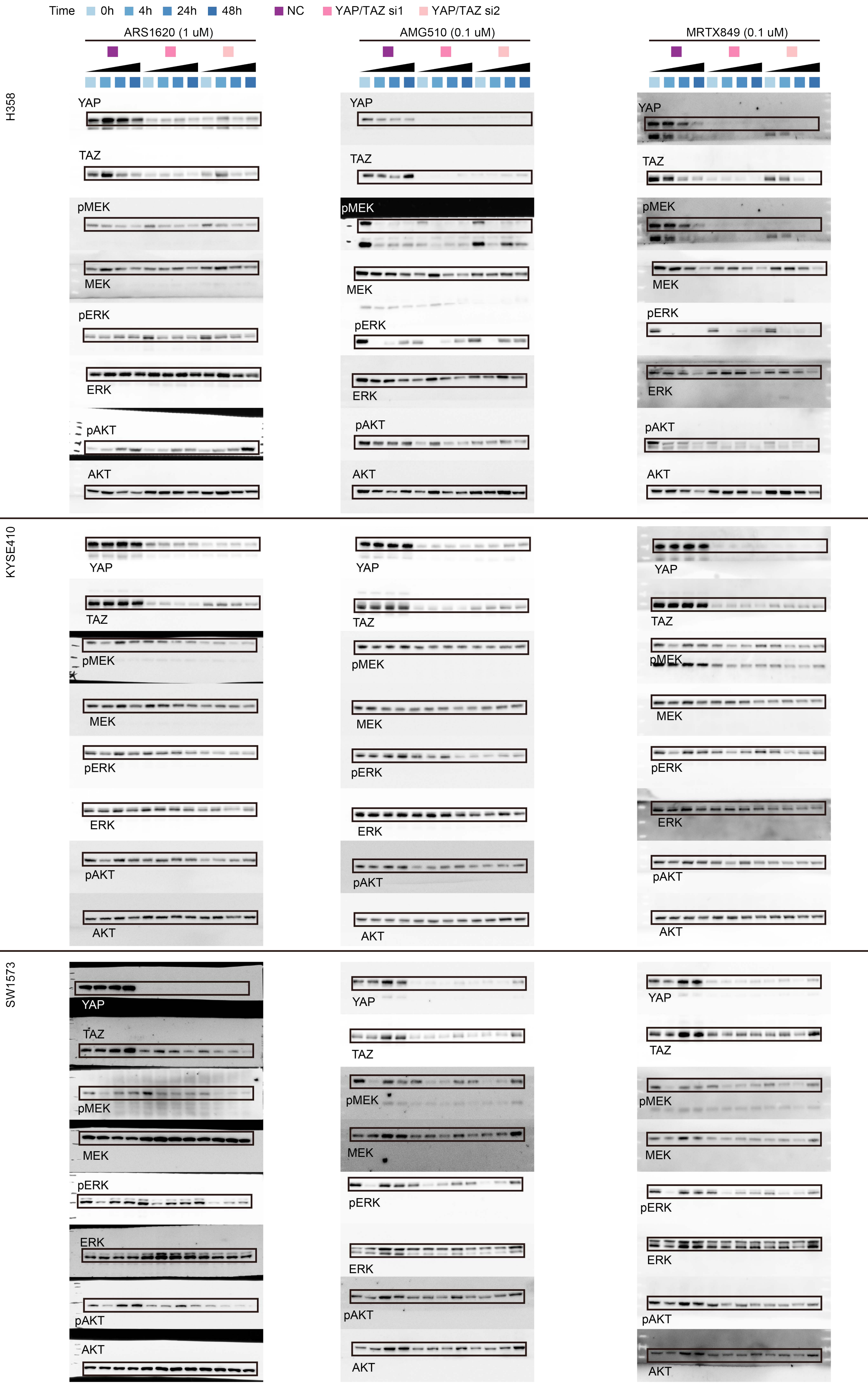

Full unedited gel for Supplemental Figure 7A

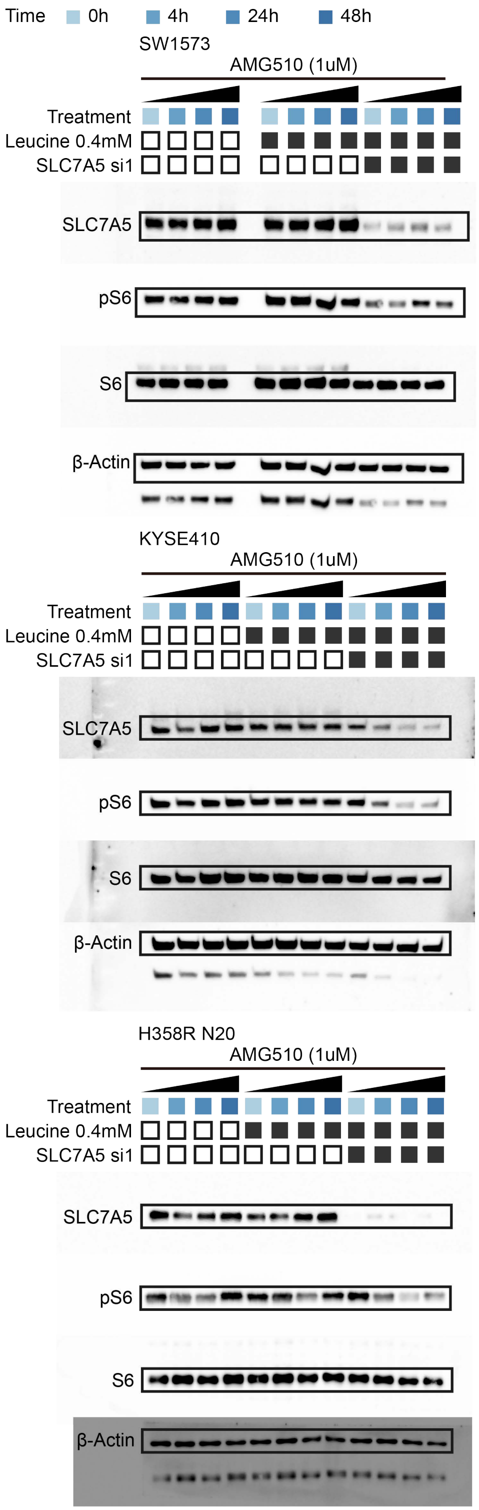

Full unedited gel for Supplemental Figure 8A

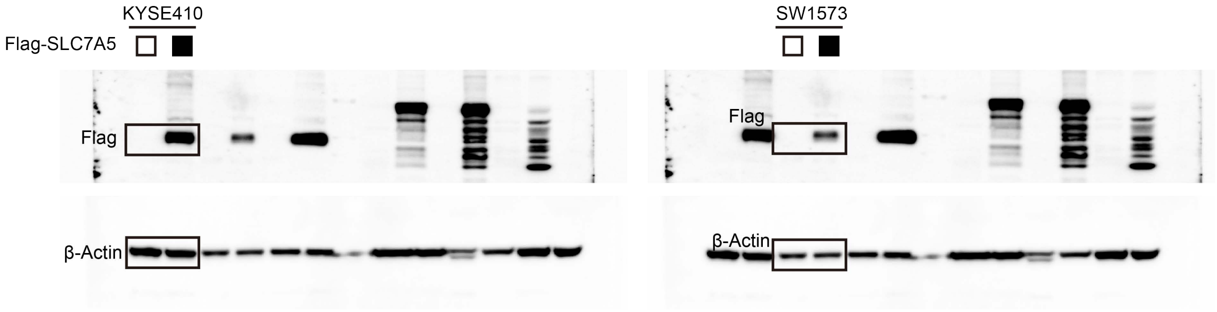

Full unedited gel for Supplemental Figure 8B

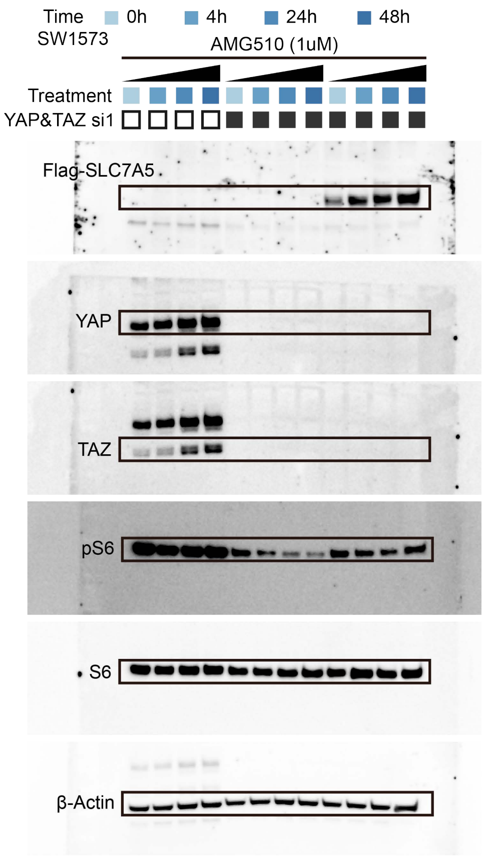

Full unedited gel for Supplemental Figure 8C

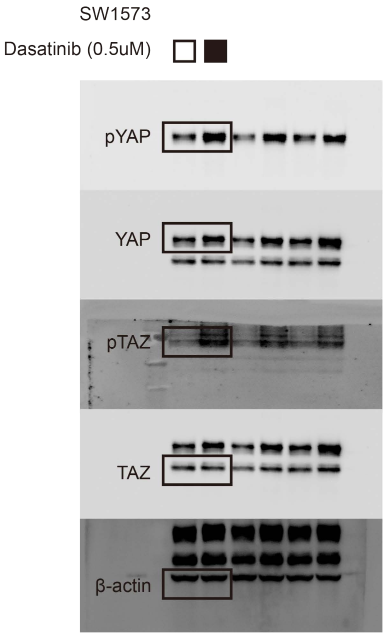

Full unedited gel for Supplemental Figure 10B

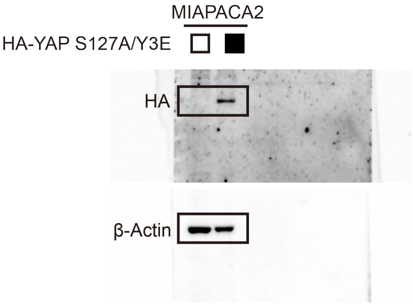

Full unedited gel for Supplemental Figure 10E

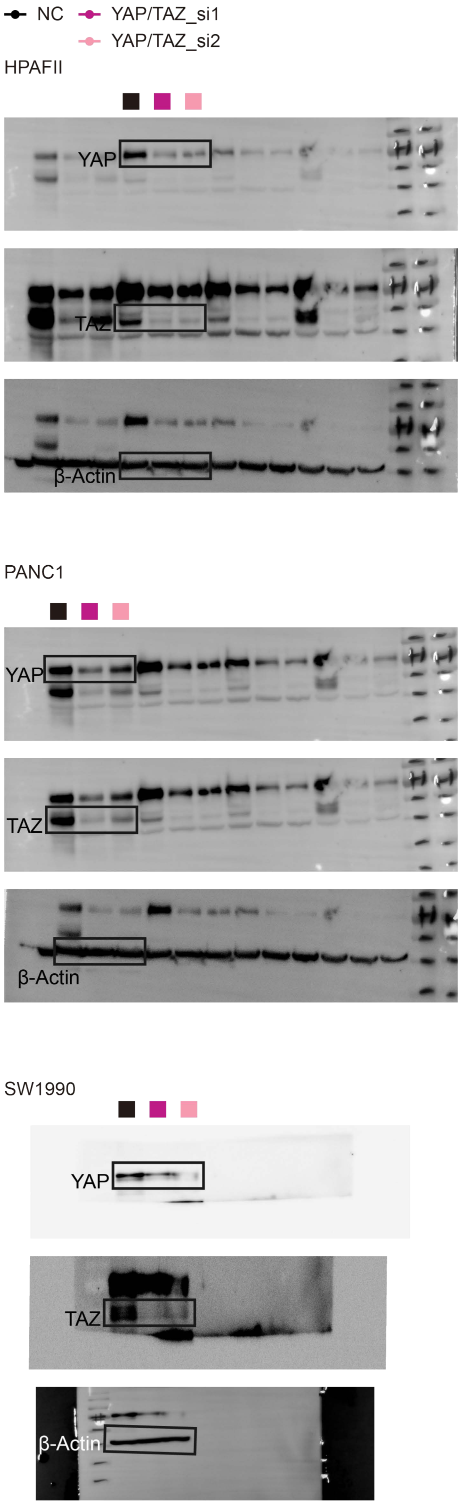

Full unedited gel for Supplemental Figure 12B

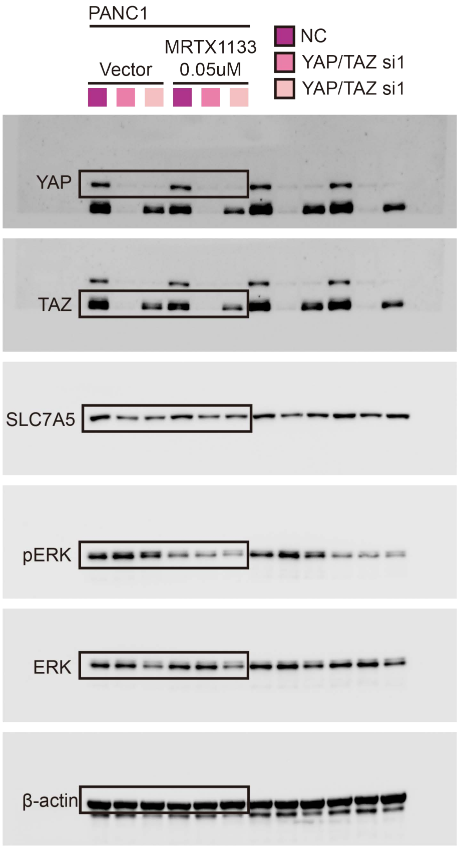

Full unedited gel for Supplemental Figure 13A

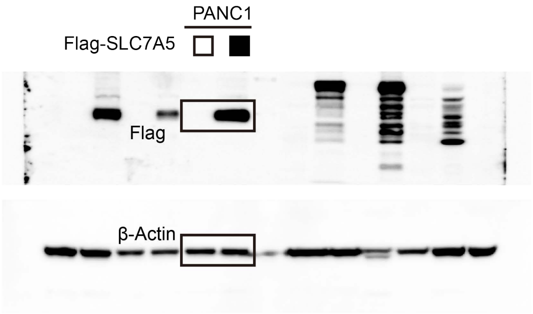

Full unedited gel for Supplemental Figure 13B
